# Author Correction: An all-in-one tetrazine reagent for cysteine-selective labeling and bioorthogonal activable prodrug construction

**DOI:** 10.1038/s41467-024-47582-0

**Published:** 2024-04-12

**Authors:** Xinyu He, Jie Li, Xinxin Liang, Wuyu Mao, Xinglong Deng, Meng Qin, Hao Su, Haoxing Wu

**Affiliations:** 1 Department of Radiology and Huaxi MR Research Center (HMRRC), Functional and Molecular Imaging Key Laboratory of Sichuan Province and Frontiers Science Center for Disease Related Molecular Network, West China Hospital, Sichuan University, Chengdu, China; 2 Key Laboratory of Drug-Targeting and Drug Delivery System of the Education Ministry and Sichuan Province, Sichuan University, Chengdu, China; 3 National Chengdu Center for Safety Evaluation of Drugs, State Key Laboratory of Biotherapy, West China Hospital, Sichuan University, Chengdu, China; 4 College of Polymer Science and Engineering, State Key Laboratory of Polymer Materials Engineering, Sichuan University, Chengdu, China

**Keywords:** Chemical tools, Peptides, Drug delivery

Correction to: *Nature Communications* 10.1038/s41467-024-47188-6, published online 02 April 2024

In the Acknowledgements section of this article the grant number relating to National Natural Science Foundation of China for Haoxing Wu was incorrectly given as 2217081 and should have been 22177081. The original article has been corrected.

