# Supplementary material for: An all-in-one tetrazine reagent for cysteine-selective labeling and bioorthogonal activable prodrug construction
Source: Nat Commun. 2024 Apr 2;15:2831. doi: 10.1038/s41467-024-47188-6 (PMC10987521; doi:10.1038/s41467-024-47188-6)
Supplement: Supplementary file 1 — Supplementary Information [file 41467_2024_47188_MOESM1_ESM.pdf]

Supplementary Information

An All-in-One Tetrazine Reagent for Cysteine-Selective Labeling and Bioorthogonal Activable Prodrug Construction

Xinyu He[1], Jie Li[1], Xinxin Liang[1], Wuyu Mao[1], Xinglong Deng[2], Meng Qin[3], Hao Su[4], and Haoxing Wu[1,2]*

[1] Department of Radiology and Huaxi MR Research Center (HMRRC), Functional and Molecular Imaging Key Laboratory of Sichuan Province and Frontiers Science Center for Disease Related Molecular Network, West China Hospital, Sichuan University, 610041, Chengdu, China
[2] Key Laboratory of Drug-Targeting and Drug Delivery System of the Education Ministry and Sichuan Province, Sichuan University, 610041, Chengdu, China
[3] National Chengdu Center for Safety Evaluation of Drugs, State Key Laboratory of Biotherapy, West China Hospital, Sichuan University, 610041, Chengdu, China
[4] College of Polymer Science and Engineering, State Key Laboratory of Polymer Materials Engineering, Sichuan University, 610065, Chengdu, China
E-mail: haoxingwu@scu.edu.cn

Supplementary Table of Contents

Supplementary Methods.. 3
Supplementary Tables 14
Supplementary Figures... 15
Supplementary References 93

Supplementary Methods

1. General methods

All chemicals were purchased from commercial sources: Adamas-Beta, Energy Chemical, or Bide pharm, China, and were used without further purification. Phosphate buffer (PB) was prepared using deionized water (Sartorius) along with sodium phosphate monobasic dihydrate and sodium phosphate dibasic dihydrate from Sigma-Aldrich. Peptides were purchased from GL Biochem (Shanghai, China). Analytical thin layer chromatography (TLC) was conducted on 0.20 mm silica gel plates. Flash column chromatography was performed on silica gel (200–300 mesh) obtained from Qingdao Haiyang Chemical (Qingdao, China). ^{1}H and ^{13}C NMR spectra were recorded on a Bruker NMR spectrometer (^{1}H, 400 MHz; ^{13}C, 101 MHz). Chemical shifts were reported as *δ* in ppm relative to tetramethylsilane (*s*, 0 ppm). Resonance multiplicities are indicated as *s* (singlet), *d* (doublet), *t* (triplet), *q* (quartet), *dd* (doublet of doublets), *m* (multiplet), or *br* (broad). The coupling constants (*J*) are reported in Hertz (Hz), and the number of protons (n) for a given resonance is indicated as nH based on the spectral integration values. High-resolution mass spectra (HRMS) were recorded on a quadrupole time-of-flight mass spectrometer (micrO-TOF-QII, Bruker) with electrospray ionization (ESI). High-performance liquid chromatography-mass spectrometry (HPLC–MS) for each peptide was recorded on a Bruker micro-TOF-QII time of flight mass spectrometer with electrospray ionization (ESI) using an Agilent 1260 Infinity HPLC system equipped with a G7129A 1260 autosampler, a G7111B 1260 Quat Pump, and a G7155A 1260 DAD detector connected to a G6125B Single Quadrupole LC/MS system with an Agilent HPLC column. H_2O (solvent A) and MeCN (solvent B), each supplemented with 0.1% trifluoroacetic acid (TFA), were used as the mobile phase, and the solvent composition was controlled by a gradient program. Low-resolution mass spectra were obtained using positive ESI in the dynamic mode over a mass range of 200–2000 *m/z* under the following conditions: temperature of drying gas = 350 °C, the flow rate of drying gas = 12 L/min, the pressure of nebulizer gas = 35 psi, capillary voltage = 3000 V, and fragment voltage = 70 V. Ultraviolet–visible (UV–Vis) absorption spectra were recorded on a Quawell scientific Q6000+ microvolume spectrophotometer. Fluorescence spectra were acquired on a wavelength-calibrated FluoroMax-4 fluorometer (Horiba Jobin Yvon, Kyoto, Japan).

2. Preparation of voTz analogues

*2.1 Preparation of voTz **1***

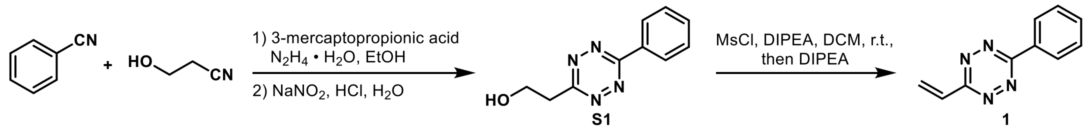

*2.1.1 Synthesis of **S1***

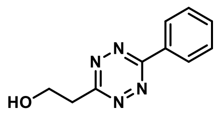

S1

Benzonitrile (206 mg, 2 mmol), 3-hydroxypropanenitrile (548 µL, 8 mmol), 3-mercaptopropionic acid (174 µL, 3 mmol), ethanol (EtOH, 0.6 mL), and hydrazine hydrate ($N_2H_4 \cdot H_2O$, 1.2 mL, 24 mmol) were added to a 10 mL reaction tube containing a magnetic stir bar. The mixture was stirred at 40 °C overnight under an argon atmosphere. Subsequently, the reaction solution was cooled to 0 °C, and sodium nitrite (1.4 g, 20 mmol) dissolved in ice water (50 mL) and 1 M HCl were slowly added, resulting in a bright red solution and the evolution of gas. The addition of 1 M HCl was continued until the gas evolution ceased, and the pH value reached 2.0–3.0. After stirring the mixture for approximately 5 min, it was extracted with dichloromethane (DCM, 50 mL × 3), dried over anhydrous Na_2SO_4, and concentrated under vacuum. The resulting residue was purified using silica gel column chromatography to afford the desired product **S1** as a pink oil (247 mg, 61%).^{1}**H NMR** (400 MHz, Chloroform-*d*) *δ* 8.69–8.42 (*m*, 2H), 7.73–7.53 (*m*, 3H), 4.31 (*dd*, *J* = 10.9, 5.4 Hz, 2H), 3.63 (*t*, *J* = 5.8 Hz, 2H), 2.69 (*t*, *J* = 5.2 Hz, 1H). ^{13}**C NMR** (101 MHz, Chloroform-*d*) *δ* 168.29, 164.57, 132.79, 131.59, 129.32, 128.01, 60.08, 37.51. **HRMS** $[M+H]^+$ *m/z* calcd. for $[C_{10}H_{11}N_4O]^+$ 203.0927, found 203.0929.

*2.1.2 Synthesis of **1***

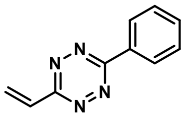

1

To a solution of **S1** (181 mg, 0.89 mmol) and N,N-Diisopropylethylamine (DIPEA, 233 µL, 1.34 mmol) in 4 mL DCM, methylsulfonyl chloride (MsCl, 103 µL, 1.34 mmol) was added dropwise at 0 °C under an argon atmosphere. The mixture was stirred at room temperature, and the reaction progress was monitored by TLC. After the complete conversion of **S1** to the methanesulfonate intermediate, DIPEA (233 µL, 1.34 mmol) was added. After stirring the mixture at room temperature for approximately 1 h, H_2O (20 mL) was then added, and the mixture was extracted with DCM (20 mL × 3). The combined organic phases were washed with brine, dried over Na_2SO_4, and concentrated under vacuum. The resulting residue was purified using silica gel column chromatography to afford the desired product **1** as a red solid (143.2 mg, 97%). **^{1}H NMR** (400 MHz, Chloroform-*d*) *δ* 8.70–8.56 (*m*, 2H), 7.70–7.53 (*m*, 3H), 7.22 (*dd*, *J* = 17.6, 10.9 Hz, 1H), 7.04 (*dd*, *J* = 17.6, 1.0 Hz, 1H), 6.07 (*dd*, *J* = 10.8, 1.0 Hz, 1H). **^{13}C NMR** (101 MHz, Chloroform-*d*) *δ* 163.88, 163.78, 132.74, 131.77, 130.46, 129.30, 128.07, 127.42. **HRMS** $[M{-}H]^-$ *m/z* calcd. for $[C_{10}H_7N_4]^-$ 183.0676, found 183.0671.

*2.2 Preparation of voTz **3−5***

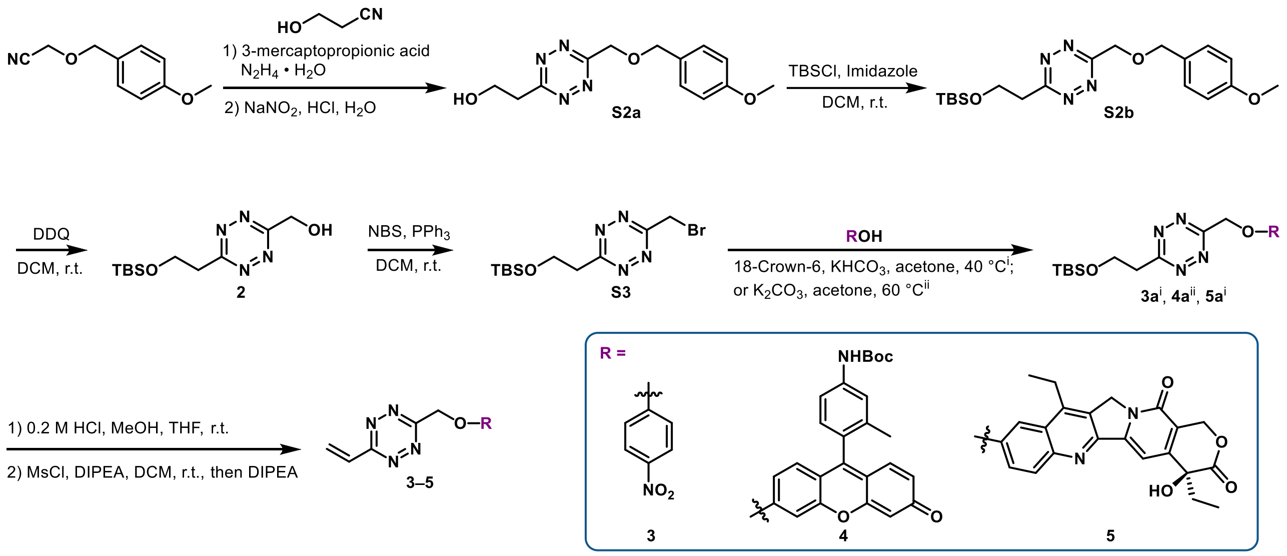

*2.2.1 Synthesis of **S2a***

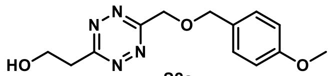

S2a

2-((4-methoxybenzyl)oxy)acetonitrile (6 g, 33.9 mmol), 3-hydroxypropanenitrile (5.79 mL, 84.75 mmol), 3-mercaptopropionic acid (2.8 mL, 33.9 mmol), EtOH (15 mL), and $N_2H_4{\cdot}H_2O$ (33.5 mL, 678 mmol) were added to a 200 mL reaction flask containing a magnetic stir bar. The mixture was stirred at room temperature overnight under an argon atmosphere. Subsequently, the reaction solution was cooled to 0 °C, and sodium nitrite (35 g, 508 mmol) dissolved in ice water (50 mL) and 1 M HCl were slowly added, resulting in a bright red solution and the evolution of gas. The addition of 1 M HCl was continued until the gas evolution ceased, and the pH value reached 2.0–3.0. After stirring the mixture for approximately 5 min, it was extracted with DCM (50 mL × 3), dried over anhydrous Na_2SO_4, and concentrated under vacuum. The resulting residue was purified using silica gel column chromatography to afford the desired product **S2a** as a red oil (2.46 g, 26%). **^{1}H NMR** (400 MHz, Chloroform-*d*) δ 7.37–7.27 (m, 2H), 6.91–6.84 (m, 2H), 5.08 (s, 2H), 4.72 (s, 2H), 4.24 (t, *J* = 5.8 Hz, 2H), 3.80 (s, 3H), 3.58 (t, *J* = 5.8 Hz, 2H). **^{13}C NMR** (101 MHz, Chloroform-*d*) δ 169.38, 167.06, 159.58, 129.90, 129.00, 113.97, 73.52, 69.29, 59.93, 55.31, 37.62. **HRMS** $[M{+}Na]^+$ *m/z* calcd. for $[C_{13}H_{16}N_4NaO_3]^+$ 299.1115, found 299.1109.

*2.2.2 Synthesis of **S2b***

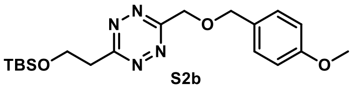

S2b

To a solution of **S2a** (2.2 g, 7.97 mmol) and imidazole (705 mg, 10.36 mmol) in 40 mL anhydrous DCM under argon atmosphere was added TBSCl (1.4 g, 9.56 mmol) in several portions. The reaction mixture was monitored by TLC and stirred at room temperature for approximately 2 h, then diluted with DCM (100 mL). The resulting mixture was washed with water (50 mL × 3) and brine (50 mL), dried over anhydrous Na_2SO_4, and concentrated under vacuum. The resulting residue was purified using silica gel column chromatography to afford the desired product **S2b** as a red oil (2.9 g, 91%). **^{1}H NMR** (400 MHz, Chloroform-*d*) δ 7.39–7.29 (m, 2H), 6.94–6.82 (m, 2H), 5.08 (s, 2H), 4.71 (s, 2H), 4.23 (t, *J* = 6.4 Hz, 2H), 3.81 (s, 3H), 3.54 (t, *J* = 6.4 Hz, 2H), 0.79 (s, 9H), -0.01 (s, 6H). **^{13}C NMR** (101 MHz, Chloroform-*d*) δ 169.31, 166.76, 159.57, 129.85, 129.09, 113.95, 73.31, 69.28, 61.10, 55.30, 38.39, 25.72, 18.14, -5.47. **HRMS** $[M{+}Na]^+$ *m/z* calcd. for $[C_{19}H_{30}N_4NaO_3Si]^+$ 413.1979, found 413.1972.

*2.2.3 Synthesis of **2***

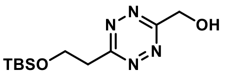

2

To a solution of **S2b** (2.8 g, 7.18 mmol) in 40 mL DCM was added 4,5-dichloro-3,6-dioxocyclohexa-1,4-diene-1,2-dicarbonitrile (DDQ, 2 g, 8.8 mmol). The reaction mixture was monitored by TLC and stirred at room temperature for approximately 4 h, then diluted with DCM (50 mL). The resulting mixture was washed with saturated sodium bicarbonate solution (50 mL × 3) and brine (50 mL × 1), dried over anhydrous Na_2SO_4, and concentrated under vacuum. The resulting residue was purified using silica gel column chromatography to afford the desired product **2** as a red oil (1.6 g, 83%). **^{1}H NMR** (400 MHz, Chloroform-*d*) δ 5.27 (d, *J* = 5.3 Hz, 2H), 4.24 (t, *J* = 6.3 Hz, 2H), 3.56 (t, *J* = 6.3 Hz, 2H), 3.24 (t, *J* = 6.3 Hz, 1H), 0.78 (s, 9H), -0.02 (s, 6H). **^{13}C NMR** (101 MHz, Chloroform-*d*) δ 169.77, 167.58, 62.70, 61.07, 38.37, 25.69, 18.13, -5.49. **HRMS** $[M{+}H]^+$ *m/z* calcd. for $[C_{11}H_{23}N_4O_2Si]^+$ 271.1585, found 271.1581.

*2.2.4 Synthesis of **S3***

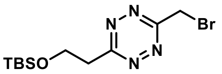

S3

To a solution of **2** (128 mg, 0.47mmol) and N-bromosuccinimide (NBS, 143 mg, 0.81 mmol) in 4 mL anhydrous DCM under argon atmosphere was added triphenylphosphine (PPh_3, 186 mg, 0.71 mmol) in several portions. The reaction mixture was monitored by TLC and stirred at room temperature for approximately 30 min, then diluted with DCM (20 mL). The resulting mixture was washed with water (20 mL × 3) and dried over anhydrous Na_2SO_4, and concentrated under vacuum. The resulting residue was purified using silica gel column chromatography to afford the desired product **S3** as a red oil (131 mg, 83%). **^{1}H NMR** (400 MHz, Chloroform-*d*) δ 4.93 (s, 2H), 4.23 (t, *J* = 6.3 Hz, 2H), 3.56 (t, *J* = 6.2 Hz, 2H), 0.77 (s, 9H), -0.03 (s, 6H). **^{13}C NMR** (101 MHz, Chloroform-*d*) δ 168.82, 167.54, 61.00, 38.33, 27.54, 25.70, 18.10, -5.51. **HRMS** $[M{+}H]^+$ *m/z* calcd. for $[C_{11}H_{22}BrN_4OSi]^+$ 333.0741, found 333.0740.

*2.2.5 Synthesis of **3a***

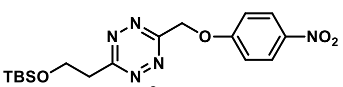

3a

To a mixture of 4-nitrophenol (21 mg, 0.15 mmol), $KHCO_3$ (45.3 mg, 0.45 mmol), 18-crown-6 (10 mg, 0.04 mmol) in acetone (5 mL) was added **S3** (60 mg, 0.18 mmol). The mixture was further stirred at 40 °C, and the reaction progress was monitored by TLC. After the reaction was completed (about 4 h), H_2O (20 mL) was then added, and the mixture was extracted with ethyl acetate (20 mL × 3). The combined organic phases were washed with brine, dried over anhydrous Na_2SO_4, and concentrated under vacuum. The resulting residue was purified using silica gel column chromatography to afford the desired product **3a** as a red solid (49 mg, 83%). **^{1}H NMR** (400 MHz, Chloroform-*d*) δ 8.23 (d, *J* = 9.2 Hz, 2H), 7.14 (d, *J* = 9.3 Hz, 2H), 5.76 (s, 2H), 4.25 (t, *J* = 6.2 Hz, 2H), 3.59 (t, *J* = 6.2 Hz, 2H), 0.75 (s, 9H), -0.04 (s, 6H). **^{13}C NMR** (101 MHz, Chloroform-*d*) δ 170.06, 164.99, 162.62, 142.46, 126.04, 115.02, 68.09, 60.97, 38.43, 25.66, 18.08, -5.53. **HRMS** $[M{+}H]^+$ *m/z* calcd. for $[C_{17}H_{26}N_5O_4Si]^+$ 392.1749, found 392.1743.

*2.2.6 Synthesis of **3***

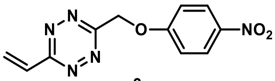

3

To a solution of **3a** (63 mg, 0.16 mmol) in THF (8 mL) and methanol (2 mL) was added 0.2 M HCl aqueous (8 mL). The mixture was further stirred at room temperature, and the reaction progress was monitored by TLC. After the reaction was completed, the mixture was diluted with 20 mL ethyl acetate, $NaHCO_3$ aqueous solution and 10 mL water was added. The organic phase was separated, and water phase was further extracted with ethyl acetate (20 mL × 3). The combined organic phase was washed with brine, dried over Na_2SO_4, and concentrated under vacuum. The resulting residue was purified using silica gel column chromatography to afford the tetrazine intermediate as a red solid (39 mg). To a solution of intermediate (39 mg, 0.14 mmol) and DIPEA (36.5 μL, 0.21 mmol) in 4 mL anhydrous DCM, MsCl (16.3 μL, 0.21 mmol) was added dropwise at 0 °C under an argon atmosphere. The mixture was stirred at room temperature, and the reaction progress was monitored by TLC. After the complete conversion to the methanesulfonate intermediate, DIPEA (36.5 μL, 0.21 mmol) was added, the mixture was further stirred at room temperature, and the reaction progress was monitored by TLC. After the reaction was completed, H_2O (20 mL) was then added, and the mixture was extracted with DCM (20 mL × 3). The combined organic phases were washed with brine, dried over Na_2SO_4, and concentrated under vacuum. The resulting residue was purified using silica gel column chromatography to afford the desired product **3** as a red solid (32.6 mg, 79% over two steps). **^{1}H NMR** (400 MHz, Chloroform-*d*) δ 8.30–8.20 (m, 2H), 7.25–7.04 (m, 4H), 6.16 (dd, *J* = 10.6, 1.3 Hz, 1H), 5.76 (s, 2H). **^{13}C NMR** (101 MHz, Chloroform-*d*) δ 165.06, 164.56, 162.63, 142.50, 130.02, 129.55, 126.07, 115.03, 68.09. **HRMS** $[M\text{+Na}]^+$ *m/z* calcd. for $[C_{11}H_9N_5NaO_3]^+$ 282.0598, found 282.0588.

*2.2.7 Synthesis of **4a***

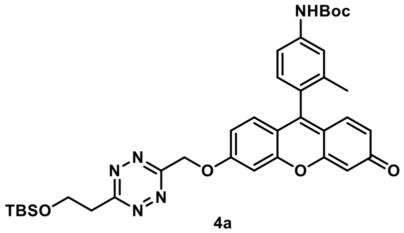

4a

To a mixture of *tert*-butyl (4-(6-hydroxy-3-oxo-3*H*-xanthen-9-yl)-3-methylphenyl)carbamate (90 mg, 0.22 mmol), K_2CO_3 (50.7 mg, 0.37 mmol), in acetone (20 mL) was added **S3** (144 mg, 0.43 mmol). The mixture was further stirred on reflux at 60 °C, and the reaction progress was monitored by TLC. After the reaction was completed, H_2O (20 mL) was then added, and the mixture was extracted with ethyl acetate (20 mL × 3). The combined organic phases were washed with brine, dried over Na_2SO_4, and concentrated under vacuum. The resulting residue was purified using silica gel column chromatography to afford the desired product **4a** as an orange solid (98 mg, 68%). **^{1}H NMR** (400 MHz, Chloroform-*d*) δ 7.55 (d, *J* = 2.1 Hz, 1H), 7.38 (dd, *J* = 8.3, 2.2 Hz, 1H), 7.16 (d, *J* = 2.4 Hz, 1H), 7.11 (dd, *J* = 8.6, 1.9 Hz, 2H), 7.05 (d, *J* = 9.7 Hz, 1H), 6.96 (dd, *J* = 8.9, 2.4 Hz, 1H), 6.77 (s, 1H), 6.65 (d, *J* = 9.7 Hz, 1H), 6.55 (s, 1H), 5.82 (s, 2H), 4.29 (t, *J* = 6.2 Hz, 2H), 3.63 (t, *J* = 6.2 Hz, 2H), 2.08 (s, 3H), 1.59 (s, 9H), 0.78 (s, 9H), -0.00 (s, 6H). **^{13}C NMR** (101 MHz, Chloroform-*d*) δ 185.68, 170.03, 165.03, 162.26, 158.84, 154.41, 152.76, 149.28, 139.79, 137.32, 130.74, 130.17, 129.88, 126.58, 120.13, 119.13, 116.08, 115.56, 113.71, 105.90, 101.65, 81.04, 68.07, 60.98, 38.43, 28.34, 25.65, 19.92, 18.07, -5.53. **HRMS** $[M\text{+H}]^+$ *m/z* calcd. for $[C_{36}H_{44}N_5O_6Si]^+$ 670.3055, found 670.3050.

*2.2.8 Synthesis of **4***

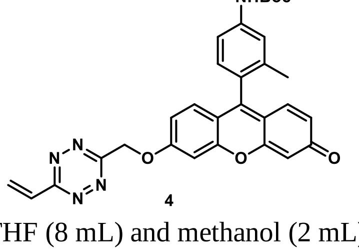

4

To a solution of **4a** (80 mg, 0.12 mmol) in THF (8 mL) and methanol (2 mL) was added 0.2 M HCl aqueous (5.25 mL). The mixture was further stirred at room temperature, and the reaction progress was monitored by TLC. After the reaction was completed, the mixture was diluted with 20 mL DCM, $NaHCO_3$ aqueous solution and 10 mL water was added. The

organic phase was separated, and water phase was further extracted with DCM (20 mL × 3). The combined organic phase was washed with brine, dried over Na_2SO_4, and concentrated under vacuum. The resulting residue was purified using silica gel column chromatography to afford the tetrazine intermediate as an orange solid (59 mg).
To a solution of intermediate (59 mg, 0.11 mmol) and DIPEA (27.6 µL, 0.16 mmol) in 4 mL anhydrous DCM, MsCl (12.3 µL, 0.16 mmol) was added dropwise at 0 °C under an argon atmosphere. The mixture was stirred at room temperature, and the reaction progress was monitored by TLC. After the complete conversion to the methanesulfonate intermediate, DIPEA (27.6 µL, 0.16 mmol) was added, the mixture was further stirred at room temperature, and the reaction progress was monitored by TLC. After the reaction was completed, H_2O (20 mL) was then added, and the mixture was extracted with DCM (20 mL × 3). The combined organic phases were washed with brine, dried over Na_2SO_4, and concentrated under vacuum. The resulting residue was purified using silica gel column chromatography to afford the desired product **4** as an orange solid (46.4 mg, 73% over two steps). **^{1}H NMR** (400 MHz, Chloroform-*d*) δ 7.52 (d, *J* = 2.1 Hz, 1H), 7.35 (dd, *J* = 8.3, 2.2 Hz, 1H), 7.22 (dd, *J* = 17.6, 10.7 Hz, 1H), 7.13 (dd, *J* = 5.2, 1.9 Hz, 2H), 7.09–7.06 (m, 2H), 7.00 (d, *J* = 9.7 Hz, 1H), 6.95–6.86 (m, 2H), 6.58 (dd, *J* = 9.7, 2.0 Hz, 1H), 6.46 (d, *J* = 1.9 Hz, 1H), 6.16 (dd, *J* = 10.6, 1.3 Hz, 1H), 5.78 (s, 2H), 2.04 (s, 3H), 1.56 (s, 9H). **^{13}C NMR** (101 MHz, Chloroform-*d*) δ 185.86, 165.04, 164.62, 162.18, 158.84, 154.37, 152.78, 149.08, 139.77, 137.33, 130.73, 130.26, 130.03, 129.88, 129.49, 126.60, 120.14, 119.15, 116.09, 115.57, 113.59, 105.93, 101.71, 81.04, 68.07, 28.34, 19.92. **HRMS** $[M+H]^+$ *m/z* calcd. for $[C_{30}H_{28}N_5O_5]^+$ 538.2085, found 538.2079.

*2.2.9 Synthesis of **5a***

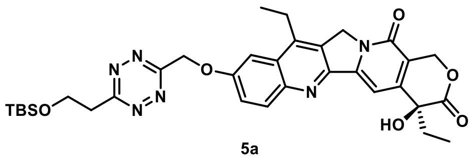

5a

To a mixture of (S)-4,11-diethyl-4,9-dihydroxy-1,12-dihydro-14H-pyrano[3',4':6,7]indolizino[1,2-b]quinoline-3,14(4H)-dione (26 mg, 0.07 mmol), $KHCO_3$ (20 mg, 0.2 mmol), 18-crown-6 (4.4 mg, 0.02 mmol) in acetone (20 mL) was added **S3** (26.4 mg, 0.08 mmol). The mixture was further stirred at 40 °C, and the reaction progress was monitored by TLC. After the reaction was completed, H_2O (20 mL) was then added, and the mixture was extracted with ethyl acetate (20 mL × 3). The combined organic phases were washed with brine, dried over anhydrous Na_2SO_4, and concentrated under vacuum. The resulting residue was purified using silica gel column chromatography to afford the desired product **5a** as a red solid (27.9 mg, 65%). **^{1}H NMR** (400 MHz, DMSO-*d*$_6$) δ 8.09 (d, *J* = 9.2 Hz, 1H), 7.75 (d, *J* = 2.8 Hz, 1H), 7.60 (dd, *J* = 9.2, 2.7 Hz, 1H), 7.27 (s, 1H), 6.50 (s, 1H), 6.01 (s, 2H), 5.42 (s, 2H), 5.27 (s, 2H), 4.17 (t, *J* = 6.2 Hz, 2H), 3.49 (t, *J* = 6.2 Hz, 2H), 3.17 (q, *J* = 7.6 Hz, 2H), 1.85 (q, *J* = 7.2 Hz, 2H), 1.28 (d, *J* = 7.7 Hz, 3H), 0.88 (t, *J* = 7.3 Hz, 3H), 0.65 (s, 9H), -0.11 (s, 6H). **^{13}C NMR** (101 MHz, DMSO-*d*$_6$) δ 172.99, 169.62, 166.06, 157.30, 156.95, 150.53, 150.50, 146.64, 145.23, 144.65, 132.14, 128.93, 128.16, 122.77, 118.88, 104.72, 96.61, 72.87, 68.14, 65.72, 61.21, 49.97, 38.33, 30.72, 29.48, 25.94, 18.12, 13.96, 8.22, -5.15. **HRMS** $[M+H]^+$ *m/z* calcd. for $[C_{33}H_{41}N_6O_6Si]^+$ 645.2851, found 645.2850.

*2.2.10 Synthesis of **5***

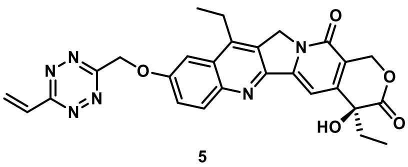

5

To a solution of **5a** (29 mg, 0.04 mmol) in THF (4 mL) and methanol (1 mL) was added 1 M HCl aqueous (0.45 mL). The mixture was further stirred at room temperature, and the reaction progress was monitored by TLC. After the reaction was completed, the mixture was diluted with 5 mL ethyl acetate, $NaHCO_3$ aqueous solution and 10 mL water was added. The organic phase was separated, and water phase was further extracted with ethyl acetate (10 mL × 3). The combined organic phase was washed with brine, dried over Na_2SO_4, and concentrated under vacuum. The resulting residue was purified using silica gel column chromatography to afford the tetrazine intermediate as a red solid (18 mg).
To a solution of intermediate (18 mg, 0.03 mmol) and DIPEA (8.69 µL, 0.05 mmol) in 1 mL anhydrous THF, MsCl (4 µL, 0.05 mmol) was added dropwise at 0 °C under an argon atmosphere. The mixture was stirred at room temperature, and the reaction progress was monitored by TLC. After the complete conversion to the methanesulfonate intermediate, DIPEA (8.69 µL, 0.05 mmol) was added, the mixture was further stirred at room temperature, and the reaction progress was monitored by TLC. After the reaction was completed, H_2O (10 mL) was then added, and the mixture was extracted

with DCM (10 mL × 3). The combined organic phases were washed with brine, dried over Na_2SO_4, and concentrated under vacuum. The resulting residue was purified using silica gel column chromatography to afford the desired product **5** as a red solid (10.7 mg, 47% over two steps). **^{1}H NMR** (400 MHz, Chloroform-*d*) δ 8.21–8.13 (m, 1H), 7.63–7.54 (m, 3H), 7.24–7.14 (m, 1H), 7.09 (dd, *J* = 17.5, 1.3 Hz, 1H), 6.15 (dd, *J* = 10.6, 1.3 Hz, 1H), 5.86 (s, 2H), 5.75 (d, *J* = 16.2 Hz, 1H), 5.35–5.20 (m, 3H), 3.77 (s, 1H), 3.15 (q, *J* = 7.7 Hz, 2H), 1.98–1.83 (m, 2H), 1.38 (t, *J* = 7.6 Hz, 3H), 1.03 (t, *J* = 7.3 Hz, 3H). **^{13}C NMR** (101 MHz, Chloroform-*d*) δ 174.01, 165.12, 164.98, 157.71, 157.01, 150.36, 150.23, 147.21, 145.90, 144.07, 132.62, 130.07, 129.42, 127.97, 127.45, 122.36, 118.06, 103.82, 97.58, 72.79, 68.14, 66.39, 49.45, 31.60, 23.21, 13.68, 7.84. **HRMS** $[M+H]^+$ *m/z* calcd. for $[C_{27}H_{25}N_6O_5]^+$ 513.1881, found 513.1877.

*2.3 Preparation of voTz **6***

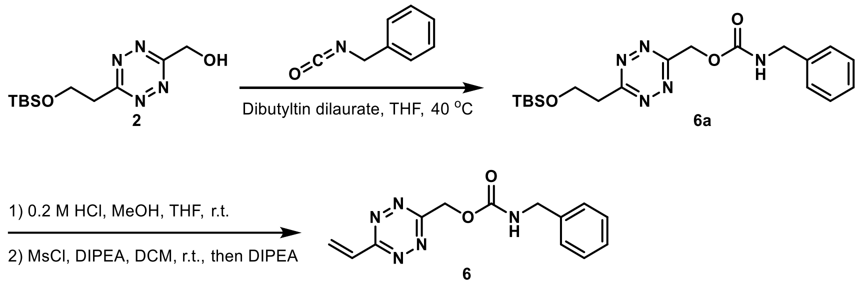

*2.3.1 Synthesis of **6a***

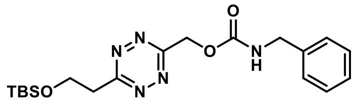

6a

To a solution of **2** (80 mg, 0.3 mmol) in 2 mL THF, (isocyanatomethyl)benzene (4.85 µL, 0.06 mmol) and dibutyltin dilaurate were added at 40 °C under an argon atmosphere. The mixture was stirred at 40 °C, and the reaction progress was monitored by TLC. After the reaction was completed, H_2O (10 mL) was then added, and the mixture was extracted with DCM (10 mL × 3). The combined organic phases were washed with brine, dried over Na_2SO_4, and concentrated under vacuum. The resulting residue was purified using silica gel column chromatography to afford the desired product **6a** as a red oil (110 mg, 91%). **^{1}H NMR** (400 MHz, Chloroform-*d*) δ 7.39–7.27 (m, 5H), 5.71 (s, 2H), 4.42 (d, *J* = 6.0 Hz, 2H), 4.24 (t, *J* = 6.4 Hz, 2H), 3.56 (t, *J* = 6.4 Hz, 2H), 0.80 (s, 9H), -0.01 (s, 6H). **^{13}C NMR** (101 MHz, Chloroform-*d*) δ 169.53, 165.76, 155.57, 137.90, 128.77, 127.68, 127.55, 63.53, 61.08, 45.35, 38.39, 25.73, 18.15, -5.48. **HRMS** $[M+H]^+$ *m/z* calcd. for $[C_{19}H_{30}N_5O_3Si]^+$ 404.2112, found 404.2107.

*2.3.2 Synthesis of **6***

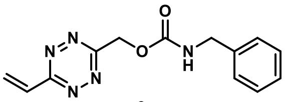

6

To a solution of **6a** (25 mg, 0.06 mmol) in THF (15 mL) and methanol (4 mL) was added 0.2 M HCl aqueous (3.1 mL). The mixture was further stirred at room temperature, and the reaction progress was monitored by TLC. After the reaction was completed, the mixture was diluted with 30 mL DCM, $NaHCO_3$ aqueous solution and 10 mL water was added. The organic phase was separated, and water phase was further extracted with DCM (30 mL × 3). The combined organic phase was washed with brine, dried over Na_2SO_4, and concentrated under vacuum. The resulting residue was purified using silica gel column chromatography to afford the tetrazine intermediate as a red oil (12 mg).

To a solution of intermediate (12 mg, 0.04 mmol) and DIPEA (10.8 µL, 0.06 mmol) in 1 mL anhydrous DCM, MsCl (4.9 µL, 0.06 mmol) was added dropwise at 0 °C under an argon atmosphere. The mixture was stirred at room temperature, and the reaction progress was monitored by TLC. After the complete conversion to the methanesulfonate intermediate, DIPEA (10.8 µL, 0.06 mmol) was added, the mixture was further stirred at room temperature, and the reaction progress was monitored by TLC. After the reaction was completed, H_2O (10 mL) was then added, and the mixture was extracted with DCM (10 mL × 3). The combined organic phases were washed with brine, dried over Na_2SO_4, and concentrated under vacuum. The resulting residue was purified using silica gel column chromatography to afford the desired product **6** as a red oil (7 mg, 50% over two steps). **^{1}H NMR** (400 MHz, Chloroform-*d*) δ 7.40–7.30 (m, 5H), 7.21–7.13 (m, 1H), 7.04 (dd, *J* = 17.6, 1.2 Hz, 1H), 6.10 (dd, *J* = 10.8, 1.2 Hz, 1H), 5.71 (s, 2H), 4.42 (d, *J* = 6.0 Hz,

2H). **^{13}C NMR** (101 MHz, Chloroform-*d*) δ 165.36, 164.92, 155.60, 137.87, 130.21, 128.77, 128.55, 127.69, 127.55, 63.58, 45.36. **HRMS** [*M*+H]$^+$ *m/z* calcd. for [$C_{13}H_{14}N_5O_2$]$^+$ 272.1142, found 272.1140.

2.4 Preparation of voTz ***7***

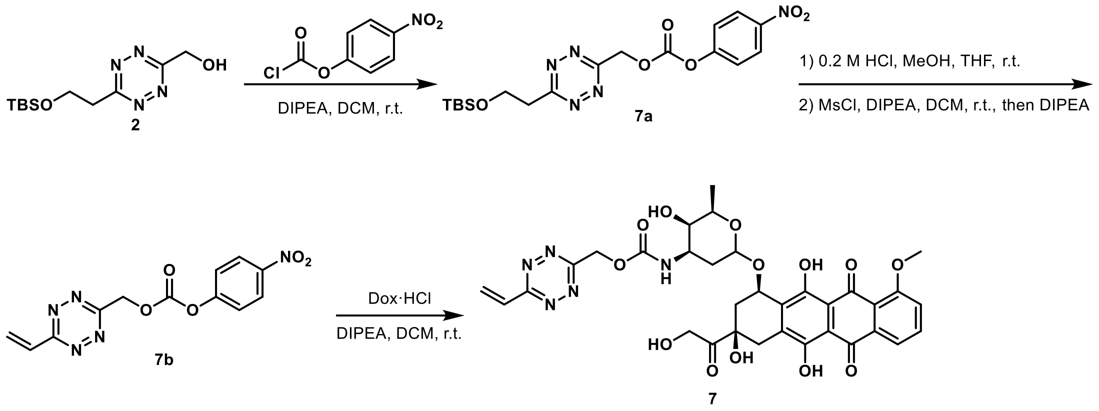

2.4.1 Synthesis of ***7a***

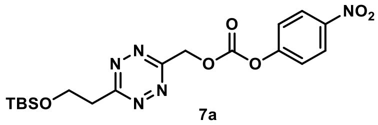

To a solution of **2** (760 mg, 2.82 mmol) and DIPEA (980 µL, 5.63 mmol) in 30 mL anhydrous DCM was added 4-nitrophenyl carbonochloridate (738 mg, 3.66 mmol) at 0 °C under argon atmosphere. The reaction mixture was monitored by TLC and stirred at room temperature for approximately 2 h, then diluted with DCM (30 mL). The resulting mixture was washed with water (60 mL × 3) and dried over anhydrous Na_2SO_4, and concentrated under vacuum. The resulting residue was purified using silica gel column chromatography to afford the desired product **7a** as a red oil (1.1 g, 90%).**^{1}H NMR** (400 MHz, DMSO-*d*$_6$) δ 8.42–8.34 (m, 2H), 7.68–7.62 (m, 2H), 5.97 (s, 2H), 4.25 (t, *J* = 6.2 Hz, 2H), 3.56 (t, *J* = 6.2 Hz, 2H), 0.78 (s, 9H), -0.00 (s, 6H). **^{13}C NMR** (101 MHz, DMSO-*d*$_6$) δ 169.78, 164.82, 155.51, 152.33, 145.82, 125.99, 122.92, 67.37, 61.20, 38.33, 26.02, 18.21, -5.10. **HRMS** [*M*+H]$^+$ *m/z* calcd. for [$C_{18}H_{26}N_5O_6Si$]$^+$ 436.1647, found 436.1645.

2.4.2 Synthesis of ***7b***

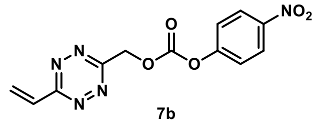

To a solution of **7a** (890 mg, 2.05 mmol) in THF (64 mL) and methanol (16 mL) was added 0.2 M HCl aqueous (100 mL). The mixture was further stirred at room temperature, and the reaction progress was monitored by TLC. After the reaction was completed, the mixture was diluted with 30 mL DCM, $NaHCO_3$ aqueous solution, and 20 mL water was added. The organic phase was separated, and water phase was further extracted with DCM (100 mL × 3). The combined organic phase was washed with brine, dried over Na_2SO_4, and concentrated under vacuum. The resulting residue was purified using silica gel column chromatography to afford the tetrazine intermediate as a red oil (480 mg).

To a solution of intermediate (480 mg, 1.49 mmol) and DIPEA (394 µL, 2.24 mmol) in anhydrous 20 mL DCM, MsCl (182 µL, 2.24 mmol) was added dropwise at 0 °C under an argon atmosphere. The mixture was stirred at room temperature, and the reaction progress was monitored by TLC. After the complete conversion to the methanesulfonate intermediate, DIPEA (394 µL, 2.24 mmol) was added, the mixture was further stirred at room temperature, and the reaction progress was monitored by TLC. After the reaction was completed, H_2O (20 mL) was then added, and the mixture was extracted with DCM (40 mL × 3). The combined organic phases were washed with brine, dried over Na_2SO_4, and concentrated under vacuum. The resulting residue was purified using silica gel column chromatography to afford the desired product **7b** as a red solid (320 mg, 51% over two steps). **^{1}H NMR** (400 MHz, DMSO-*d*$_6$) δ 8.38–8.30 (m, 2H), 7.65–7.58 (m, 2H), 7.25 (dd, *J* = 17.6, 10.9 Hz, 1H), 6.94 (dd, *J* = 17.5, 1.2 Hz, 1H), 6.19 (dd, *J* = 10.9, 1.1 Hz, 1H), 5.90 (s, 2H). **^{13}C NMR** (101 MHz, DMSO-*d*$_6$) δ 164.79, 164.36, 155.53, 152.38, 145.83, 130.87, 129.08, 126.01, 122.97, 67.41. **HRMS** [*M*+H]$^+$ *m/z* calcd. for [$C_{12}H_{10}N_5O_5$]$^+$ 304.0676, found 304.0674.

*2.4.3 Synthesis of **7***

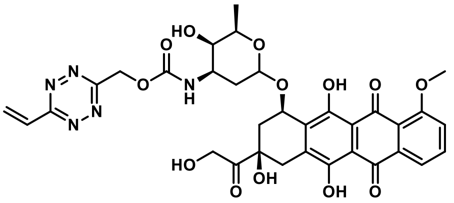

7

To a solution of **7b** (34 mg, 0.11 mmol) and DIPEA (115 µL, 0.66 mmol) in 10 mL anhydrous DCM, Dox·HCl (195 mg, 0.33 mmol) was added. The mixture was stirred at room temperature, and the reaction progress was monitored by TLC. After the reaction was completed, H_2O (20 mL) was then added, and the mixture was extracted with DCM (20 mL × 3). The combined organic phases were washed with brine, dried over Na_2SO_4, and concentrated under vacuum. The resulting residue was purified using silica gel column chromatography to afford the desired product **7** as a red oil (45.8 mg, 58%). **^{1}H NMR** (400 MHz, DMSO-d_6) δ 14.04 (s, 1H), 13.28 (s, 1H), 7.92 (d, *J* = 4.8 Hz, 2H), 7.67 (q, *J* = 4.9, 4.4 Hz, 1H), 7.27 – 7.13 (m, 2H), 6.85 (dd, *J* = 17.5, 1.2 Hz, 1H), 6.10 (dd, *J* = 11.0, 1.2 Hz, 1H), 5.56 – 5.46 (m, 2H), 5.43 (s, 1H), 5.23 (d, *J* = 3.6 Hz, 1H), 4.95 (s, 1H), 4.84 (t, *J* = 6.0 Hz, 1H), 4.76 (d, *J* = 5.7 Hz, 1H), 4.56 (d, *J* = 6.0 Hz, 2H), 4.14 (q, *J* = 6.5 Hz, 1H), 4.00 (s, 3H), 3.72 (m, 1H), 3.46 (d, *J* = 5.3 Hz, 1H), 2.97 (d, *J* = 3.3 Hz, 2H), 2.21 – 2.11 (m, 2H), 2.00 (q, *J* = 7.3 Hz, 1H), 1.87 (dd, *J* = 12.8, 3.8 Hz, 1H), 1.12 (d, *J* = 6.4 Hz, 3H). **^{13}C NMR** (101 MHz, DMSO-d_6) δ 213.90, 186.22, 186.09, 165.64, 164.08, 160.65, 156.05, 154.78, 154.46, 136.05, 135.31, 134.39, 133.93, 130.35, 128.04, 119.73, 119.51, 118.84, 110.57, 110.43, 100.30, 74.88, 69.74, 67.95, 66.66, 63.75, 62.95, 56.48, 47.47, 36.37, 31.98, 29.77, 17.01.
HRMS [*M*+Na]$^+$ *m/z* calcd. for $[C_{33}H_{33}N_5NaO_{13}]^+$ 730.1967, found 730.1961.

*2.5 Synthesis of **TCO-P$_4$***

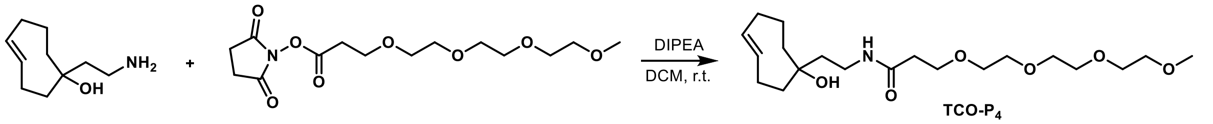

To a solution of 2,5-dioxopyrrolidin-1-yl 2,5,8,11-tetraoxatetradecan-14-oate (230 mg, 0.69 mmol) and DIPEA (143 µL, 0.83 mmol) in 10 mL anhydrous DCM, (*E*)-1-(2-aminoethyl)cyclooct-4-en-1-ol[1] (140 mg, 0.83 mmol) was added. The mixture was stirred at room temperature, and the reaction progress was monitored by TLC. After the reaction was completed, H_2O (20 mL) was then added, and the mixture was extracted with DCM (20 mL × 3). The combined organic phases were washed with brine, dried over Na_2SO_4, and concentrated under vacuum. The resulting residue was purified using silica gel column chromatography to afford the desired product **TCO-P$_4$** as a white oil (242 mg, 90%). **^{1}H NMR** (400 MHz, Benzene-d_6) δ 6.60–6.40 (m, 1H), 5.77 (ddd, *J* = 15.1, 10.9, 3.8 Hz, 1H), 5.50 (ddd, *J* = 15.5, 11.3, 3.3 Hz, 1H), 3.50–3.25 (m, 16H), 3.12 (s, 3H), 2.61–2.45 (m, 2H), 2.31–2.20 (m, 3H), 2.04 (m, 1H), 1.91–1.81 (m, 2H), 1.81–1.71 (m, 1H), 1.69–1.64 (m, 1H), 1.52 (m, 2H), 1.43 (m, 1H), 1.21 (m, 1H). **^{13}C NMR** (101 MHz, Benzene-d_6) δ 170.74, 134.09, 132.50, 71.98, 71.57, 70.60, 70.43, 70.41, 70.23, 70.03, 67.15, 58.31, 47.83, 47.72, 38.99, 37.08, 35.23, 34.33, 30.52, 27.32. **HRMS** [*M*+H]$^+$ *m/z* calcd. for $[C_{20}H_{38}NO_6]^+$ 388.2694, found 388.2685.

3. Evaluation and optimization of the labeling reaction conditions between 1 and Cys

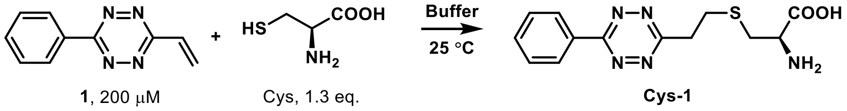

HPLC conditions: Poroshell 120, EC-C_{18} column: 3.0 × 150 mm, 2.7 µm; column temperature: 35 °C; gradient: 0 min, 20% B; 0–8 min, 20–100% B; flow rate: 0.6 mL/min.
The yields were calculated as follow: Product yield (%) = $I_p/I_0 \times 100$. I_p represents the peak areas of the ligation product at the indicated time. I_0 represents the peak areas of **1** at 0 min. The peaks were monitored by HPLC at 520 nm.

4. Determine labeling reaction kinetics between voTz 1 and Cys

The second-order rate constant for the labeling reactions between tetrazine **1** and Cys was determined at 25 °C in PB (30% MeCN, 20 mM, pH 8.0) under second-order conditions using a 6000+ (Quawell) UV-Vis spectrophotometer. The concentrations of cysteine and tetrazine were identical. The specific difference in absorption wavelength (300 nm) between **1** and Cys-1 was measured over time. The second-order rate constant k_2 was calculated from the slope of a plot of $(1/c - 1/c_0)$ versus time.

5. Competitive labeling reaction with other nucleophilic amino acids

For the competitive labeling experiment, 10 equiv. (2 mM) of *N*-Fmoc-Lys, *N*-Fmoc-Arg, Ser, Tyr, and 1.3 equiv. (260 μM) of Cys were added in PB before the addition of **1** (200 μM). The resulting solution was maintained at 25 °C and monitored by LC–MS. HPLC conditions: Poroshell 120, EC-C_{18} column: 3.0 × 150 mm, 2.7 μm; column temperature: 35 °C; gradient: 0 min, 5% B; 0–8 min, 5–100% B; flow rate: 0.5 mL/min.

6. Chemoselective peptide labeling

HPLC conditions: Poroshell 120, EC-C_{18} column: 3.0 × 150 mm, 2.7 μm; column temperature: 35 °C; gradient: 0 min, 5% B; 0–8 min, 5–100% B; flow rate: 0.5 mL/min.
Each voTz analogue (1.1 equiv.) was added to a 0.25 mL vial containing solvent. The resulting solution was pipetted up and down five times, followed by the addition of a peptide solution (P_1–P_9) to a final concentration of either 20 μM or 2.5 mM in 200 μL. The solution was further pipetted up and down ten times and allowed to sit at room temperature. The chromatogram peak areas of all relevant Cys-containing species were integrated, and the yield was determined using the following equation: Yield (%) = $I_{\text{product}}/(I_{\text{starting}} + I_{\text{product}} + I_{oxidation} + I_{\text{side product}}) \times 100$, where I_{starting}, I_{product}, $I_{oxidation}$, and $I_{\text{side product}}$ respectively represent the average ion counts of remaining starting material, product, oxidized starting material and any side product, if present[2].

7. Nano-structure characterization of peptide conjugates

Preparation of samples: H_2O was added to the stock solution of peptide conjugates (2.5 mM in DMSO) to obtain a 50 μM solution (H_2O, containing 2% DMSO) which was then sonicated in a bath sonicator (120 W) for 10 min.

7.1 Tyndall effect analyses

For the Tyndall effect assay, the solutions of peptide conjugates were illuminated using a red laser pointer pen (635 nm, 1 mW).

7.2 Dynamic light scattering (DLS) measurements

DLS analyses and particle zeta potential values were determined using a Nano ZS ZEN 3690 instrument (Malvern Instruments).

7.3 TEM analyses

Transmission electron microscope (TEM) images were obtained using a Talos FEI 200 instrument (Thermo Scientific) with the electron acceleration voltage was 80 kV. Before analysis, the solutions of peptide conjugates were aged at room temperature for 4 h. Thirty microliters of peptide solutions were placed on parafilm, and absorbed onto a copper mesh covered with a carbon support film for 2 min. Excess stain was removed using filter paper and then allowed to dry at room temperature.

8. The critical micelle concentration of peptide conjugates

A mixture of Nile Red (final concentration, 1.0 μM) and peptide conjugates (serially diluted into different concentrations) in PBS（5% DMSO）were shaken at room temperature overnight. The fluorescence emission intensity of Nile Red at 525 nm (λ_{ex} = 485 nm) was measured with a FluoroMax-4 fluorometer (Horiba, Kyoto, Japan).

9. Kinetic studies of bioorthogonal reactions

The second-order rate constants of the bioorthogonal reactions between peptide conjugates (final concentration: 50 μM) and *trans*-cyclooctene (TCO, final concentration: 50 μM) in buffer at 37 °C were determined by UV–Vis spectroscopy. The absorption was measured at indicated time intervals, and the concentration of tetrazine was calculated based on the change in its absorption peak intensity at 520 nm over time. The k_2 value was then determined from the slope of the (1/c – 1/c_0) against time, where c_0 is the concentration of tetrazine analogue at time zero and c is the corresponding concentration at the indicated time. Each experiment was performed in triplicate, and data were analyzed using Origin Pro 2015.

10. *In vivo* release evaluation of the RGD-FL

10.1 Time lapse imaging of bioorthogonal reaction triggered RGD-FL releasing in live SKOV3 cells

SKOV3 cells were seeded onto 12-well cell culture clusters and cultured for 48 h. Subsequently, the cells were stained with Hoechst 33342 (250 nM) for 5 min to label the nuclei, washed briefly, then pre-treated with RGD-FL (5 μM) for 1 h at 37 ºC. After washing with PBS, TCO (50 μM) was added to the cells, which were then imaged using a confocal laser scanning microscope. Images were analyzed with ZEN blue software to measure the intensity (λ_{ex} = 488 nm, λ_{em} = 500−600 nm). Three times each imaging experiment was repeated independently with similar results.

10.2 Fluorescence imaging of SKOV3 cells

SKOV3 cells were seeded onto 35-mm glass-bottom dishes and cultured for 48 h. Subsequently, the cells were stained with Hoechst 33342 (250 nM) for 5 min to label the nuclei, washed briefly, and treated with RGD-FL (5 μM) at 37 °C for 60 min. After washing with PBS, the cells were treated with TCO (50 μM) for 60 min, the cells were imaged in 1X Cell Imaging Solution (Invitrogen) using a confocal laser scanning microscope (Zeiss 880, 63x magnification). SKOV3 cells treated only with RGD-FL were used as a control.

10.3 Targeting study of RGD-FL in live cells

SKOV3 cells ($\alpha_v\beta_3$ positive) or MCF-7 cells ($\alpha_v\beta_3$ negative) were seeded onto 12-well cell culture clusters and cultured for 48 h. Subsequently, the cells were stained with Hoechst 33342 (250 nM) for 5 min to label the nuclei, washed briefly, and treated with RGD-FL (5 μM) at 37 °C for 60 min. After washing with PBS, the cells were treated with TCO (50 μM) for 60 min. SKOV3 cells treated only with RGD-FL were used as a control. Blocking studies were performed by pre-treated of SKOV3 cells with a 20-fold excess of unmodified RGD for 1 h before incubation with RGD-FL. The cells were imaged in 1X Cell Imaging Solution (Invitrogen) using a confocal laser scanning microscope (Zeiss 880).

10.4 Time lapse imaging of bioorthogonal reaction triggered 18-4-FL releasing in live MDA-MB-231 cells

MDA-MB-231 cells were seeded onto 12-well cell culture clusters and cultured for 48 h. Cells were pre-treated with 18-4-FL (5 μM) for 1 h at 37 ºC. After washing with PBS, TCO (50 μM) was added to the cells, which were then imaged at specific times using a confocal laser scanning microscope.

11. Cell viability assays

11.1 Analysis of cellular uptake of RGD-Dox over various time intervals using flow cytometry

B16F10 cells were seeded onto 6-well cell culture clusters and incubated for 48 hours. After the incubation period, the cells were exposed to RGD-Dox (10 μM) at 37 °C for different durations. Subsequently, the cells were trypsinized and underwent three rinses with PBS. Flow cytometry analysis (Fortessa, BD, USA) was utilized to evaluate the intrinsic fluorescence intensity of Dox with an excitation/emission wavelength of 488/610 nm.

11.2 Cytotoxicity of drugs, PDCs and activable-Drugs

All cells were seeded onto a 96-well plate at a density of 10^5 cells/well. After 24 hours of incubation at 37 °C with 5% CO_2, the cells were treated with tested compounds, including free Drugs, PDCs, or activable-Drugs (cells were pre-incubated with different concentrations of PDCs for 2 h followed by incubation with 200 μM TCO), at varied concentrations (150 μL final volume per well) for 48 hours. Then, 10 μL of CCK8 solution was added to each well, and

the incubation continued for another 1−2 hours. Following this, the absorbance at 480 nm was measured using a plate reader. The cell viability of the solvent control group was set at 100%, and the cytotoxicity of the compounds was determined by comparing the absorbance to that of the solvent control group.

11.3 Cytotoxicity of TCO

All cells were seeded onto a 96-well plate at a density of 10^5 cells/well. After 24 hours of incubation at 37 °C with 5% CO_2, the cells were treated with TCO (200 μM, 150 μL final volume per well) for 48 hours. Then, 10 μL of CCK8 solution was added to each well, and the incubation continued for another 1−2 hours. Following this, the absorbance at 480 nm was measured using a plate reader. The cell viability of the solvent control group was set at 100%, and the cytotoxicity of the compounds was determined by comparing the absorbance to that of the solvent control group.

11.4 Western blotting analysis

Rabbit anti-p53 antibody (ab131442), Rabbit anti-Bax antibody (ab32503), Rabbit anti-Caspase-3 antibody (ab184787), Rabbit anti-β-actin antibody (ab8227), Rabbit anti-GAPDH antibody (ab181602), and Goat anti-Rabbit IgG HL (HRP, ab205718) were obtained from Abcam Ltd (Cambridge, UK).
B16F10 cells were cultured for 48 h. In the treatment group, RGD-Dox (5 μM) and TCO (50 μM, 10 eq.) were administered at 2 h intervals to activate Dox. For comparison, the remaining four control groups received RGD-Dox (5 μM), TCO (50 μM), Dox (5 μM), or PBS for 24 h. Samples were lysed in RIPA solution. Protein content was quantified using the BCA method (Thermo Fisher). Proteins were loaded onto a 12.5% SDS-polyacrylamide gel and blotted onto a polyvinylidene fluoride (PVDF) membrane (Millipore). The blotted PVDF membranes were blocked with 5% milk in Tris-buffered saline with Tween 20 and incubated overnight at 4 °C with the primary antibody. Proteins were finally detected with a horseradish peroxidase (HRP)-conjugated secondary antibody. β-actin and GAPDH were used as internal standards.

12. Cell uptake analysis

12.1 Calculation of the fluorescence coefficient (F_c)

The RGD-Dox or Dox was dissolved in PBS (10% DMSO) to obtain a 10 μM solution. The F_c was the radio calculated from the peak emission intensity (Int.) at 595 nm (λ_{ex} = 480 nm) of Dox and RGD-Dox after subtracting background noise.

12.2 Uptake calculation of the B16F10 cells

Images were analyzed with ZEN blue software to measure the intensity (λ_{em} = 560−650 nm) of individual cells. A lognormal distribution was identified as the best-fit curve from a histogram of the mean intensity of individual cells. The mean fluorescence intensity for each treatment group (n = 3) was calculated based on n = 50 cells. The mean and standard deviation were analyzed using Origin Pro 2015.

12.3 Comparison of cell uptake between RGD-Dox and Dox using flow cytometry

The cellular uptake of RGD-Dox and Dox was analyzed using flow cytometry. B16F10 cells, characterized by $\alpha_v\beta_3$ positivity, were seeded onto 6-well cell culture clusters and incubated for 48 hours. Following the incubation period, the cells were exposed to either RGD-Dox (10 μM), Dox (10 μM), or PBS at 37 °C for 2 hours. Subsequently, the cells were trypsinized and underwent three PBS rinses. Flow cytometry analysis (Fortessa, BD, USA) was utilized to evaluate the intrinsic fluorescence intensity of Dox with an excitation/emission wavelength of 488/610 nm.

12.4 Cell uptake analysis by fluorescence imaging

B16F10 cells ($\alpha_v\beta_3$ positive) or LX2 cells ($\alpha_v\beta_3$ negative) were seeded onto 12-well cell culture clusters and cultured for 48 h. Subsequently, the cells were stained with Hoechst 33342 (250 nM) for 5 min to label the nuclei, washed briefly, and treated with RGD-Dox or Dox (10 μM) at 37 °C for 2 h, then washed with PBS for three times. Blocking studies were performed by pre-treated of B16F10 cells with a 50-fold excess of unmodified RGD for 2 h before incubation with RGD-Dox. Cells were imaged in 1X Cell Imaging Solution (Invitrogen) using a confocal laser scanning microscope (Zeiss 880).

13. *In vivo* anticancer efficacy

13.1 The accumulation of RGD-Dox at various time points in tumor tissues

When the tumor volume reached approximately 400 mm^3, five mice in each group received an i.v. injection of 3.67 $\mu mol \cdot kg^{-1}$ RGD-Dox. After various time intervals, the mice were sacrificed, and tumor tissues were dissected. About 0.5 to 1 g of tissue was ground into a homogenate, and proteins were precipitated by the adding two volumes of acetonitrile (1 mL). After centrifugation, the supernatant was removed, concentrated to 100 µL, and 20 µL was used for HPLC–MS analysis. The RGD-Dox concentration in the sample was determined from a standard curve of the peak area of the characteristic absorption of RGD-Dox at 520 nm.

13.2 Calculation of the tumor growth inhibition values (TGI)

Tumor growth inhibition values (TGI) were calculated using the following formula: TGI (%) = $(1 - RTV_{treatment}/RTV_{control})*100$. RTV refers to relative tumor volume, representing the ratio of the volume at the end of treatment to the volume at the beginning of treatment.

Supplementary Tables

Supplementary Table 1. **Reaction yields between tetrazine 1 and Cys under different conditions**.

Entry	Buffer	Co-solvent	pH	Time	Yield%
1	PB	30% A	8.0	30 min	97
2	PB	30% A	8.0	1 h	99
3	PB	30% A	7.4	1 h	95
4	PB	30% A	6.5	1 h	64
5	PB	30% A	6.5	4.5 h	97
6	PB	10% A	8.0	1 h	98
7	PB	1% A	8.0	1 h	98
8	PB	30% B	8.0	1 h	99
9	Tris	30% A	8.0	1 h	97
10	HEPES	30% A	8.0	1 h	93
11	-	A	-	10 min	94%[a]

[a] The reaction was carried out under a 30 mM concentration of **1** with 1.0 equiv. of DIPEA in MeCN. Co-solvent A is MeCN and B is DMSO.

Supplementary Table 2. **The 48 h cytotoxicity of Dox, RGD-Dox and activable-Dox, against corresponding cells**.

Treatment	IC_{50}[a]			
	B16F10	U87	SKOV3	LX2
**Dox**	2.05 (1.64–2.54)	3.26 (2.75–3.85)	4.45 (4.06–4.89)	3.37 (2.86-3.95)
**RGD-Dox**	24.03 (20.42–28.87)	29.18 (26.12–32.60)	55.52 (50.35–61.39)	56.73 (49.74–65.59)
**Activable-Dox**	0.16 (0.12–0.21)	0.95 (0.77–1.17)	1.08 (0.97–1.21)	16.42 (14.35–18.82)

[a] 95% confidence interval is given in parentheses.

Supplementary Table 3. **The 48 h cytotoxicity of other Drugs, prodrug PDCs, and activable-Drugs against corresponding cells**.

Treatment	IC_{50}[a]			
	BP9a-Dox HepG2	18-4-Dox MDA-MB-231	RGD-SN-38 U87	RGD-SN-38 SKOV3
**Drug**	5.89 (4.72–7.30)	4.41 (3.75–5.17)	1.71 (1.30–2.24)	2.71 (2.09–3.50)
**PDC**	42.41 (37.99–48.19)	20.79 (19.07–22.74)	5.96 (5.12–6.90)	10.28 (8.42–12.49)
**Activable-Drug**	1.78 (1.39–2.26)	2.19 (2.02–2.38)	0.39 (0.31–0.49)	1.14 (0.87–1.49)

[a] 95% confidence interval is given in parentheses.

Supplementary Figures

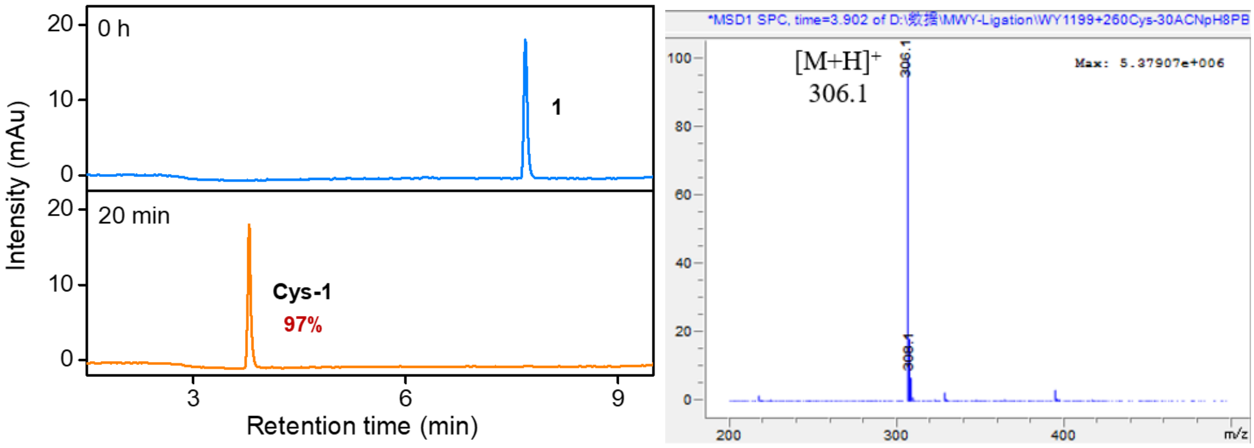

Supplementary Fig. 1. **Monitoring of the reaction between 1 and Cys**. HPLC–UV monitoring of the reaction between **1** and Cys in phosphate buffer (30% MeCN, 20 mM, pH 8.0) after 30 min at 520 nm and the associated mass spectrum of the reaction solution at 3.9 min.

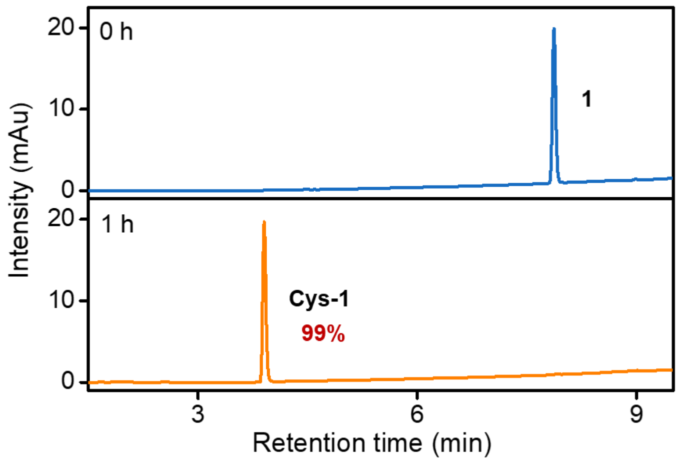

Supplementary Fig. 2. **Monitoring of the reaction between 1 and Cys**. HPLC–UV monitoring of the reaction between **1** and Cys in phosphate buffer (30% MeCN, 20 mM, pH 8.0) after 1 h at 520 nm.

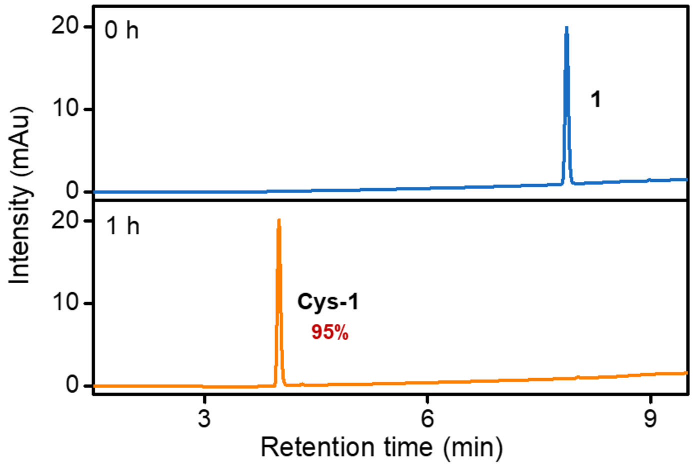

Supplementary Fig. 3. **Monitoring of the reaction between 1 and Cys**. HPLC–UV monitoring of the reaction between **1** and Cys in a phosphate buffer (30% MeCN, 20 mM, pH 7.4) after 1 hour at 520 nm.

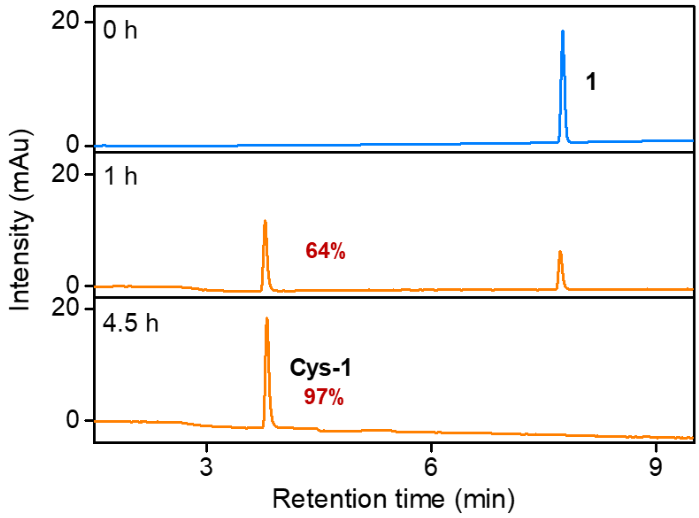

Supplementary Fig. 4. **Monitoring of the reaction between 1 and Cys**. HPLC–UV monitoring of the reaction between **1** and Cys in phosphate buffer (30% MeCN, 20 mM, pH 6.5) after 1 h and 4.5 h at 520 nm.

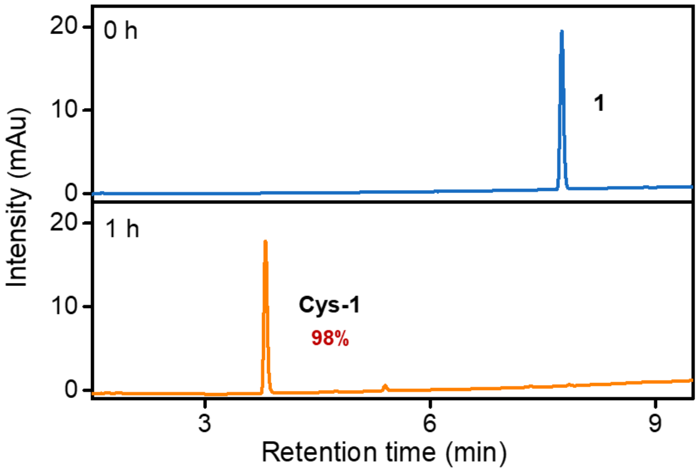

Supplementary Fig. 5. **Monitoring of the reaction between 1 and Cys**. HPLC–UV monitoring of the reaction between **1** and Cys in phosphate buffer (10% MeCN, 20 mM, pH 8.0) after 1 h at 520 nm.

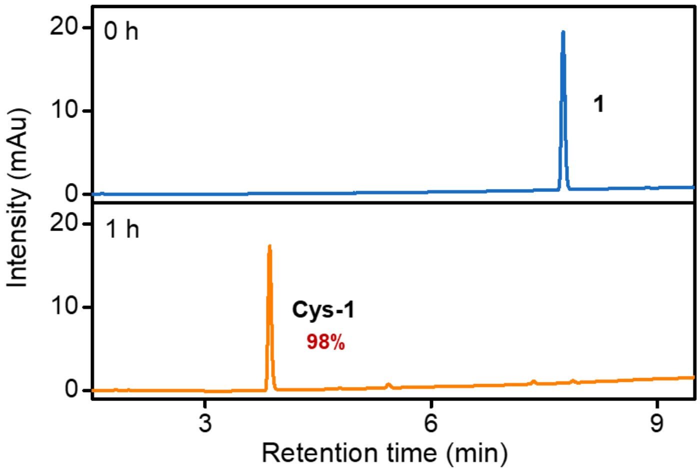

Supplementary Fig. 6. **Monitoring of the reaction between 1 and Cys**. HPLC–UV monitoring of the reaction between **1** and Cys in phosphate buffer (1% MeCN, 20 mM, pH 8.0) after 1 h at 520 nm.

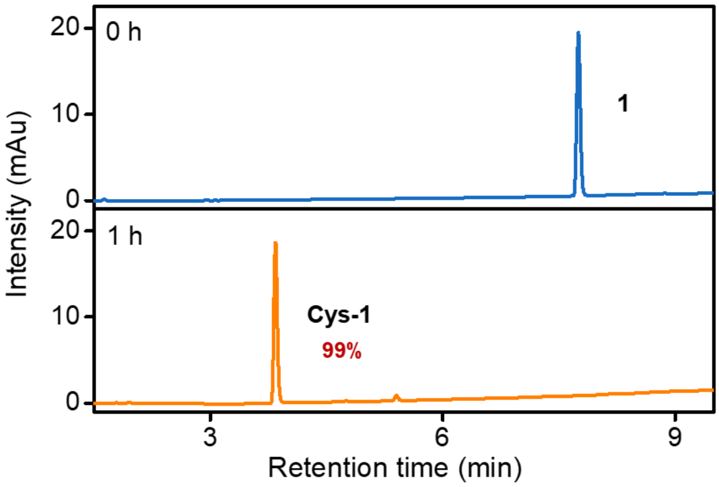

Supplementary Fig. 7. **Monitoring of the reaction between 1 and Cys**. HPLC–UV monitoring of the reaction between **1** and Cys in phosphate buffer (30% DMSO, 20 mM, pH 8.0) after 1 h at 520 nm.

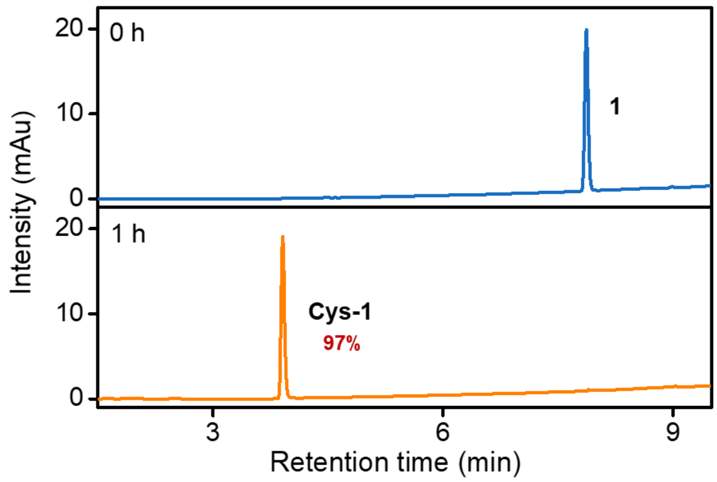

Supplementary Fig. 8. **Monitoring of the reaction between 1 and Cys**. HPLC–UV monitoring of the reaction between **1** and Cys in Tris (30% MeCN, 100 mM, pH 8.0) after 1 h at 520 nm.

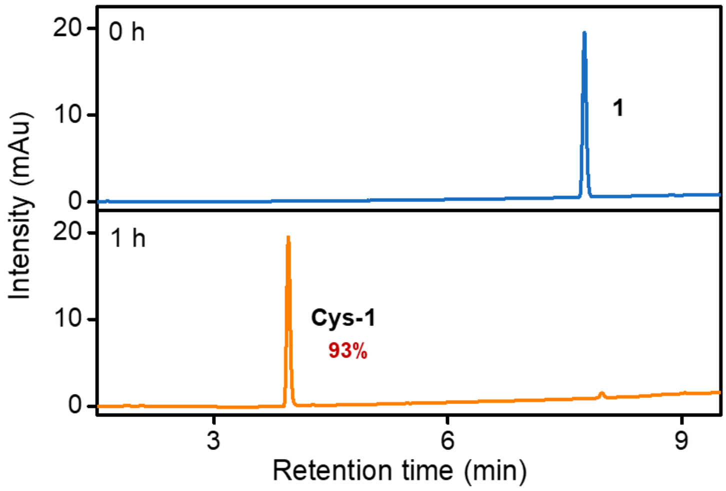

Supplementary Fig. 9. **Monitoring of the reaction between 1 and Cys**. HPLC–UV monitoring of the reaction between **1** and Cys in HEPES (30% MeCN, 100 mM, pH 8.0) after 1 h at 520 nm.

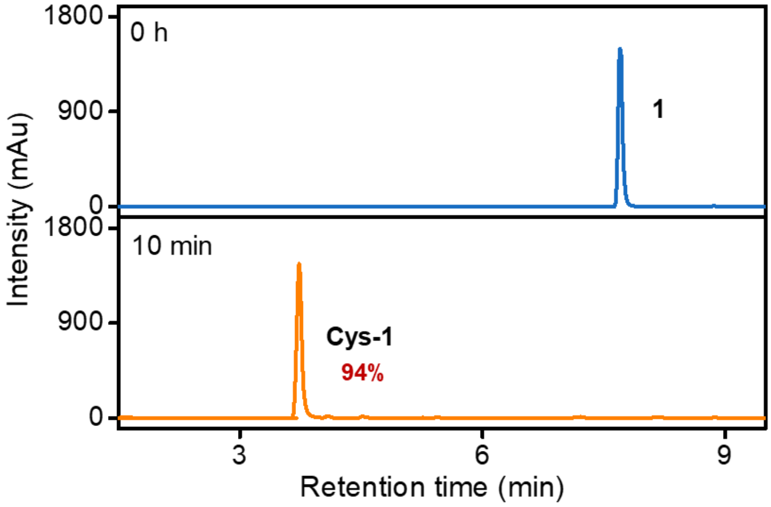

Supplementary Fig. 10. **Monitoring of the reaction between 1 and Cys**. HPLC–UV monitoring of the reaction between **1** and Cys in MeCN with 1.0 eq. DIPEA after 10 min at 520 nm.

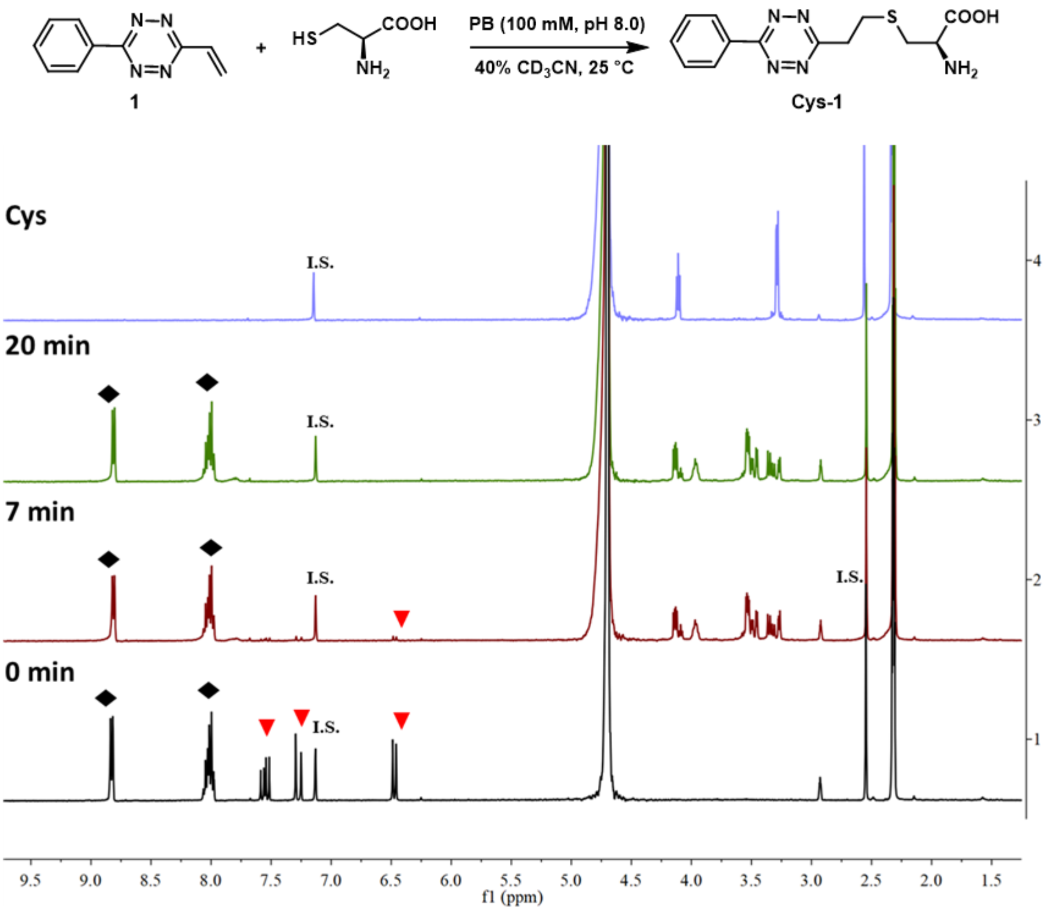

Supplementary Fig. 11. **Monitoring of the reaction between tetrazine 1 and Cys by ^{1}H NMR spectroscopy**. Legend: ◆: phenyl hydrogens; ▼: vinyl hydrogens; I.S.: internal standard (mesitylene). PB of NMR is phosphate buffer prepared with deuterium oxide.

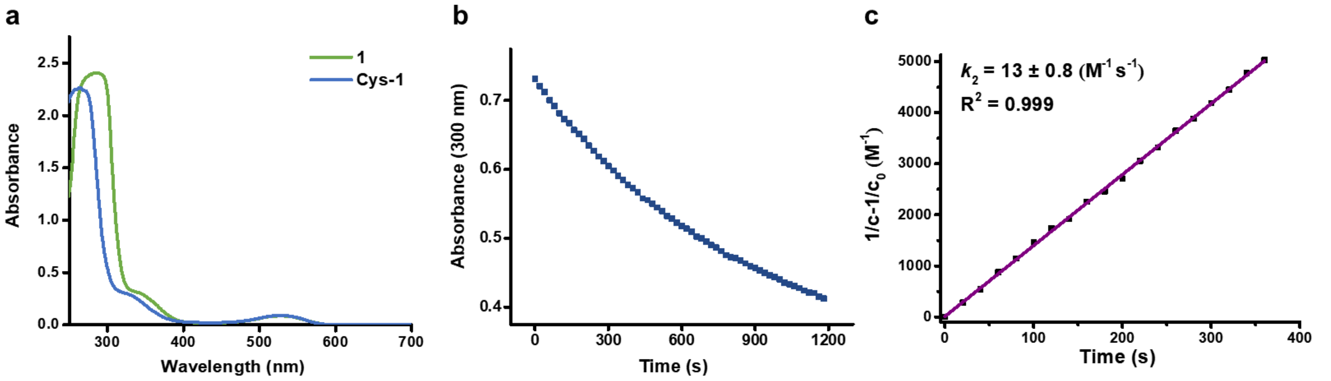

Supplementary Fig. 12. **Determine labeling reaction kinetics between voTz 1 and Cys**. (a) Absorption spectra of **1** (200 µM) and Cys-1 (200 µM) in PB (20 mM, pH = 8.0, 30% MeCN). The specific difference in absorption wavelength (300 nm) between **1** and Cys-**1** was used to measure reaction kinetics. (b) The absorption change over time of the reaction between **1** (50 µM) and Cys (50 µM) at 300 nm. (c) Calculation of the second-order rate constant of **1** (50 µM) and Cys (50 µM) by measuring the absorbance at 300 nm.

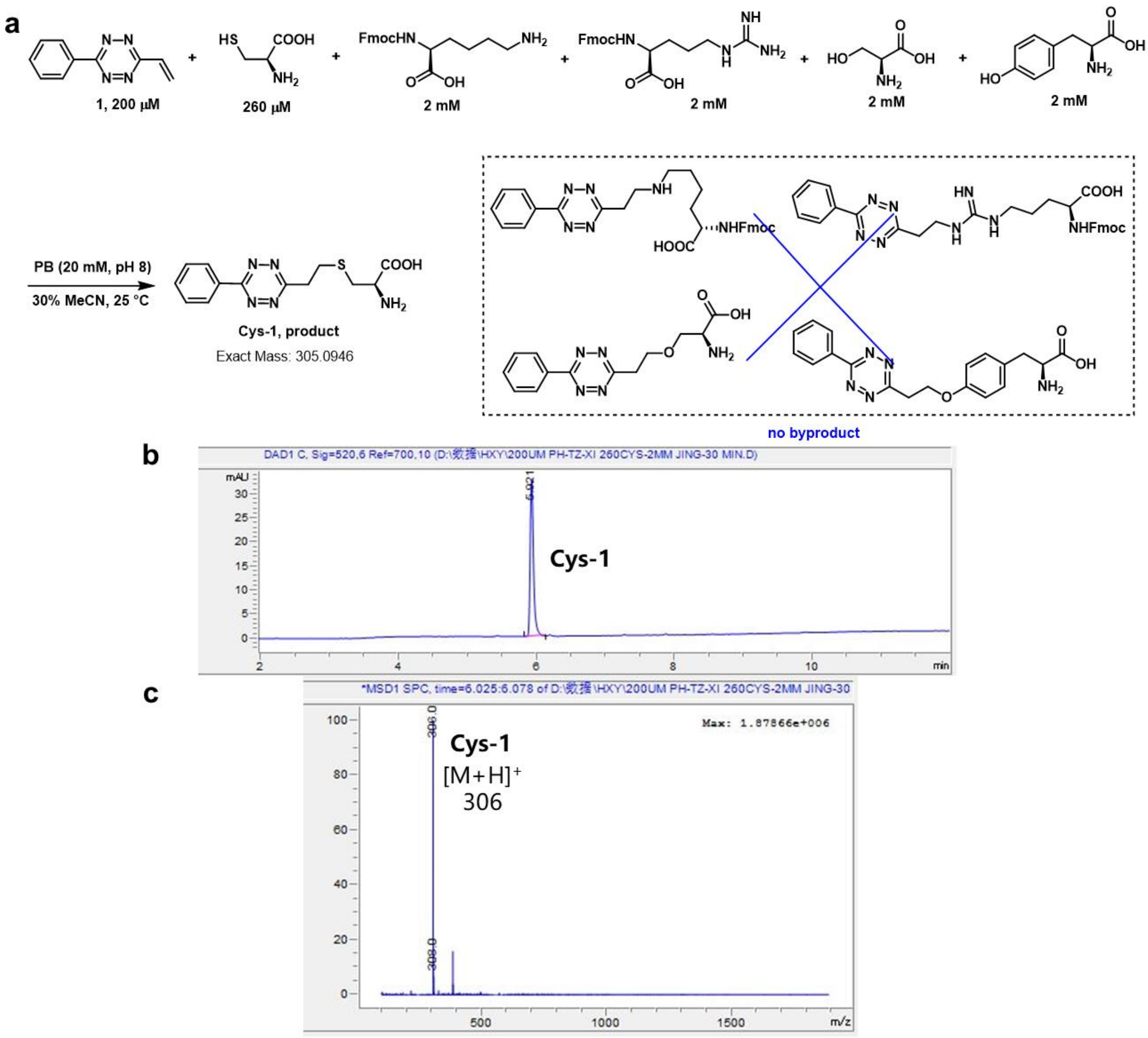

Supplementary Fig. 13. Competitive labeling reaction with other nucleophilic amino acids. (a) The reaction of voTz **1** with Cys in the presence of a competitive amount of *N*-Fmoc-Lys, *N*-Fmoc-Arg, Ser, and Tyr. (b) HPLC–UV chromatogram at 520 nm of the reaction. (c) The associated mass spectrum of the product Cys-**1**.

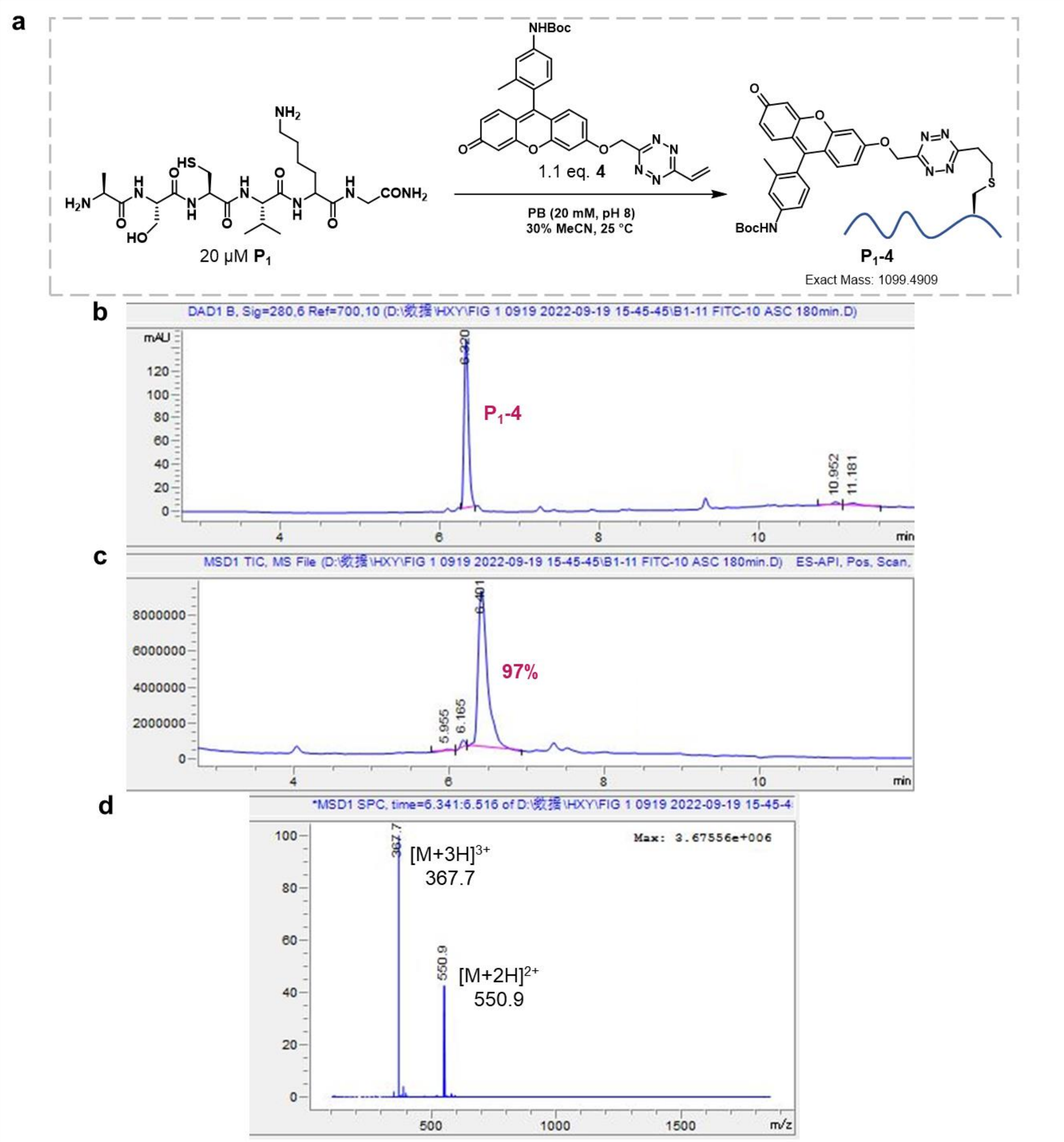

Supplementary Fig. 14. **Analysis the labeling reaction involving P**$_1$ **with voTz 4 using LC–MS**. (a) The labeling reaction involving P$_1$ and **4**. (b) HPLC–UV chromatogram at 280 nm of the reaction. (c) HPLC–MS chromatogram of the reaction. (d) The associated mass spectrum of the product P$_1$-4.

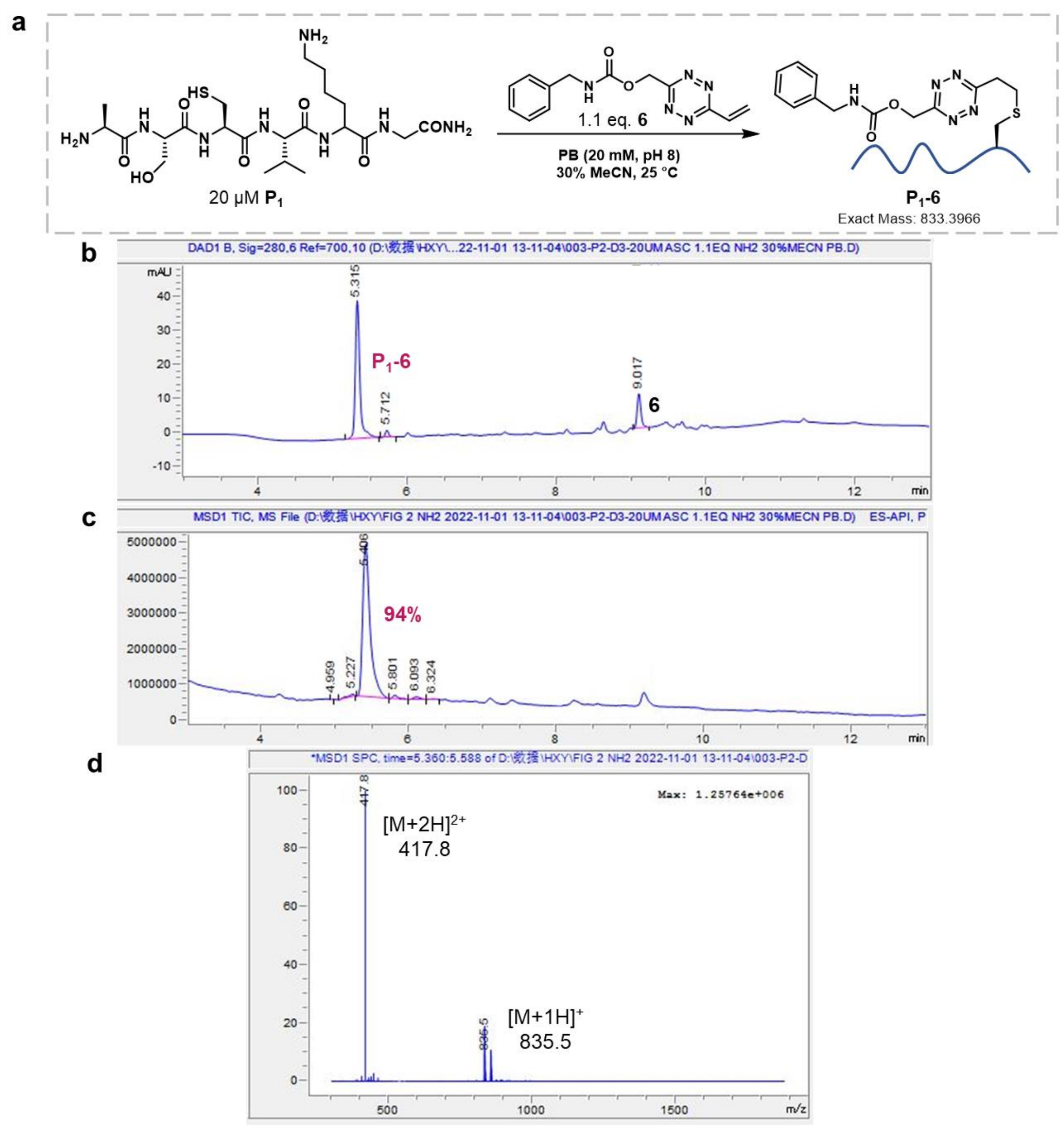

Supplementary Fig. 15. **Analysis the labeling reaction involving P_1 with voTz 6 using LC–MS**. (a) The labeling reaction involving P_1 and **6**. (b) HPLC–UV chromatogram at 280 nm of the reaction. (c) HPLC–MS chromatogram of the reaction. (d) The associated mass spectrum of the product P_1-6.

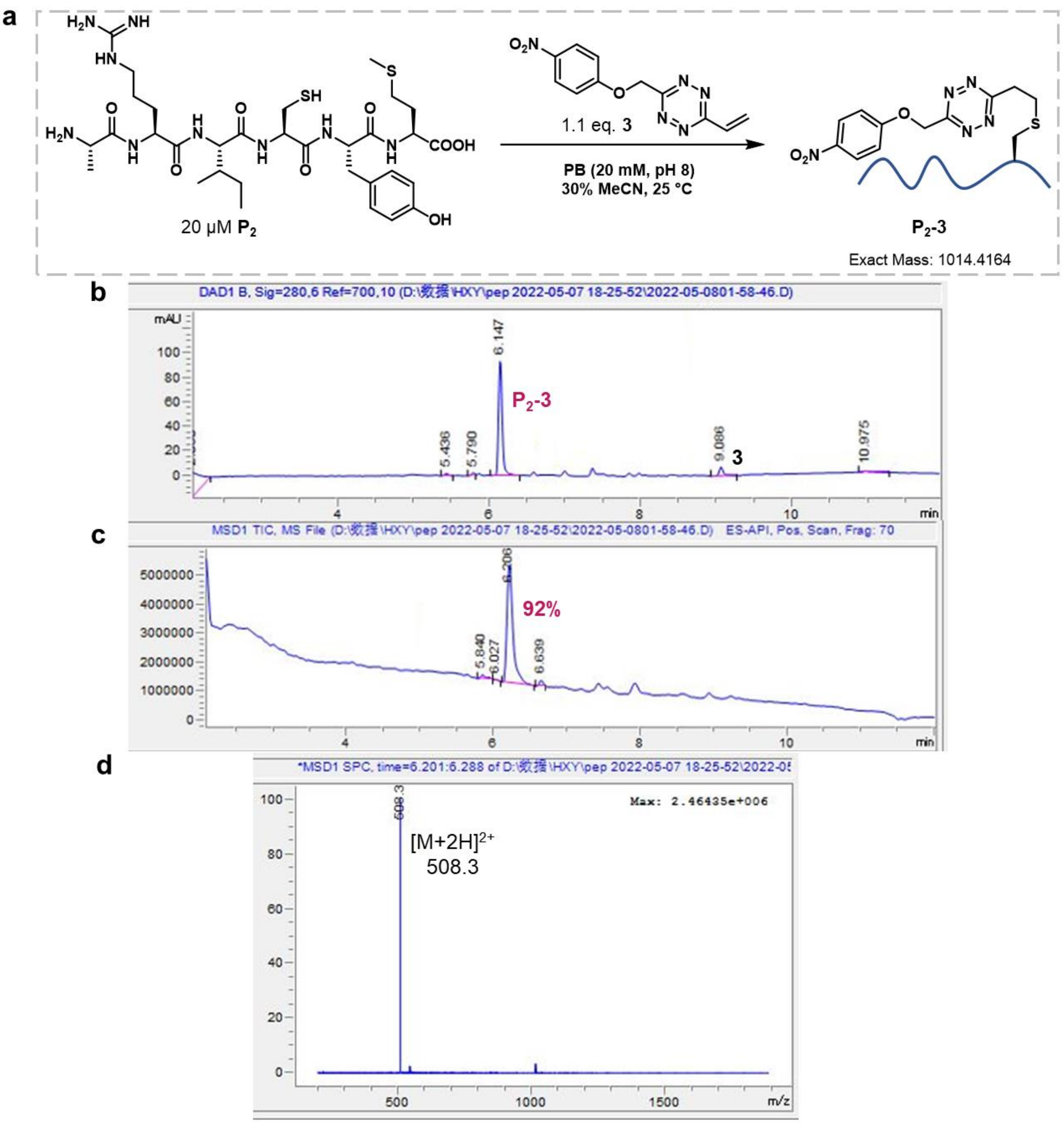

Supplementary Fig. 16. **Analysis the labeling reaction involving P$_2$ with voTz 3 using LC–MS**. (a) The labeling reaction involving P$_2$ and **3**. (b) HPLC–UV chromatogram at 280 nm of the reaction. (c) HPLC–MS chromatogram of the reaction. (d) The associated mass spectrum of the product P$_2$-3.

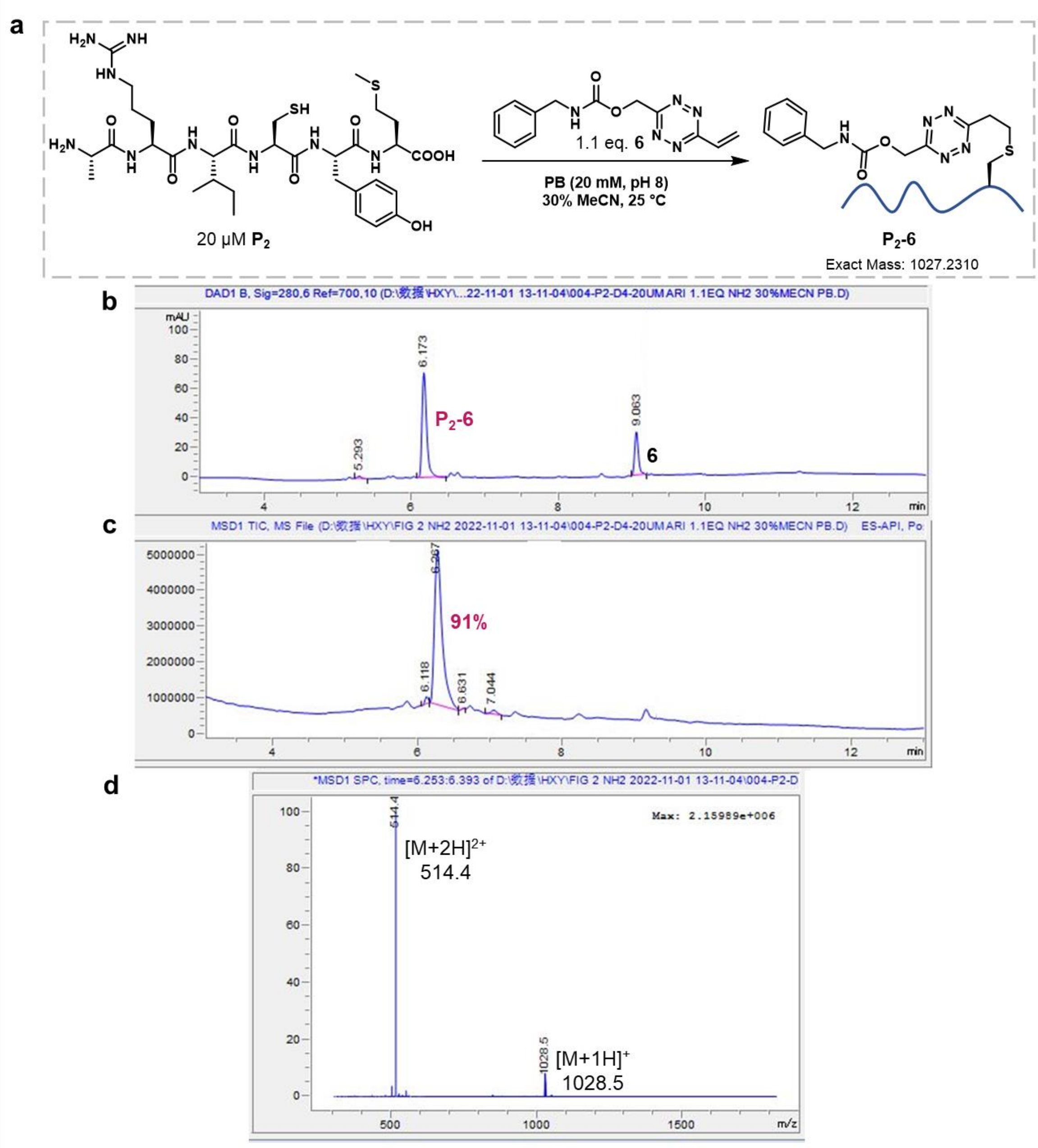

Supplementary Fig. 17. **Analysis the labeling reaction involving P_2 with voTz 6 using LC–MS**. (a) The labeling reaction involving P_2 and **6**. (b) HPLC–UV chromatogram at 280 nm of the reaction. (c) HPLC–MS chromatogram of the reaction. (d) The associated mass spectrum of the product P_2-6.

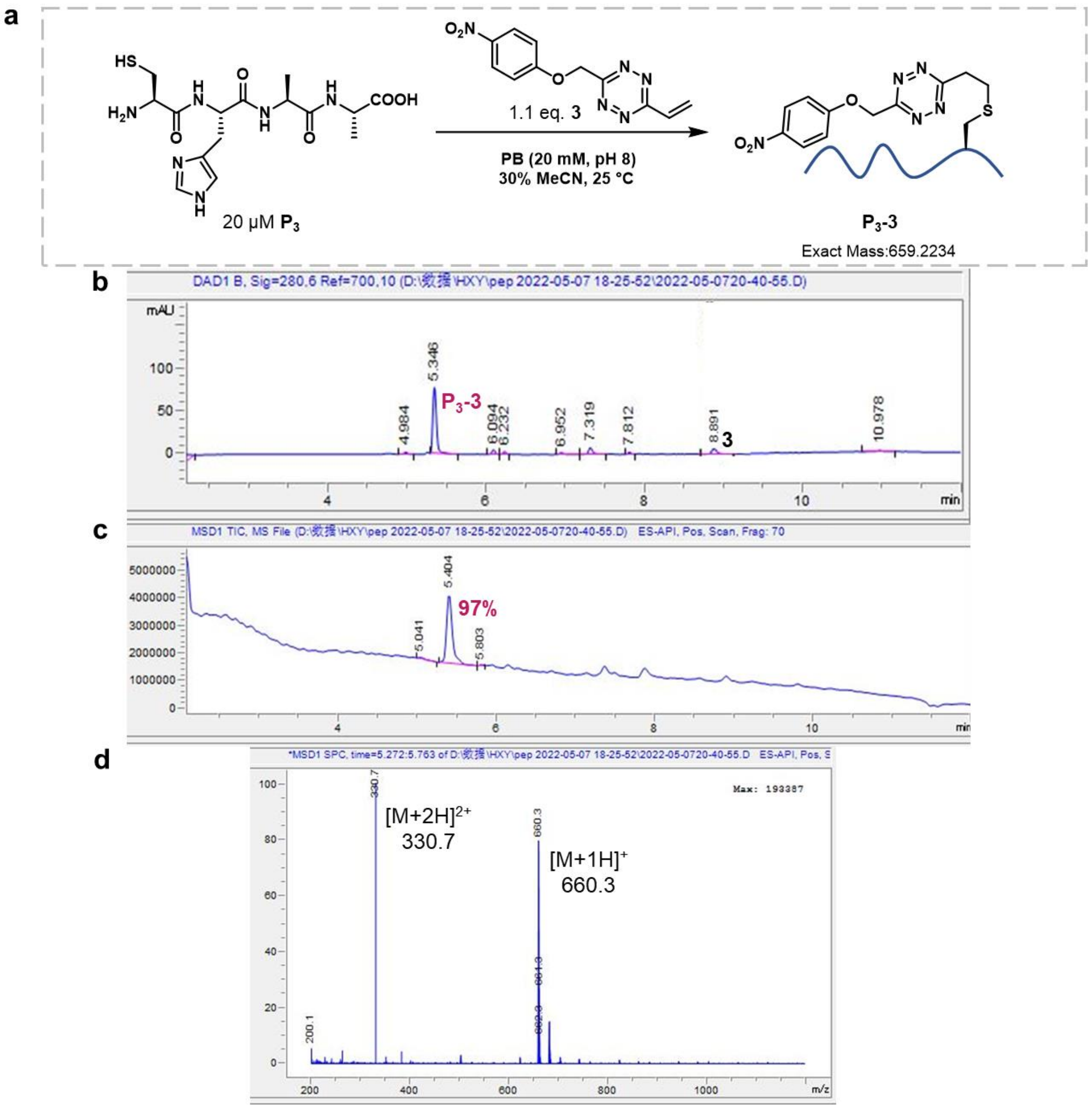

Supplementary Fig. 18. **Analysis the labeling reaction involving P_3 with voTz 3 using LC–MS**. (a) The labeling reaction involving P_3 and **3**. (b) HPLC–UV chromatogram at 280 nm of the reaction. (c) HPLC–MS chromatogram of the reaction. (d) The associated mass spectrum of the product P_3-3.

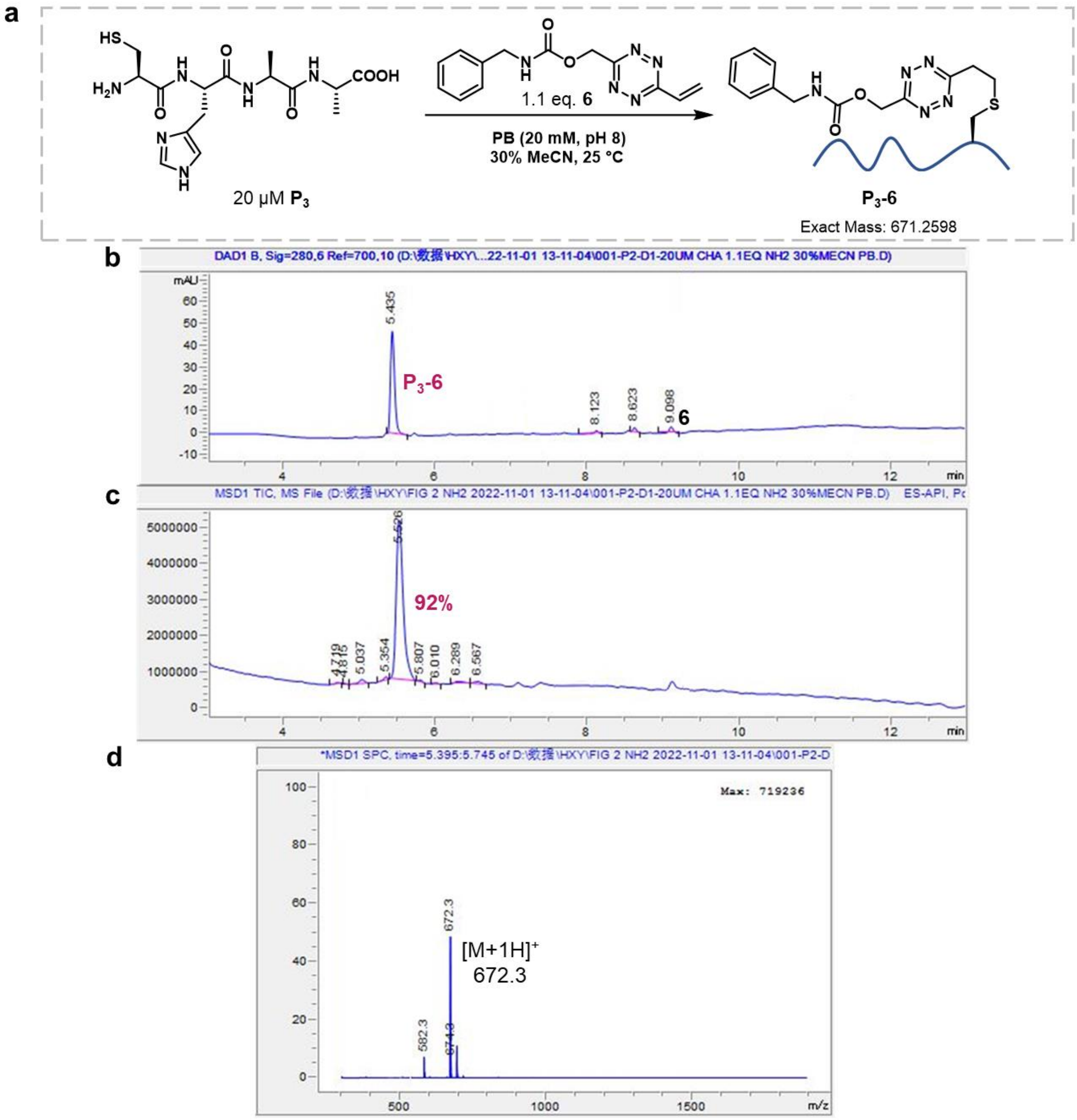

Supplementary Fig. 19. **Analysis the labeling reaction involving P₃ with voTz 6 using LC–MS**. (a) The labeling reaction involving P_3 and **6**. (b) HPLC–UV chromatogram at 280 nm of the reaction. (c) HPLC–MS chromatogram of the reaction. (d) The associated mass spectrum of the product P_3-6.

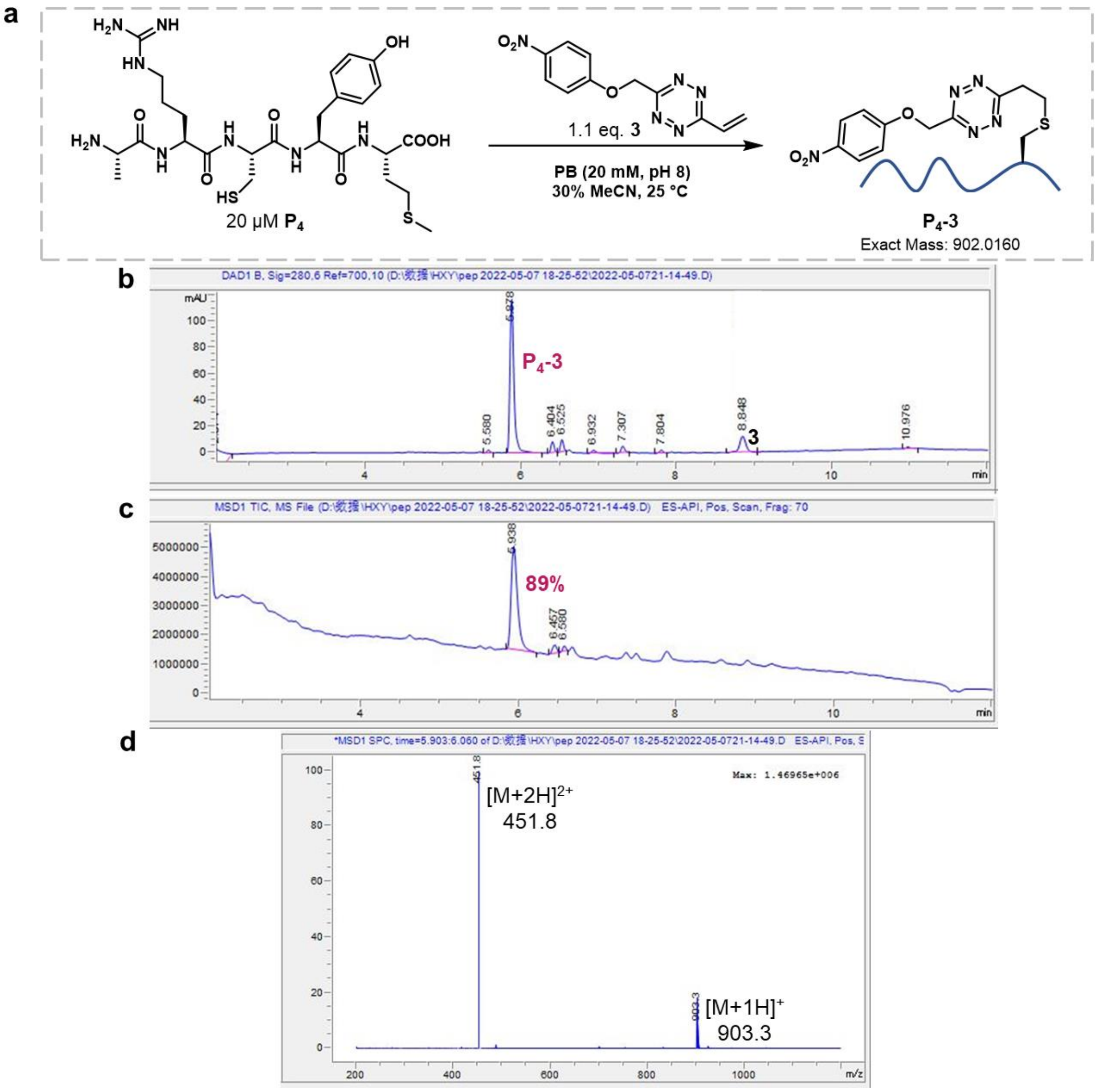

Supplementary Fig. 20. **Analysis the labeling reaction involving P4 with voTz 3 using LC–MS**. (a) The labeling reaction involving P4 and **3**. (b) HPLC–UV chromatogram at 280 nm of the reaction. (c) HPLC–MS chromatogram of the reaction. (d) The associated mass spectrum of the product P4-3.

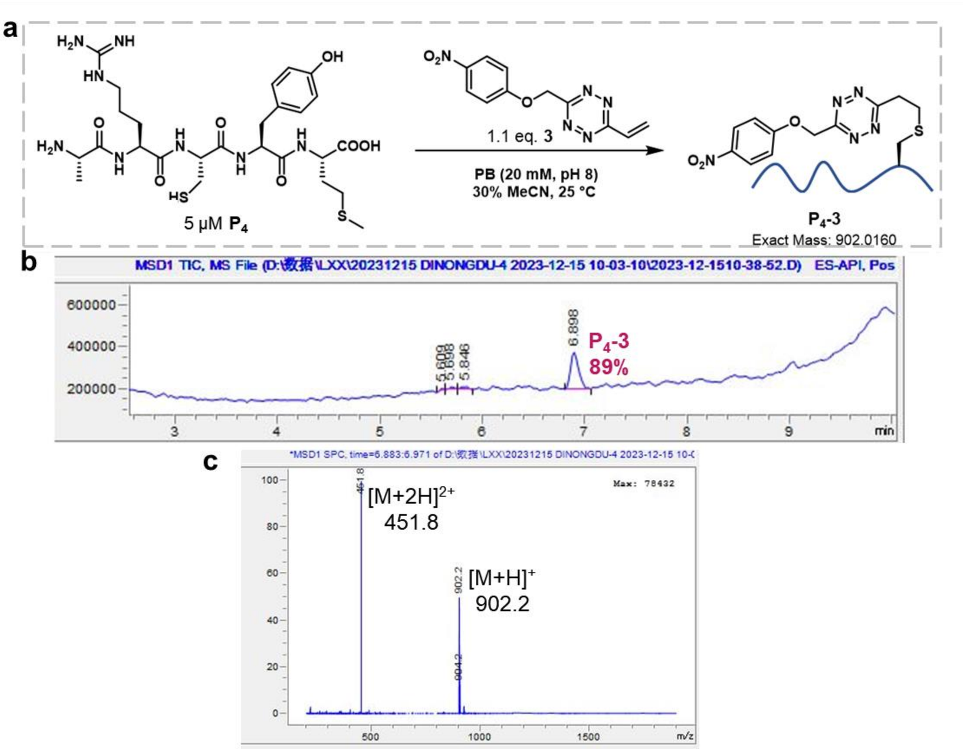

Supplementary Fig. 21. **Analysis the labeling reaction involving P4 with voTz 3 using LC–MS**. (a) The labeling reaction involving P4 and **3** at 5 μM. (b) HPLC–MS chromatogram of the reaction. (c) The associated mass spectrum of the product P4-3.

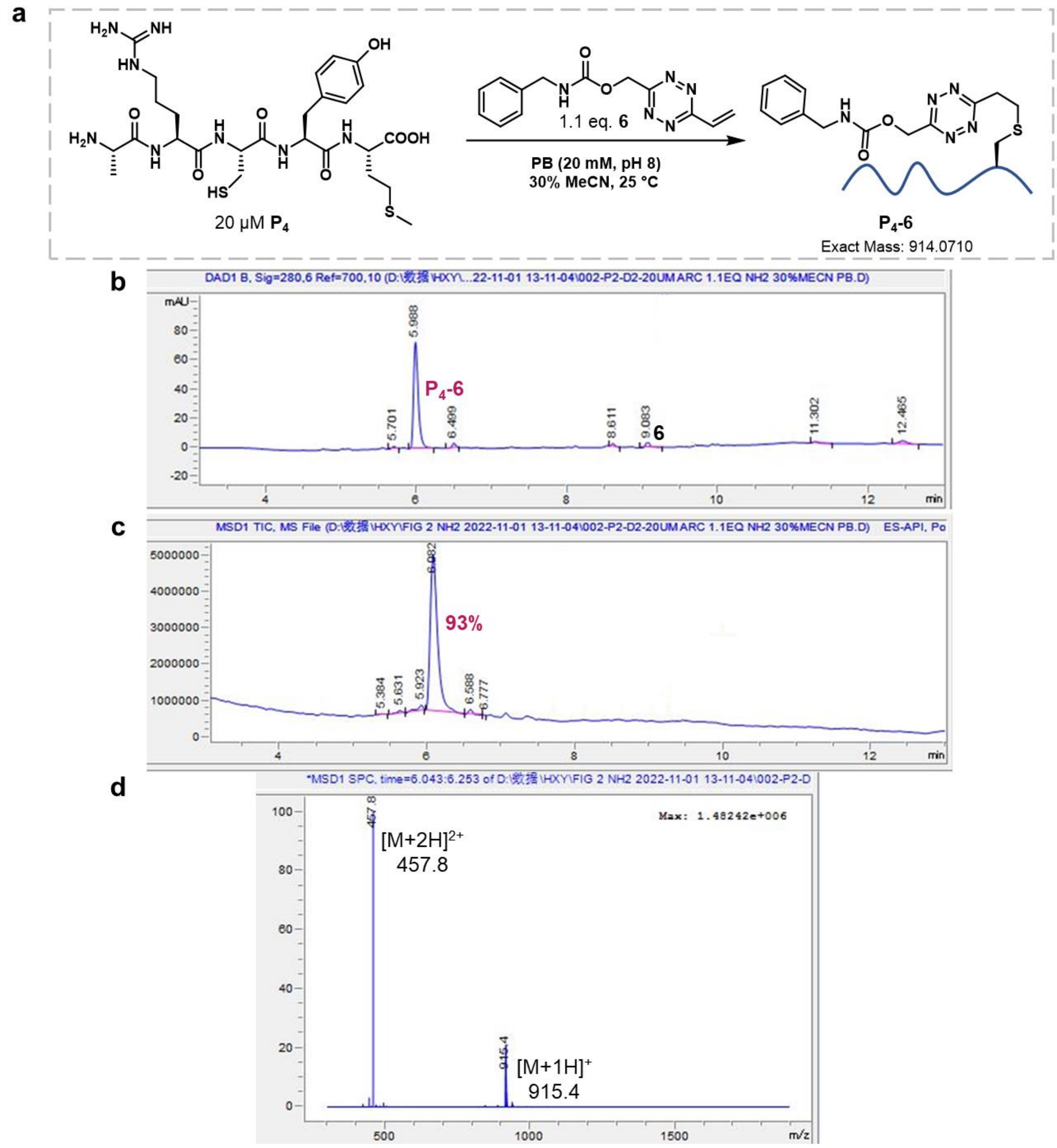

Supplementary Fig. 22. **Analysis the labeling reaction involving P$_4$ with voTz 6 using LC–MS**. (a) The labeling reaction involving P$_4$ and **6**. (b) HPLC–UV chromatogram at 280 nm of the reaction. (c) HPLC–MS chromatogram of the reaction. (d) The associated mass spectrum of the product P$_4$-6.

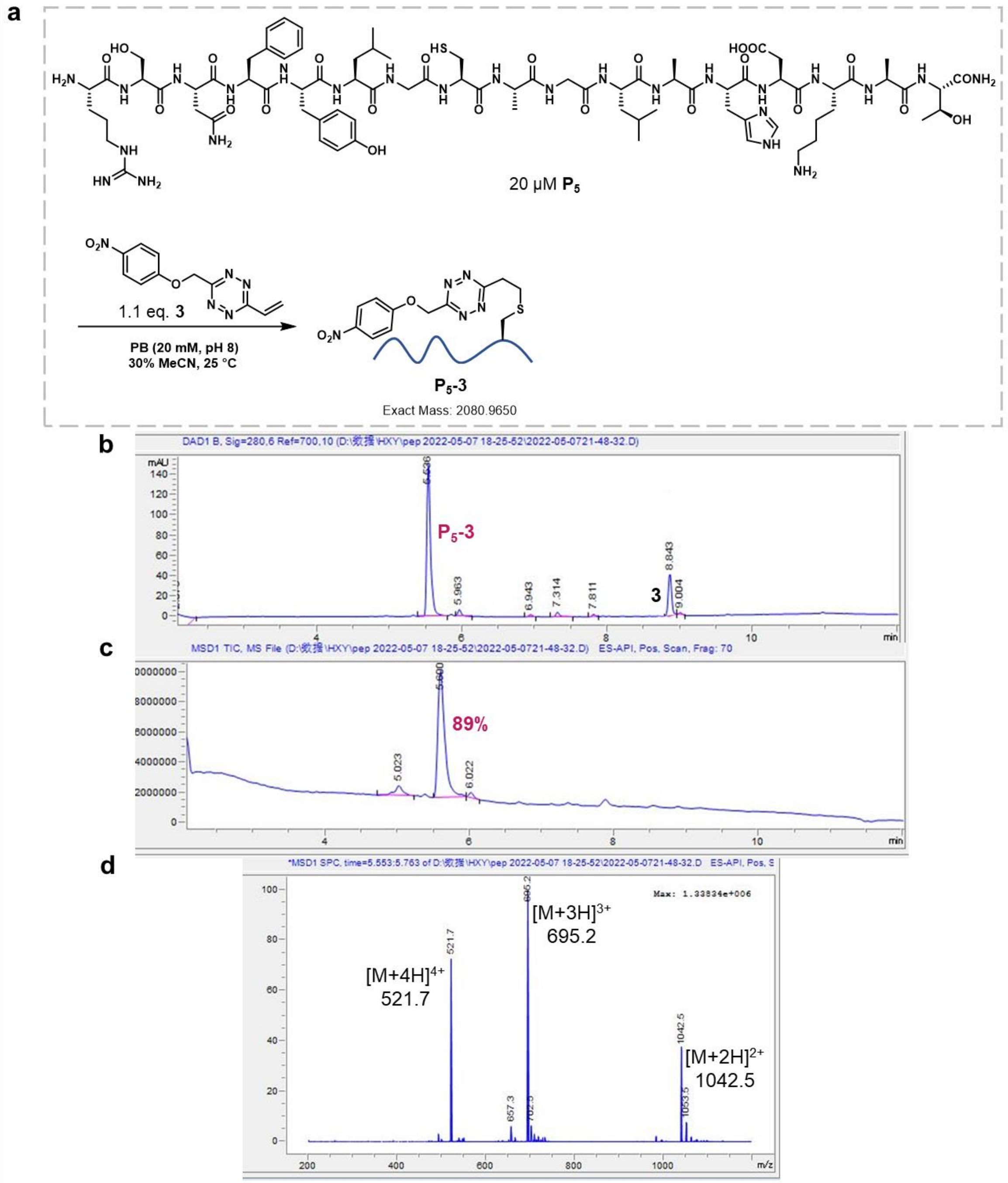

Supplementary Fig. 23. **Analysis the labeling reaction involving P$_5$ with voTz 3 using LC–MS**. (a) The labeling reaction involving P$_5$ and **3**. (b) HPLC–UV chromatogram at 280 nm of the reaction. (c) HPLC–MS chromatogram of the reaction. (d) The associated mass spectrum of the product P$_5$-3.

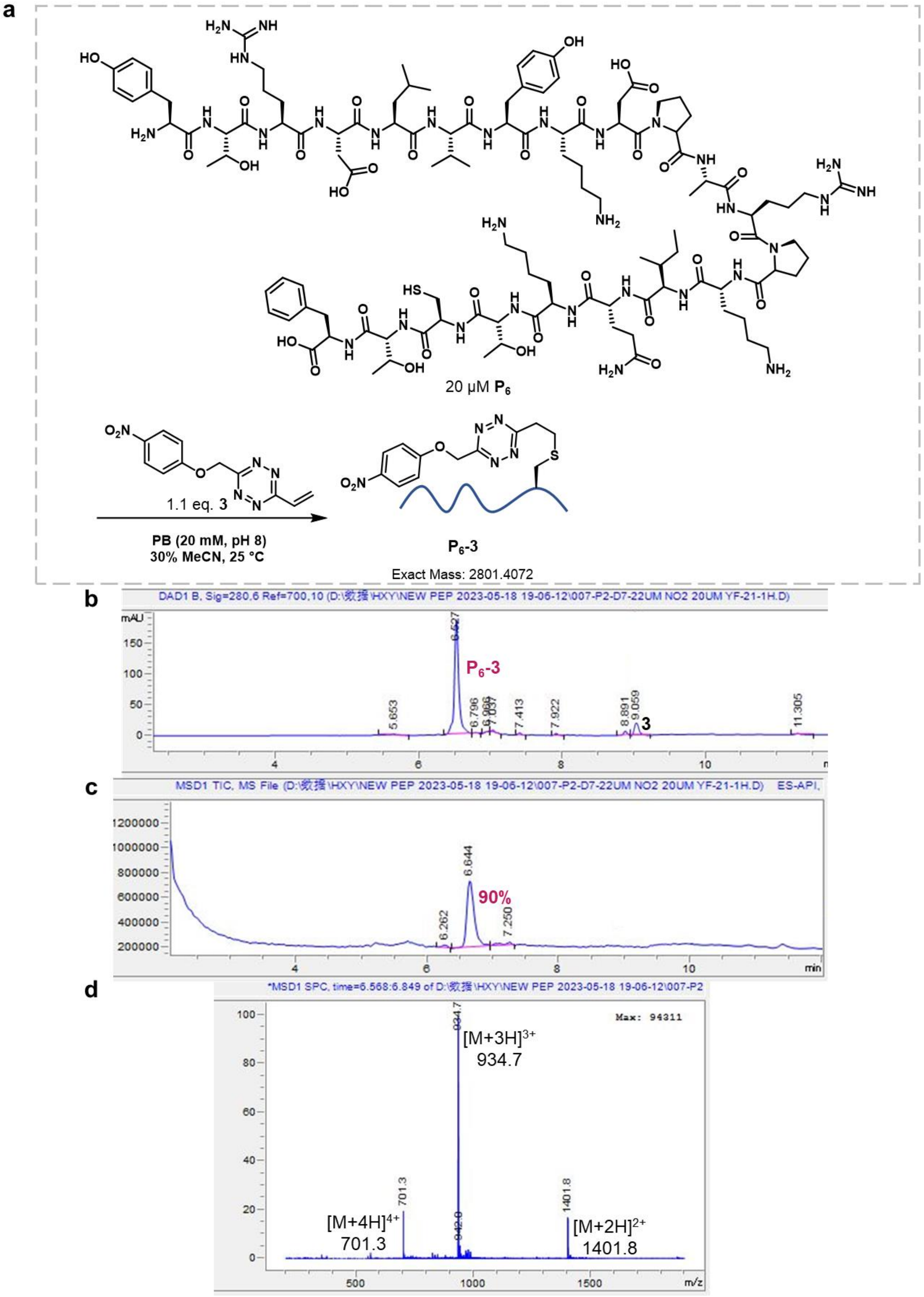

Supplementary Fig. 24. **Analysis the labeling reaction involving P_6 with voTz 3 using LC–MS**. (a) The labeling reaction involving P_6 and **3**. (b) HPLC–UV chromatogram at 280 nm of the reaction. (c) HPLC–MS chromatogram of the reaction. (d) The associated mass spectrum of the product P_6-3.

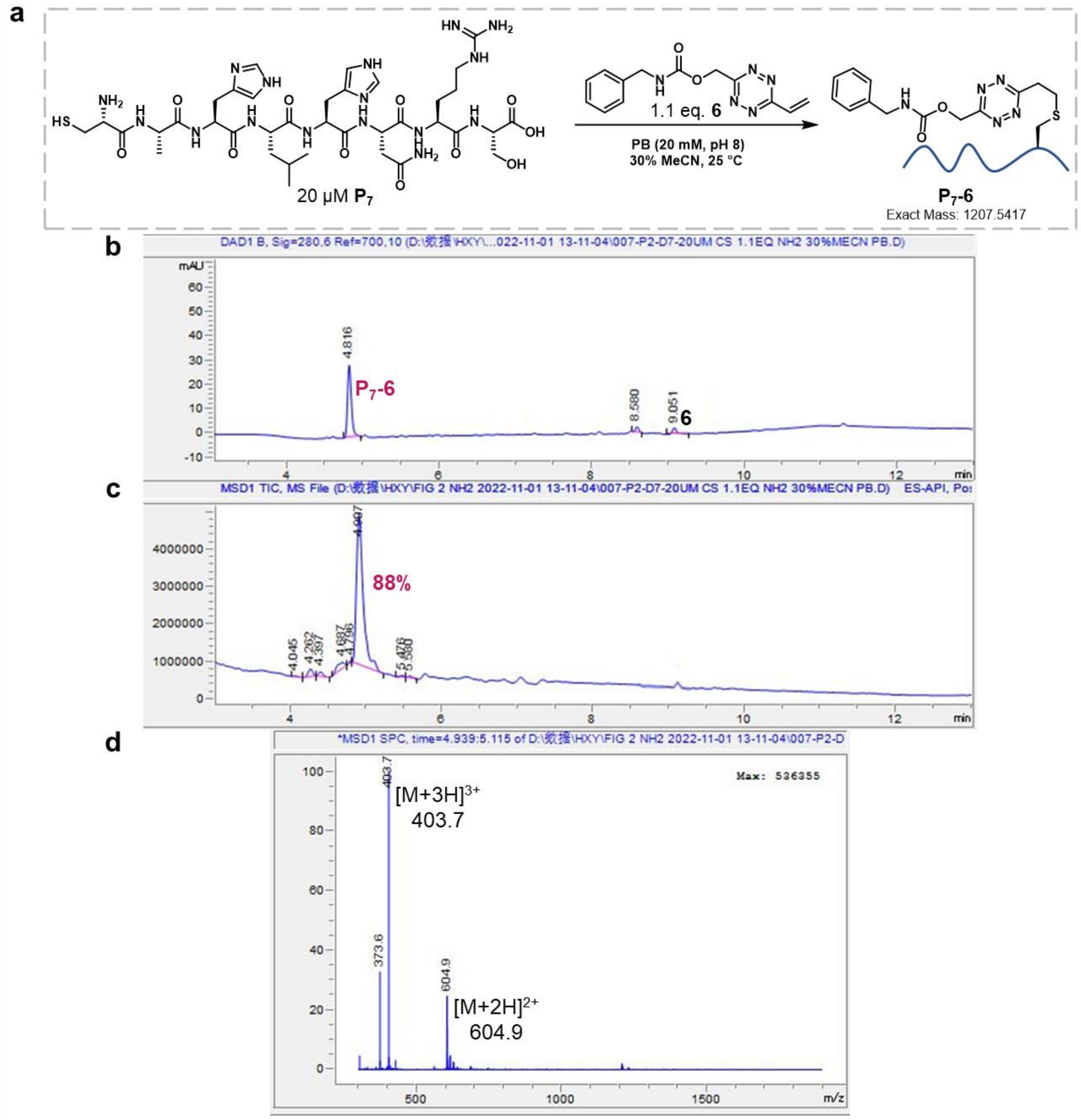

Supplementary Fig. 25. **Analysis the labeling reaction involving P$_7$ with voTz 6 using LC–MS**. (a) The labeling reaction involving P$_7$ and **6**. (b) HPLC–UV chromatogram at 280 nm of the reaction. (c) HPLC–MS chromatogram of the reaction. (d) The associated mass spectrum of the product P$_7$-6.

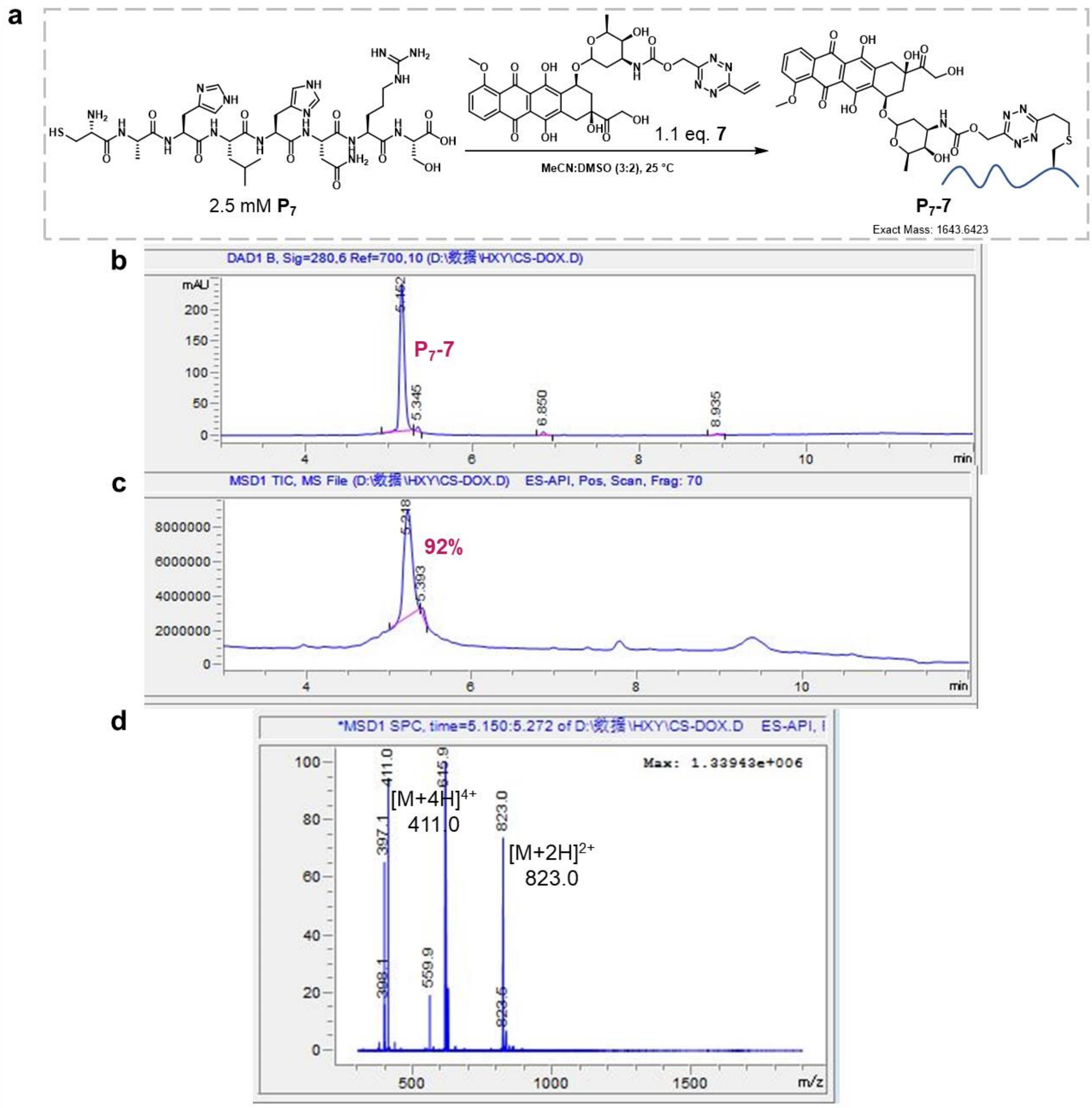

Supplementary Fig. 26. **Analysis the labeling reaction involving P7 with voTz 7 using LC–MS**. (a) The labeling reaction involving P7 and **7**. (b) HPLC–UV chromatogram at 280 nm of the reaction. (c) HPLC–MS chromatogram of the reaction. (d) The associated mass spectrum of the product P7-7.

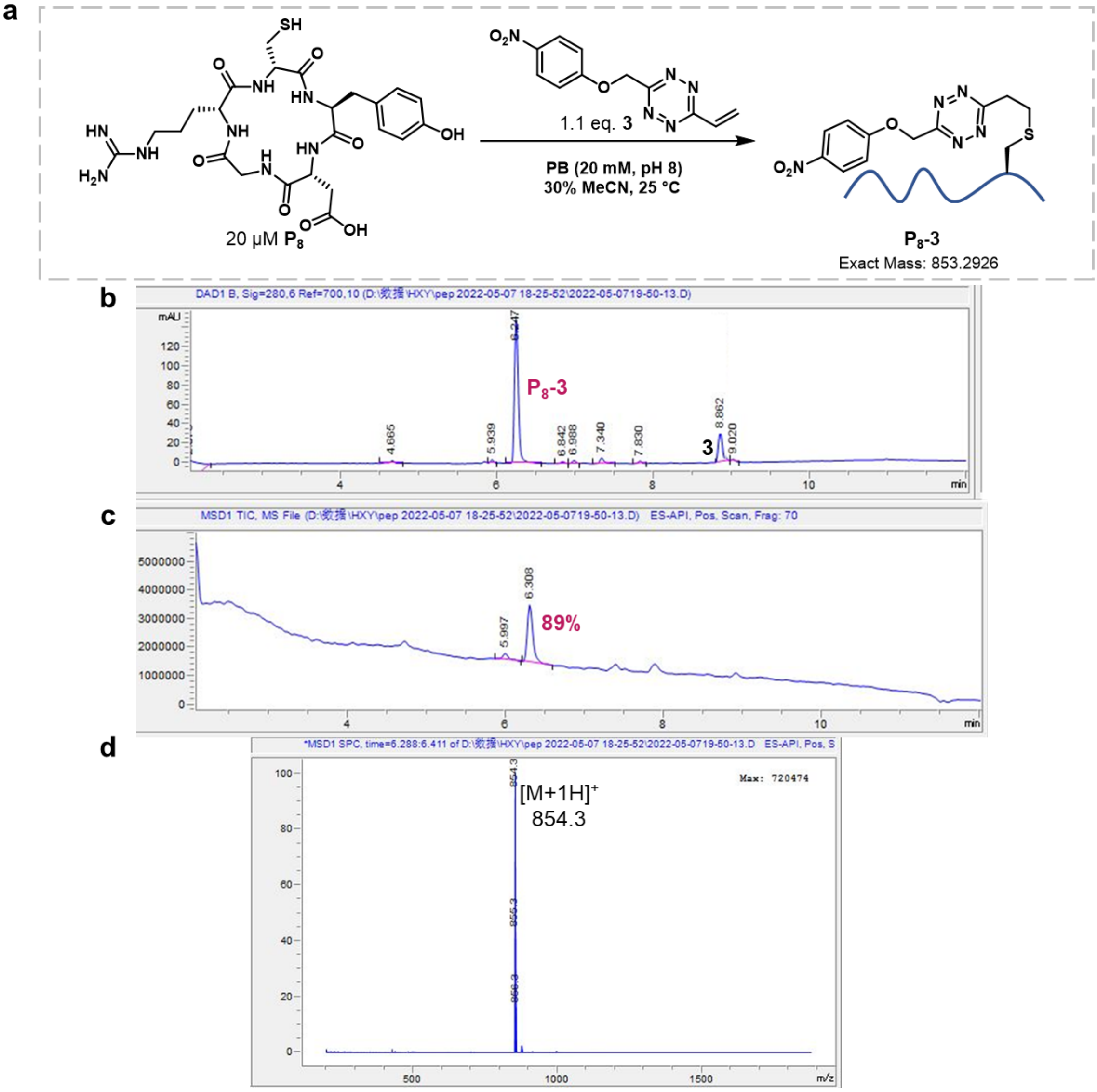

Supplementary Fig. 27. **Analysis the labeling reaction involving P_8 with voTz 3 using LC–MS**. (a) The labeling reaction involving P_8 and **3**. (b) HPLC–UV chromatogram at 280 nm of the reaction. (c) HPLC–MS chromatogram of the reaction. (d) The associated mass spectrum of the product P_8-3.

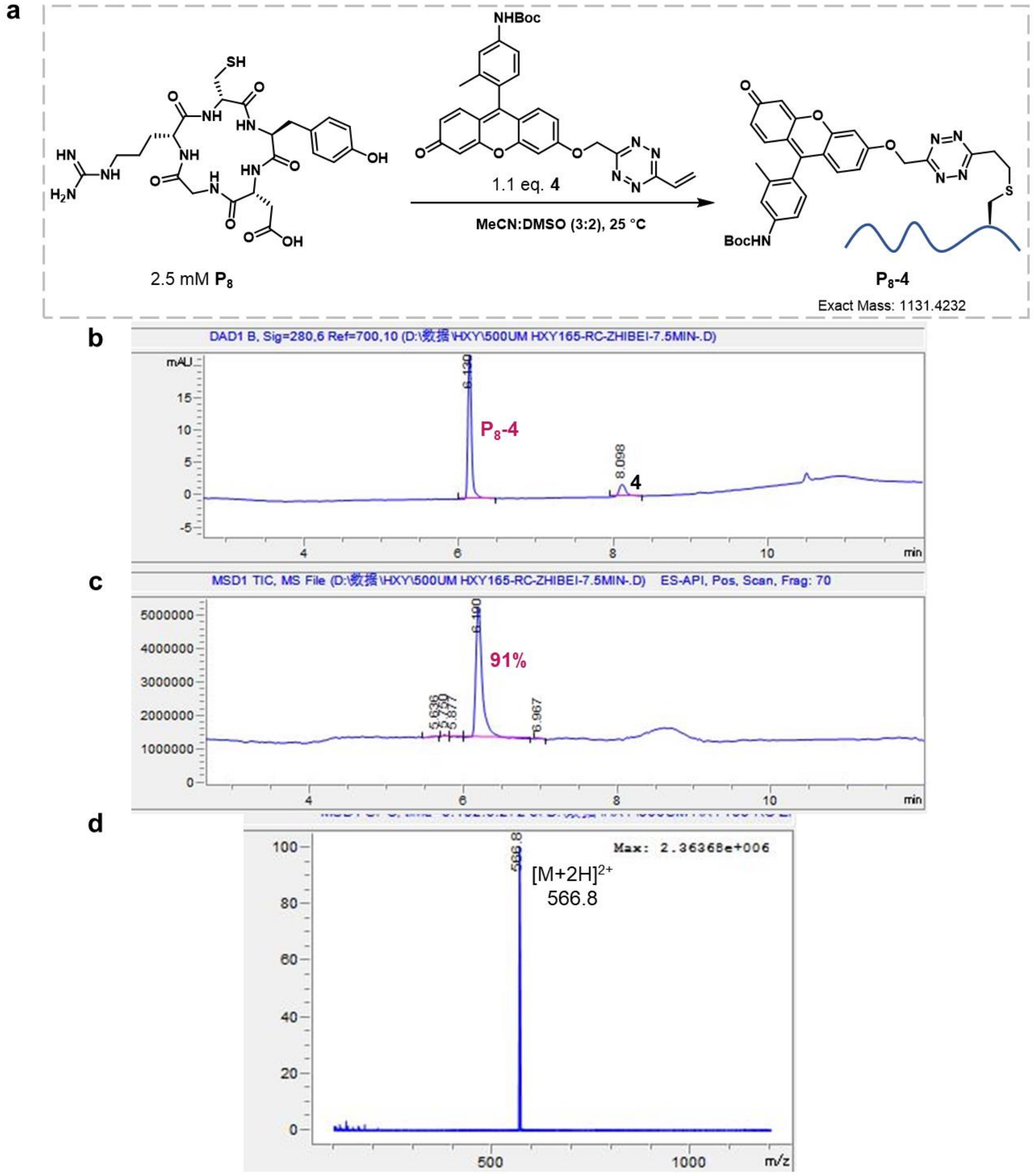

Supplementary Fig. 28. **Analysis the labeling reaction involving P8 with voTz 4 using LC–MS**. (a) The labeling reaction involving P8 and **4**. (b) HPLC–UV chromatogram at 280 nm of the reaction. (c) HPLC–MS chromatogram of the reaction. (d) The associated mass spectrum of the product P8-4.

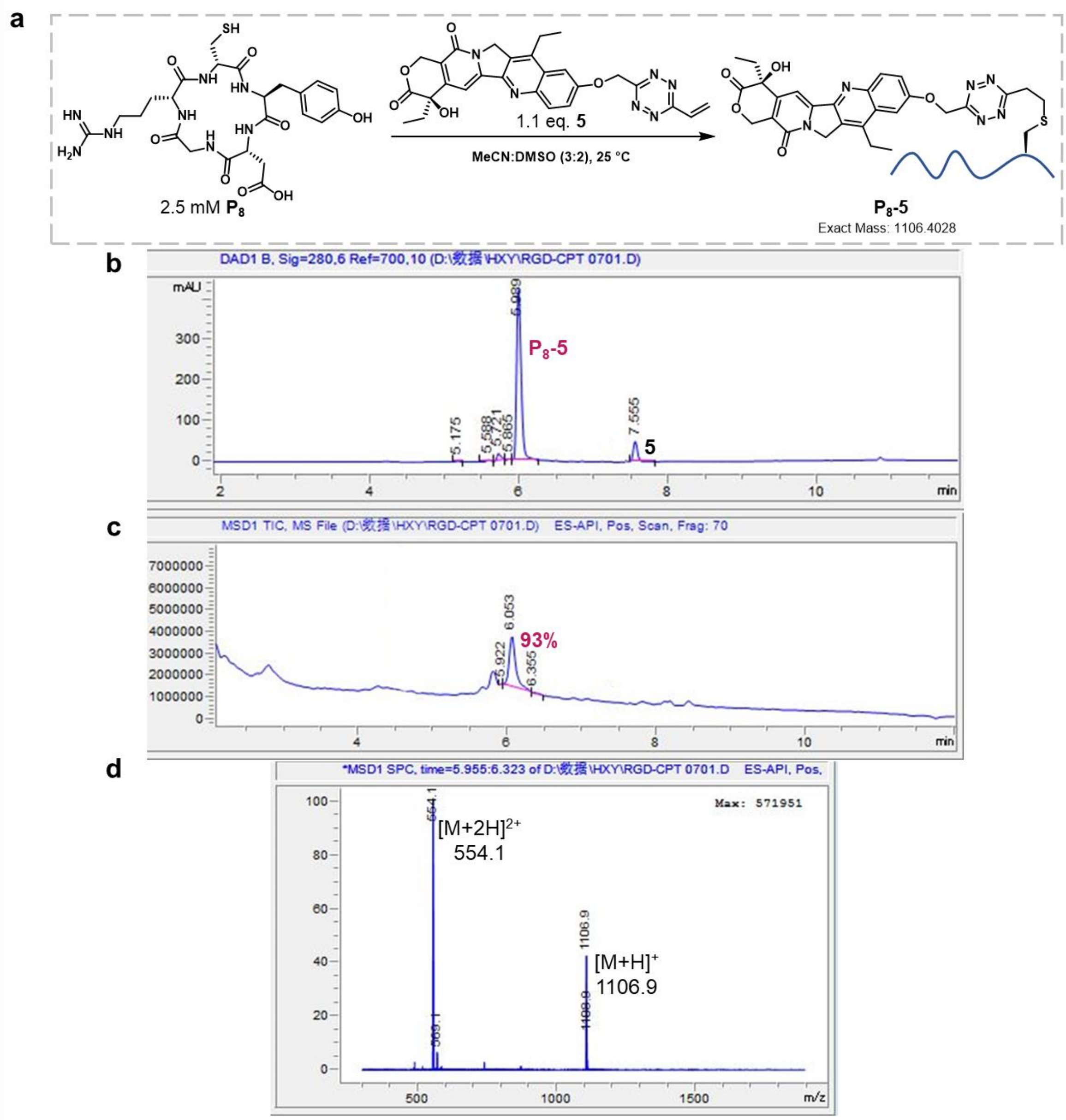

Supplementary Fig. 29. **Analysis the labeling reaction involving P_8 with voTz 5 using LC–MS**. (a) The labeling reaction involving P_8 and **5**. (b) HPLC–UV chromatogram at 280 nm of the reaction. (c) HPLC–MS chromatogram of the reaction. (d) The associated mass spectrum of the product P_8-5.

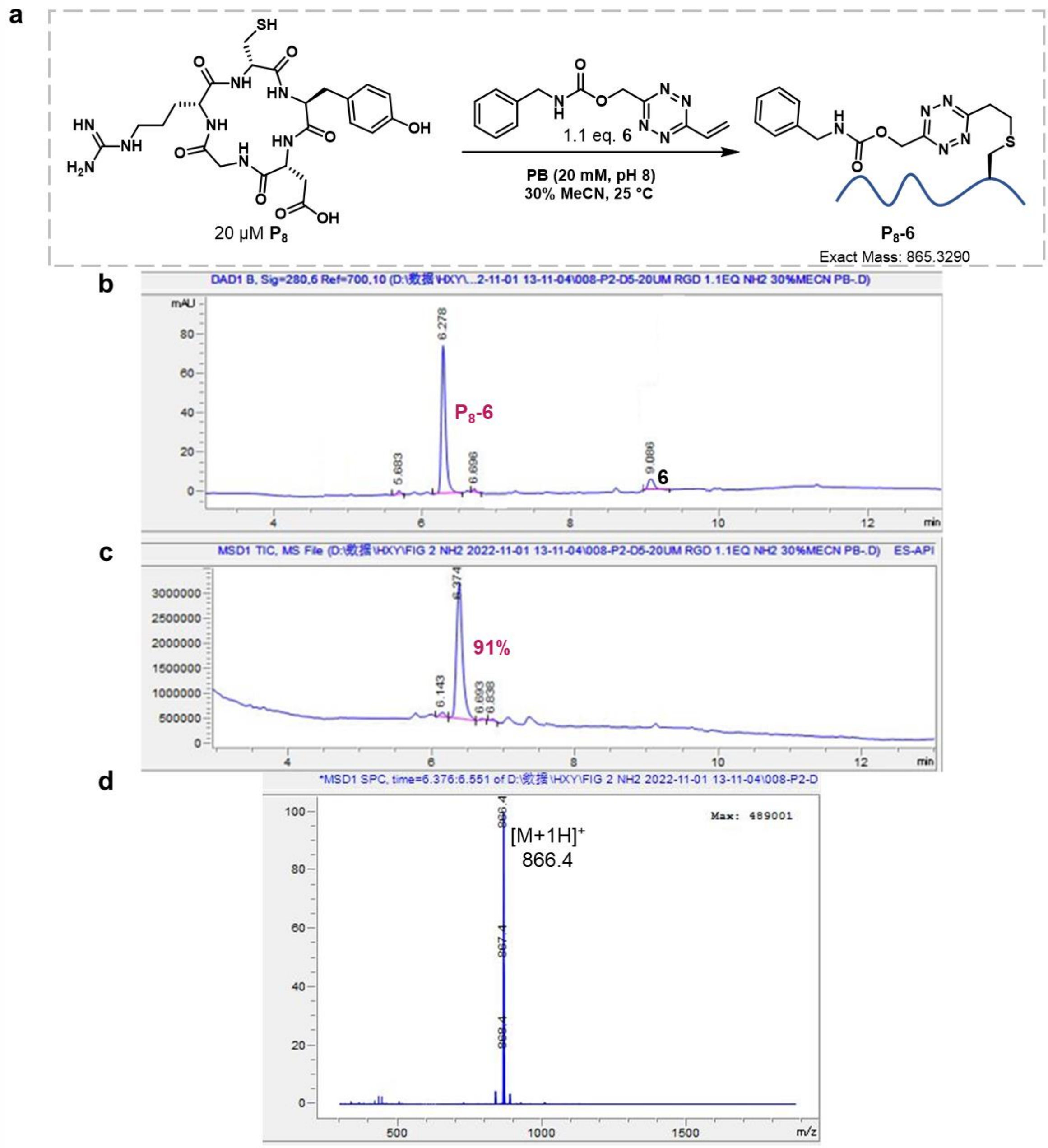

Supplementary Fig. 30. **Analysis the labeling reaction involving P8 with voTz 6 using LC–MS**. (a) The labeling reaction involving P8 and **6**. (b) HPLC–UV chromatogram at 280 nm of the reaction. (c) HPLC–MS chromatogram of the reaction. (d) The associated mass spectrum of the product P8-6.

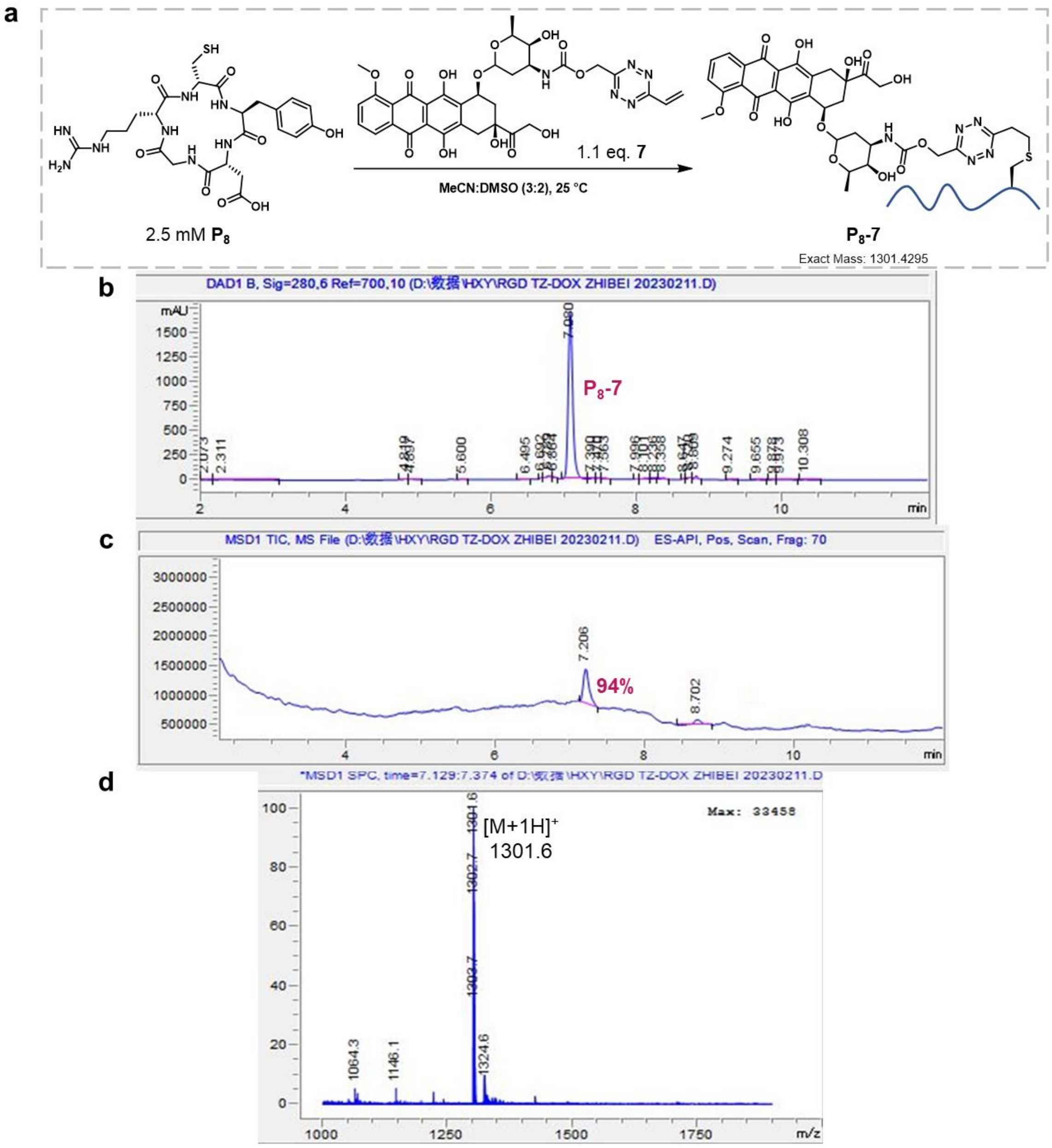

Supplementary Fig. 31. **Analysis the labeling reaction involving P8 with voTz 7 using LC–MS**. (a) The labeling reaction involving P8 and **7**. (b) HPLC–UV chromatogram at 280 nm of the reaction. (c) HPLC–MS chromatogram of the reaction. (d) The associated mass spectrum of the product P8-7.

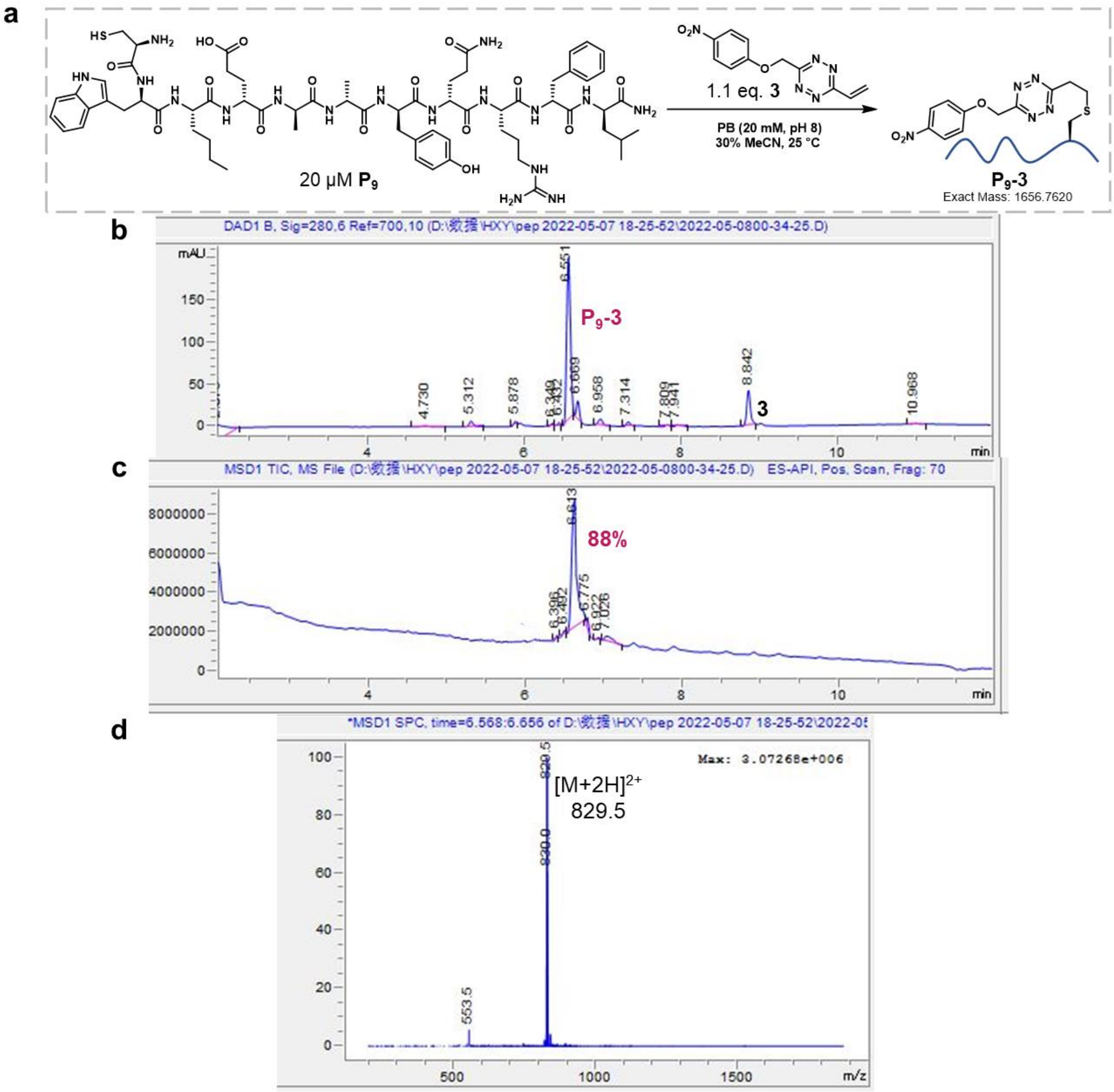

Supplementary Fig. 32. **Analysis the labeling reaction involving P_9 with voTz 3 using LC–MS**. (a) The labeling reaction involving P_9 and **3**. (b) HPLC–UV chromatogram at 280 nm of the reaction. (c) HPLC–MS chromatogram of the reaction. (d) The associated mass spectrum of the product P_9-3.

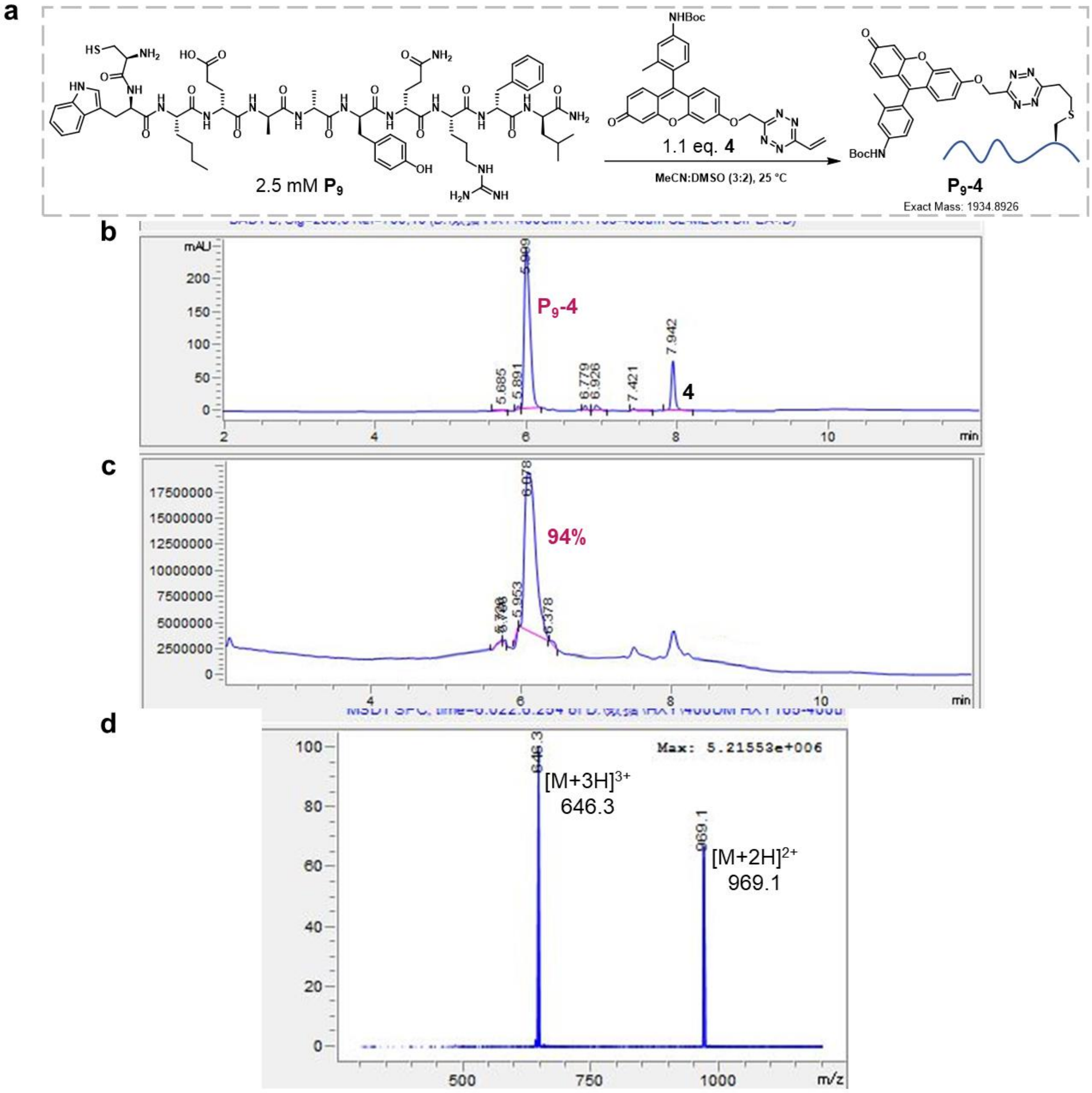

Supplementary Fig. 33. **Analysis the labeling reaction involving P9 with voTz 4 using LC–MS**. (a) The labeling reaction involving P9 and **4**. (b) HPLC–UV chromatogram at 280 nm of the reaction. (c) HPLC–MS chromatogram of the reaction. (d) The associated mass spectrum of the product P9-4.

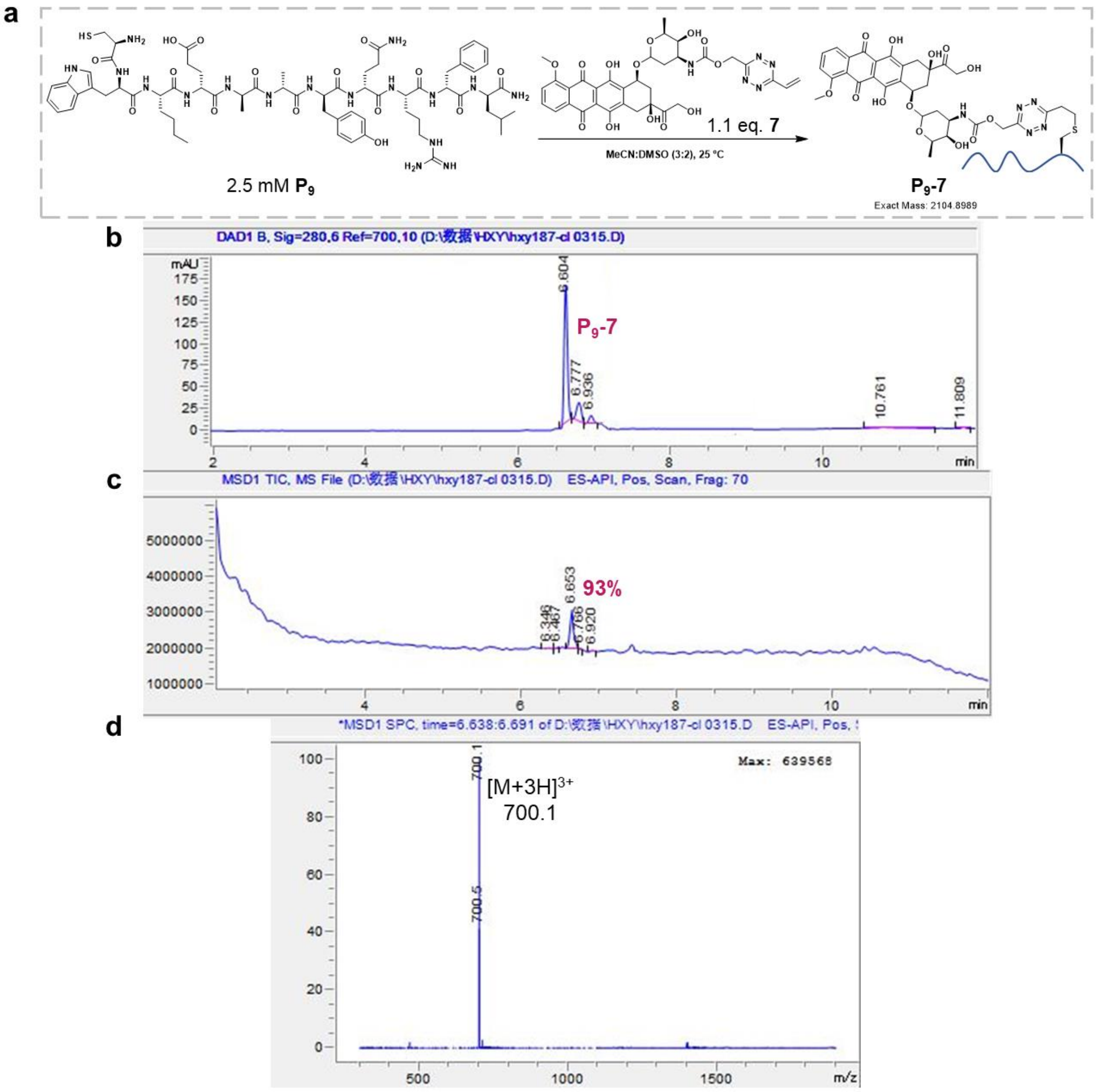

Supplementary Fig. 34. **Analysis the labeling reaction involving P_9 with voTz 7 using LC–MS**. (a) The labeling reaction involving P_9 and **7**. (b) HPLC–UV chromatogram at 280 nm of the reaction. (c) HPLC–MS chromatogram of the reaction. (d) The associated mass spectrum of the product P_9-7.

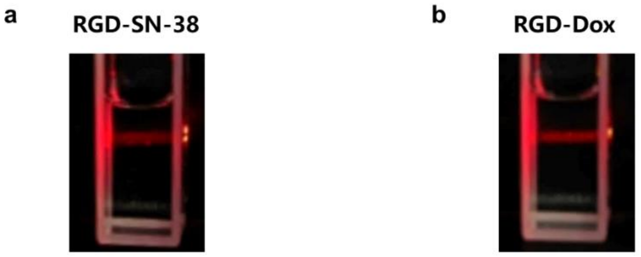

Supplementary Fig. 35. **Tyndall effect analyses**. Photographs of a red laser passing through the solutions (a) RGD-SN-38 and (b) RGD-Dox.

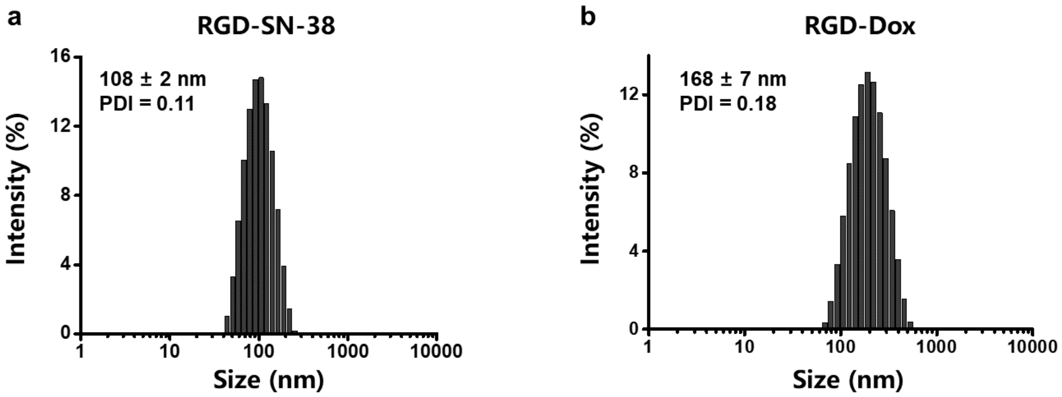

Supplementary Fig. 36. **Dynamic light scattering (DLS) measurements**. DLS analyses of the particle sizes of (a) RGD-SN-38 and (b) RGD-Dox. Independent measurements were taken three times. The results are shown as the mean ± SD.

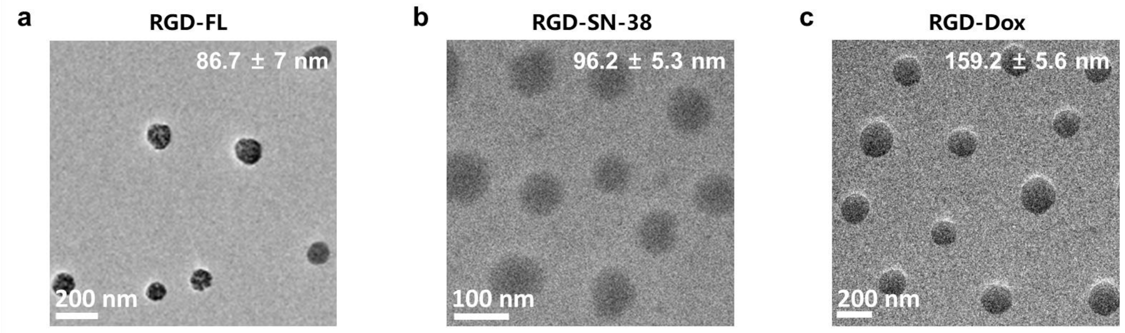

Supplementary Fig. 37. **TEM analyses**. TEM images and size distribution by TEM of self-assembly (a) RGD-FL, (b) RGD-SN-38, and (c) RGD-Dox. Images were analyzed using NIH ImageJ software to measure the diameter of individual particles. The mean size was calculated based on n = 30 particles. The results are shown as the mean ± SD.

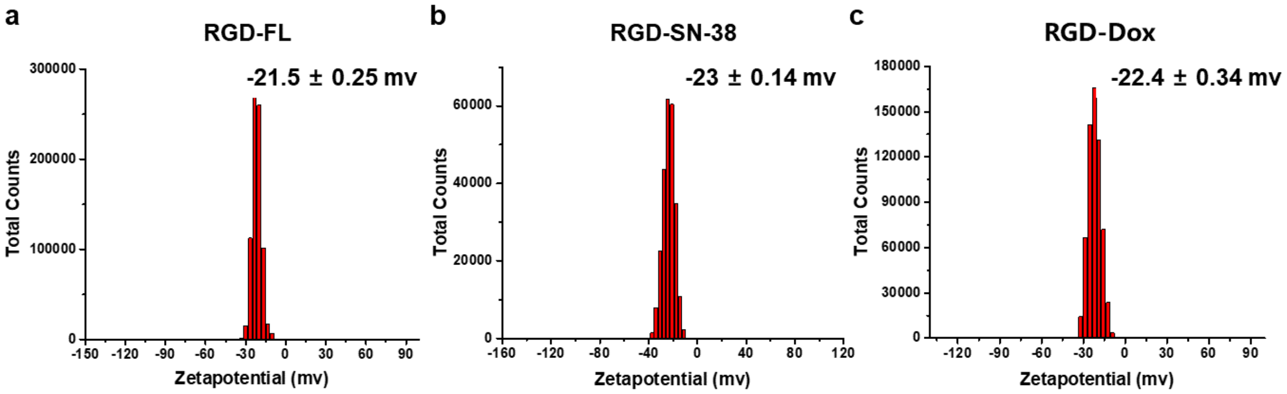

Supplementary Fig. 38. **The particle zeta potential values analyses**. Zeta potential values of (a) RGD-FL (b) RGD-SN-38 and (c) RGD-Dox. Independent measurements were taken three times. The results are shown as the mean ± SD.

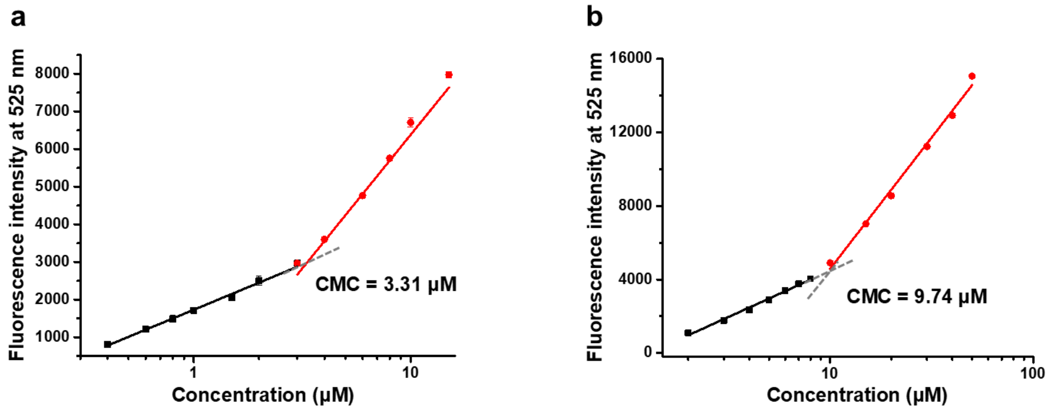

Supplementary Fig. 39. **The critical micelle concentration of peptide conjugates**. The critical aggregation concentration of (a) RGD-Dox in PBS was determined at 3.31 μM and (b) RGD-SN-38 in PBS was determined at 9.74 μM as the intersection of the tangents to the two linear segments of the graph.

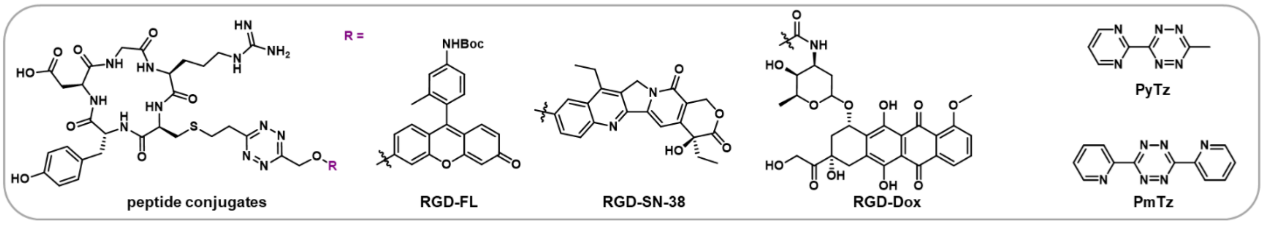

Supplementary Fig. 40. **The structures of tetrazine compounds**.

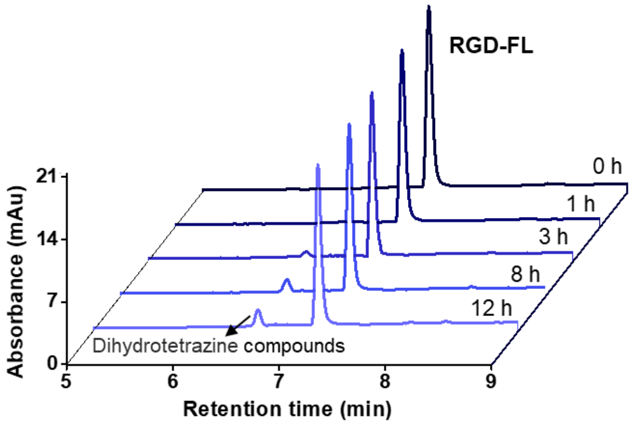

Time (h)	Stability (%)
0	100
1	96 ± 0.5
3	91 ± 2
8	88 ± 1
12	85 ± 3

Supplementary Fig. 41. **Stability of RGD-FL in the presence of a large excess of GSH**. Independent measurements were taken three times. The results are shown as the mean ± SD. Source data are provided as a Source Data file.

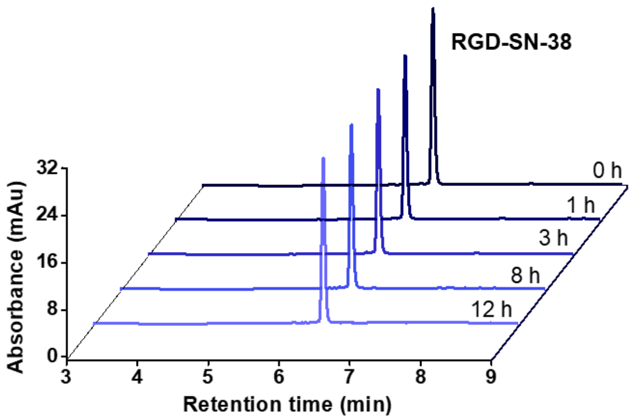

Time (h)	Stability (%)
0	100
1	96± 1
3	94 ± 2
8	93 ± 1
12	92 ± 2

Supplementary Fig. 42. **Stability of RGD-SN-38 in the presence of a large excess of GSH**. Independent measurements were taken three times. The results are shown as the mean ± SD. Source data are provided as a Source Data file.

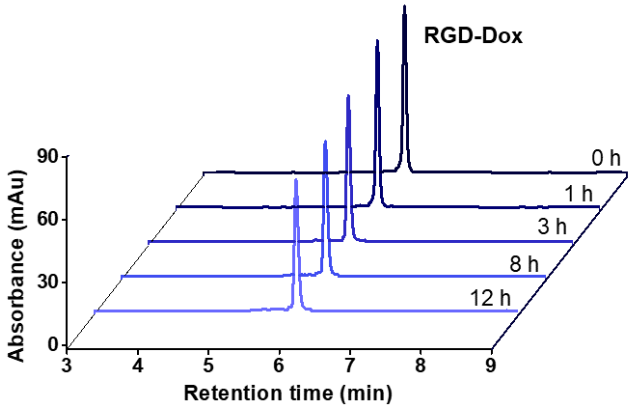

Time (h)	Stability (%)
0	100
1	98 ± 1
3	97 ± 1
8	96 ± 1
12	96 ± 1

Supplementary Fig. 43. **Stability of RGD-Dox in the presence of a large excess of GSH**. Independent measurements were taken three times. The results are shown as the mean ± SD. Source data are provided as a Source Data file.

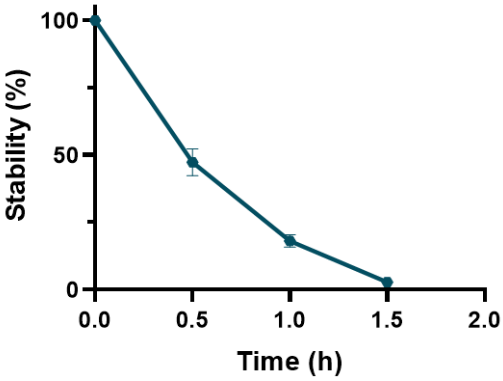

Time (h)	Stability (%)
0	100
0.5	47± 4
1	18 ± 2
1.5	3 ± 1

Supplementary Fig. 44. **Stability of PyTz in the presence of a large excess of GSH**. Independent measurements were taken three times. The results are shown as the mean ± SD. Source data are provided as a Source Data file.

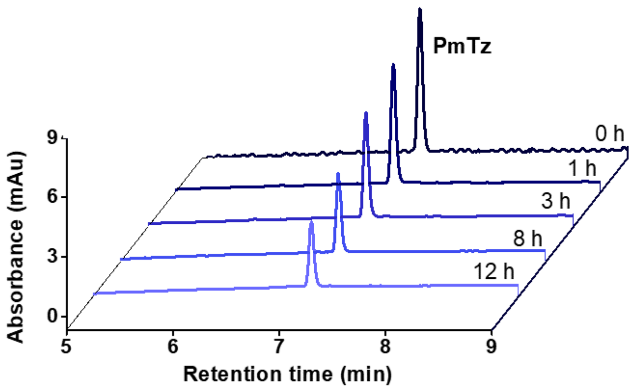

Time (h)	Stability (%)
0	100
1	86 ± 2
3	74 ± 2
8	51 ± 1
12	38 ± 2

Supplementary Fig. 45. **Stability of PmTz in the presence of a large excess of GSH**. Independent measurements were taken three times. The results are shown as the mean ± SD. Source data are provided as a Source Data file.

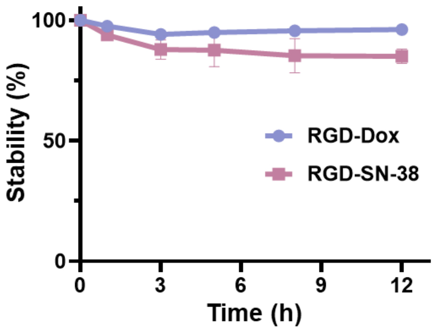

Time (h)	Stability (%)	
	RGD-Dox	RGD-SN-38
0	100	100
1	97 ± 1	94 ± 2
3	94 ± 1	88 ± 4
5	95 ± 0.1	87 ± 6
8	95 ± 0.5	85 ± 7
12	95 ± 1	85 ± 3

Supplementary Fig. 46. **Stability of RGD-Dox and RGD-SN-38 in the presence of a large excess of GSH at concentrations below their critical micelle concentrations (2 µM)**. Independent measurements were taken three times. The results are shown as the mean ± SD. Source data are provided as a Source Data file.

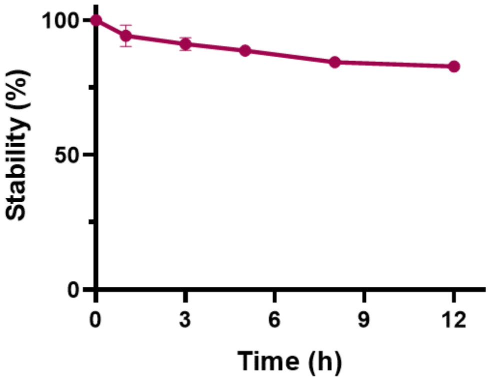

Time (h)	Stability (%)
0	100
1	94 ± 4
3	91 ± 2
5	89 ± 2
8	84 ± 2
12	82 ± 1

Supplementary Fig. 47. **Stability of RGD-SN-38 in the FBS**. RGD-SN-38 was diluted to a concentration of 50 µM using FBS (10% DMSO). The mixture was homogenized and incubated at 37 °C. The stability of RGD-SN-38 was determined by monitoring the decrease in peak intensity at 520 nm which was monitored by UV–Vis. Independent measurements were taken three times. The results are shown as the mean ± SD. Source data are provided as a Source Data file.

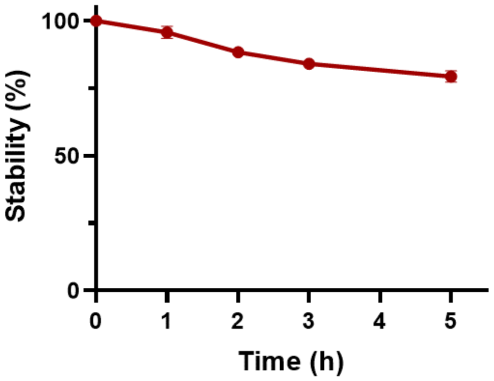

Time (h)	Stability (%)
0	100
1	96 ± 2
2	88 ± 1
3	84 ± 2
5	80 ± 2

Supplementary Fig. 48. **Stability of RGD-Dox in the 50% FBS**. RGD-Dox was diluted to a concentration of 50 µM using 50% FBS/PBS (10% DMSO). The mixture was homogenized and incubated at 37 °C. The stability of RGD-Dox was assessed by measuring the decrease in peak area at 520 nm (with the initial peak area at 0 min defined as 100%). This measurement was monitored by HPLC–MS. Independent measurements were taken three times. The results are shown as the mean ± SD. Source data are provided as a Source Data file.

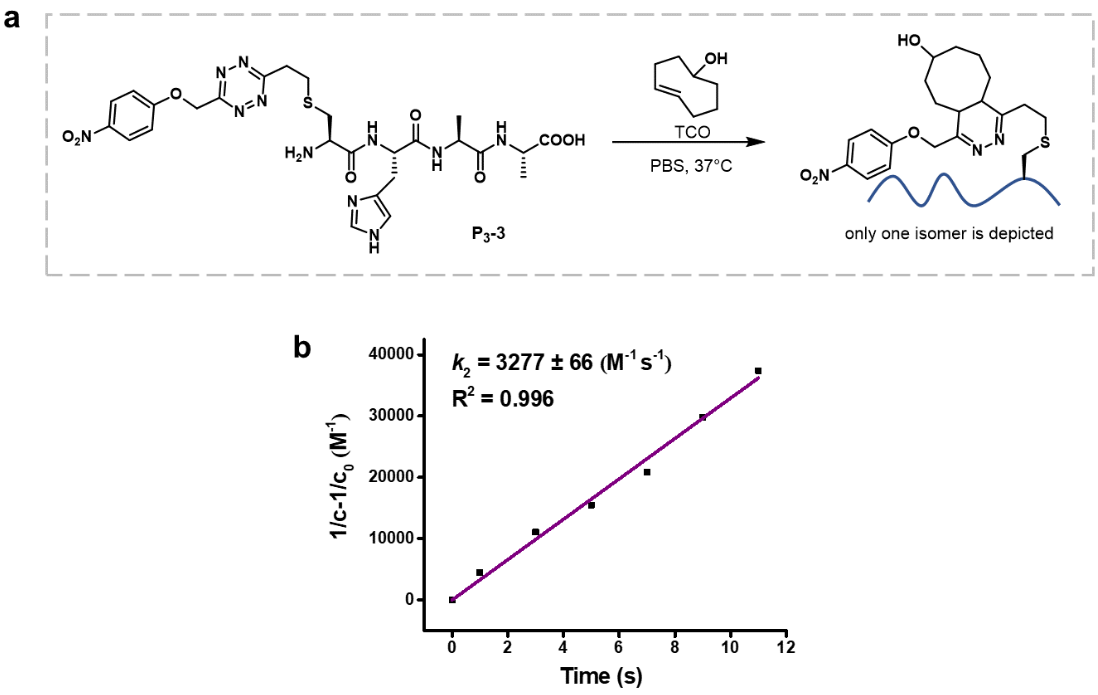

Supplementary Fig. 49. **Kinetic studies of bioorthogonal reactions between P3-3 and TCO**. (a) The bioorthogonal reaction between P3-3 and TCO in PBS at 37 °C (b) Calculation of the k_2 value of the reaction. The results are shown as the mean ± SD. Source data are provided as a Source Data file.

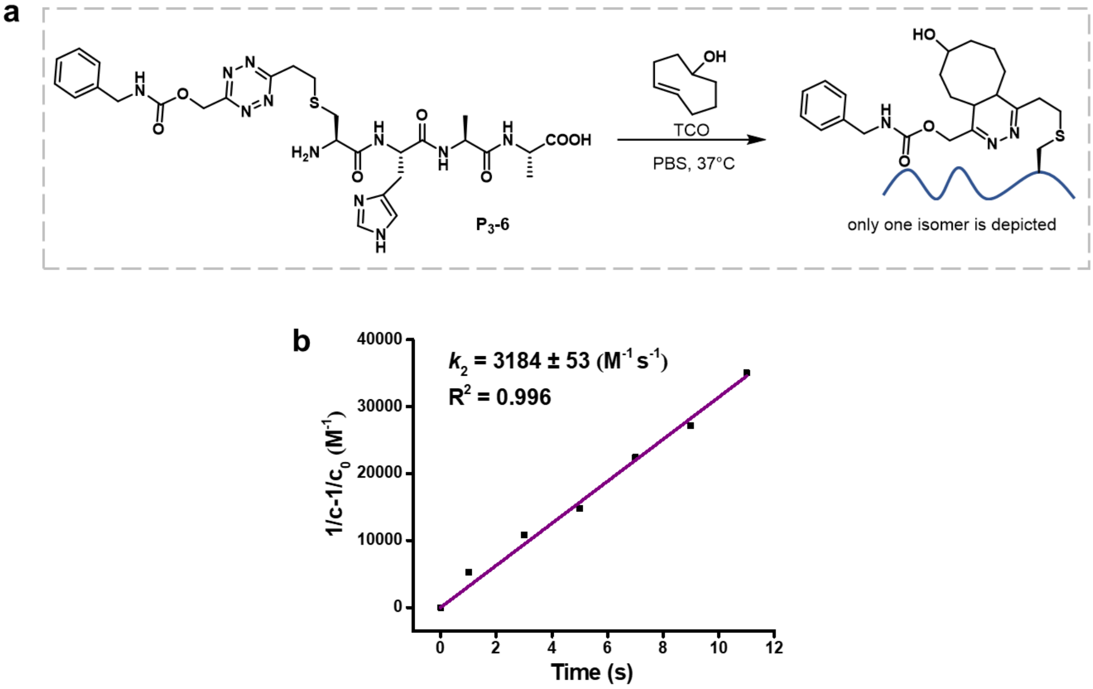

Supplementary Fig. 50. **Kinetic studies of bioorthogonal reactions between P3-6 and TCO**. (a) The bioorthogonal reaction between P3-6 and TCO in PBS at 37 °C (b) Calculation of the k_2 value of the reaction. The results are shown as the mean ± SD. Source data are provided as a Source Data file.

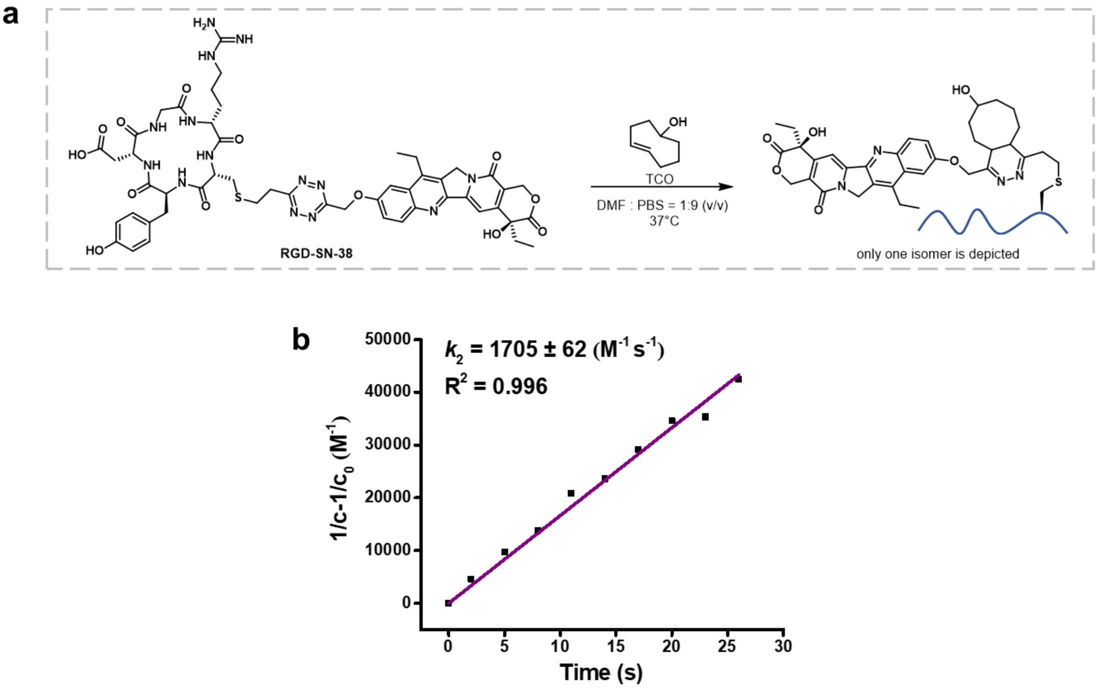

Supplementary Fig. 51. **Kinetic studies of bioorthogonal reactions between RGD-SN-38 and TCO**. (a) The bioorthogonal reaction between RGD-SN-38 and TCO in PBS (10% DMF) at 37 ℃. (b) Calculation of the k_2 value of the reaction. The results are shown as the mean ± SD. Source data are provided as a Source Data file.

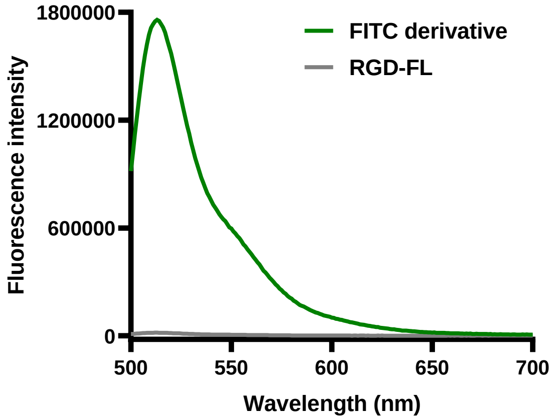

Supplementary Fig. 52. **Fuorescence spectra of RGD-FL and FITC derivative**. The RGD-FL or FITC derivative was dissolved in PBS (1% DMSO) to obtain a 2 µM solution. λ_{ex} = 495 nm.

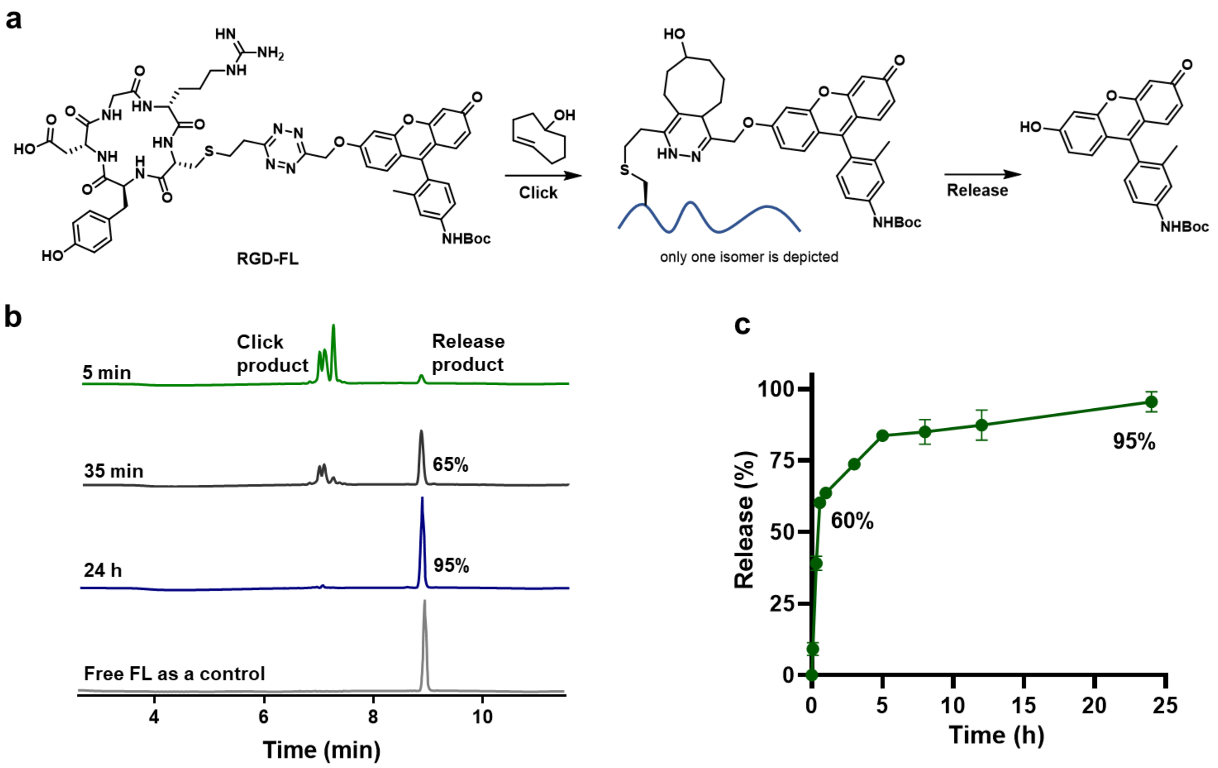

Supplementary Fig. 53. ***In vitro* release analysis of RGD-FL with TCO**. (a) The reaction between 50 µM RGD-FL and 500 µM TCO in PBS (1% DMSO) at 37 °C. (b) HPLC traces of the reaction at different time points, with detection at 495 nm. (c) The release progress were quantified by HPLC-MS at a wavelength of 495 nm. The results are shown as the mean ± SD. Source data are provided as a Source Data file.

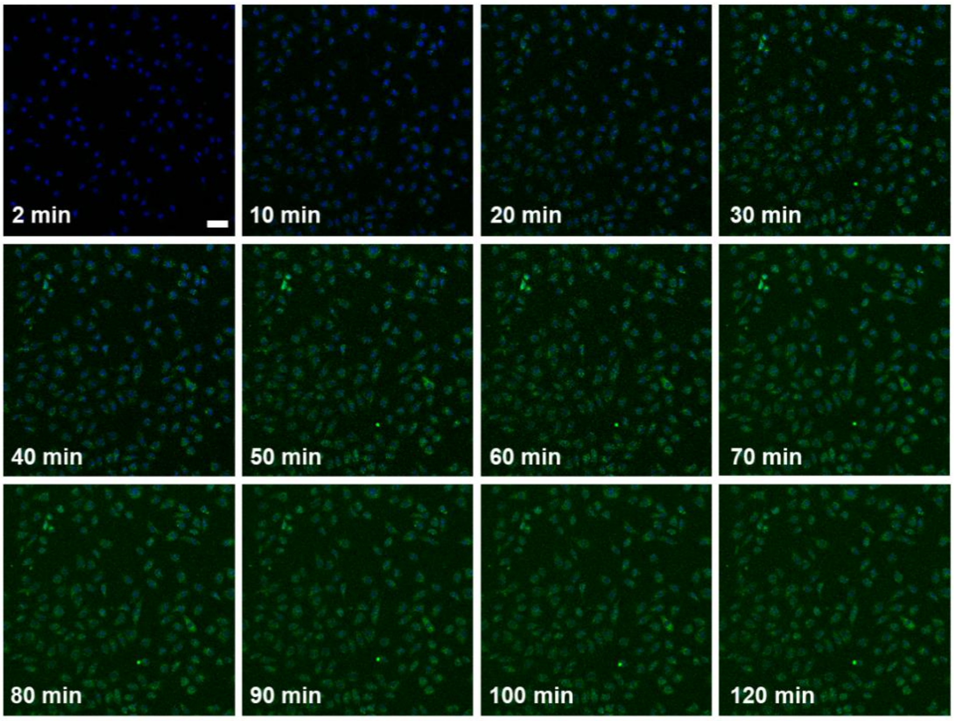

Supplementary Fig. 54. **Time-lapse imaging of RGD-FL with TCO in live SKOV3 cells**. Scale bar: 50 μm.

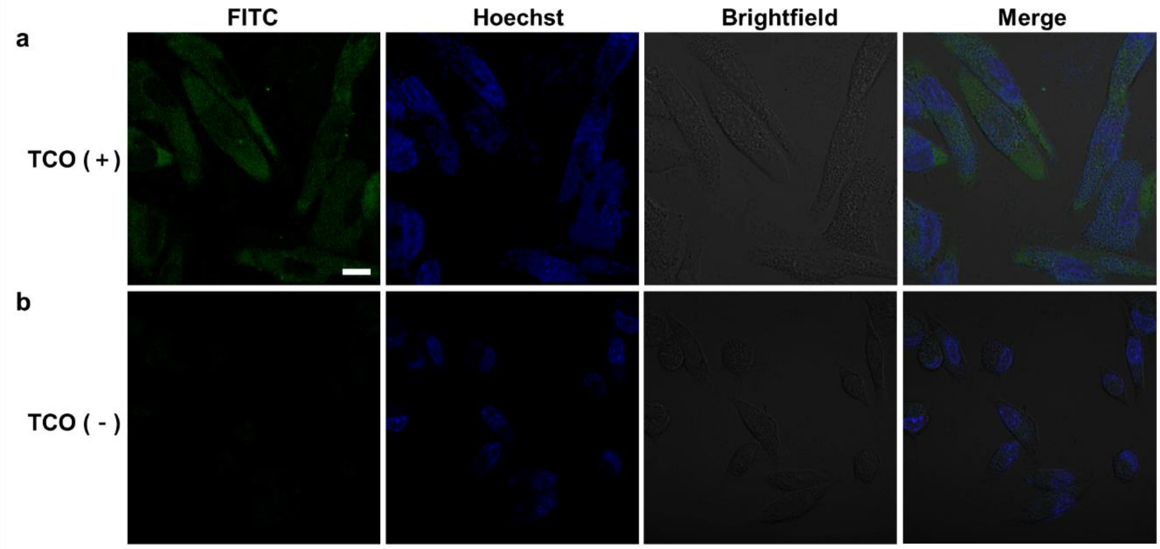

Supplementary Fig. 55. **Fluorescence imaging of SKOV3 cells using RGD-FL**. (a) With TCO. (b) Without TCO. Scale bar: 10 μm.

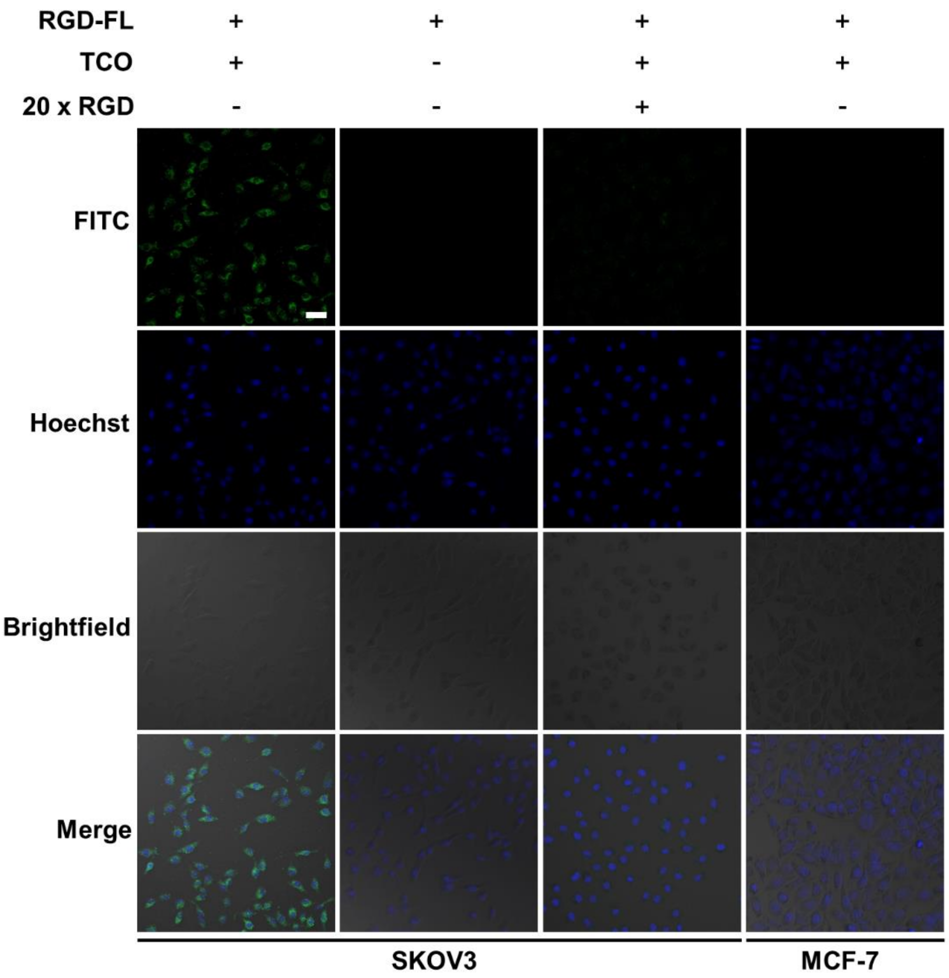

Supplementary Fig. 56. **Targeting study of RGD-FL in live cells**. Fluorescence imaging of SKOV3 and MCF-7 cells. Scale bar: 50 μm.

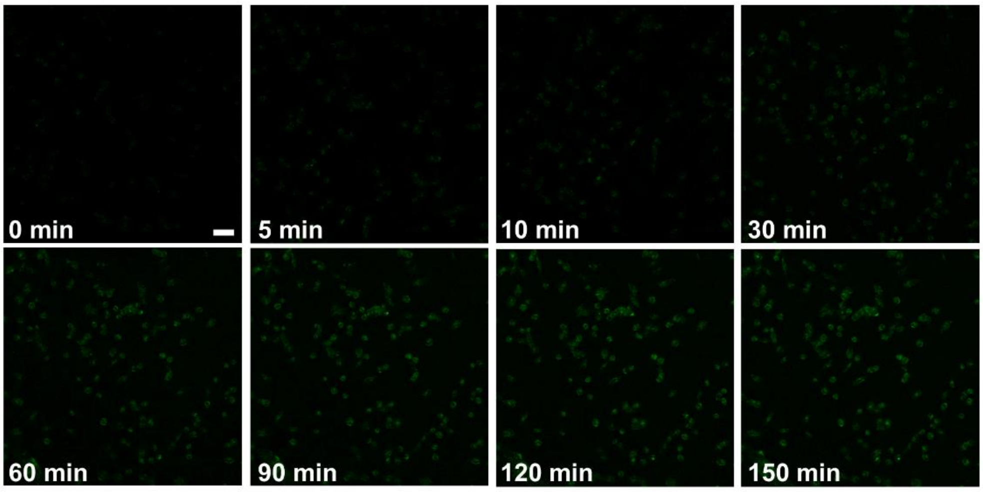

Supplementary Fig. 57. **Time-lapse imaging of 18-4-FL with TCO in live MDA-MB-231 cells**. Scale bar: 50 μm.

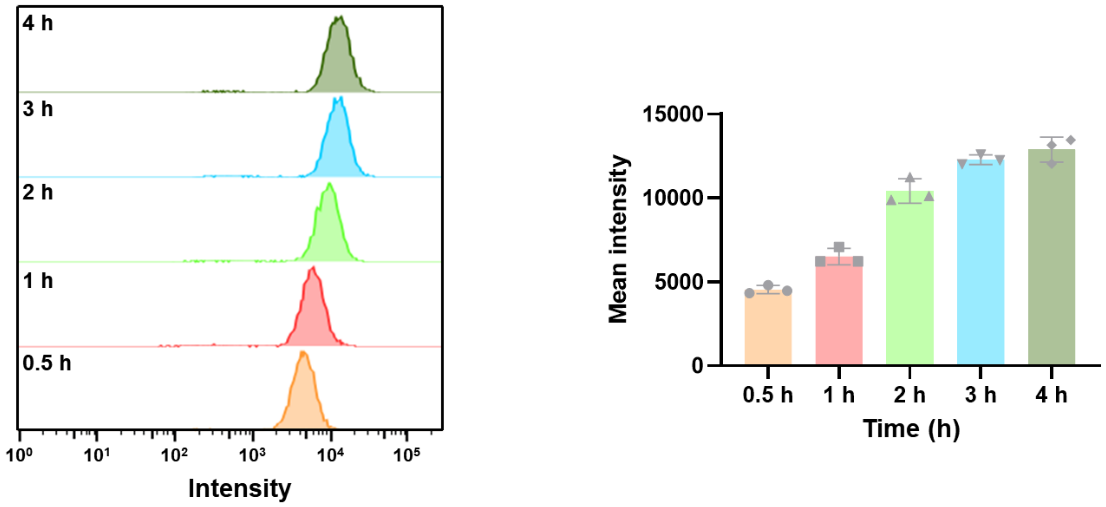

Supplementary Fig. 58. Flow cytometry assessment of cellular uptake of RGD-Dox by B16F10 cells at different time points. Data are presented as mean ± SD (n = 3 biologically independent samples). Source data are provided as a Source Data file.

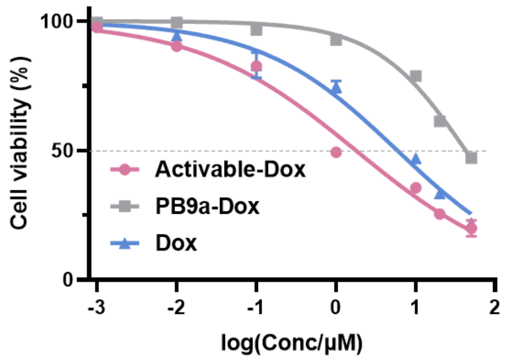

Supplementary Fig. 59. **Cytotoxicity of PB9a-Dox against HepG2 cell lines**. Grey and blue lines show cells incubated with PB9a-Dox or Dox of various concentrations for 48 h. The red line show cells were pre-incubated with different concentrations of PB9a-Dox for 2 h followed by incubation with TCO for 48 h in total. Data are presented as mean ± SD (n = 3 biologically independent samples). Source data are provided as a Source Data file.

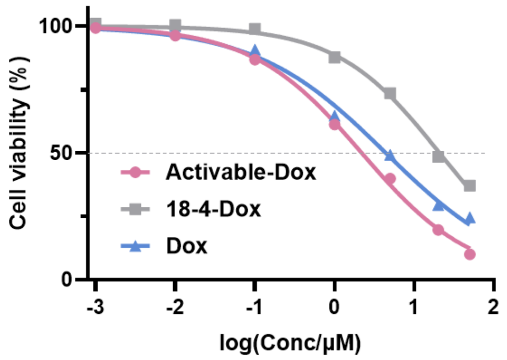

Supplementary Fig. 60. **Cytotoxicity of 18-4-Dox against MDA-MB-231 cell lines**. Grey and blue lines represent cells incubated with 18-4-Dox or Dox at various concentrations for 48 h. The red line represents cells pre-incubated with different concentrations of 18-4-Dox for 2 h, followed by incubation with TCO for a total of 48 h. Data are presented as mean ± SD (n = 3 biologically independent samples). Source data are provided as a Source Data file.

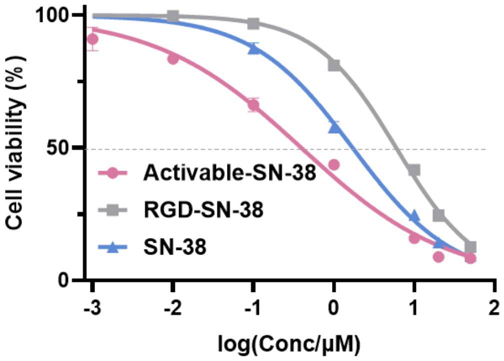

Supplementary Fig. 61. **Cytotoxicity of RGD-SN-38 against U87 cell lines**. Grey and blue lines represent cells incubated with RGD-SN-38 or SN-38 of various concentrations for 48 h. The red line represents cells were pre-incubated with different concentrations of RGD-SN-38 for 2 h, followed by incubation with TCO for a total of 48 h. Data are presented as mean ± SD (n = 3 biologically independent samples). Source data are provided as a Source Data file.

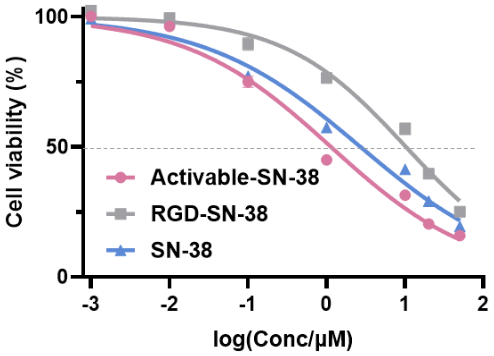

Supplementary Fig. 62. **Cytotoxicity of RGD-SN-38 against SKOV3 cell lines**. Grey and blue lines represent cells incubated with RGD-SN-38 or SN-38 of various concentrations for 48 h. The red line represents cells were pre-incubated with different concentrations of RGD-SN-38 for 2 h, followed by incubation with TCO for a total of 48 h. Data are presented as mean ± SD (n = 3 biologically independent samples). Source data are provided as a Source Data file.

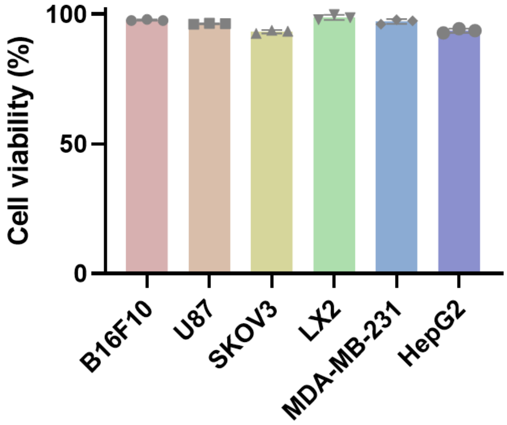

Supplementary Fig. 63. **The 48 h cytotoxicity of TCO against B16F10, U87, SKOV3, LX2, MDA-MB-231 and HepG2 cell lines**. Data are presented as mean ± SD (n = 3 biologically independent samples). Source data are provided as a Source Data file.

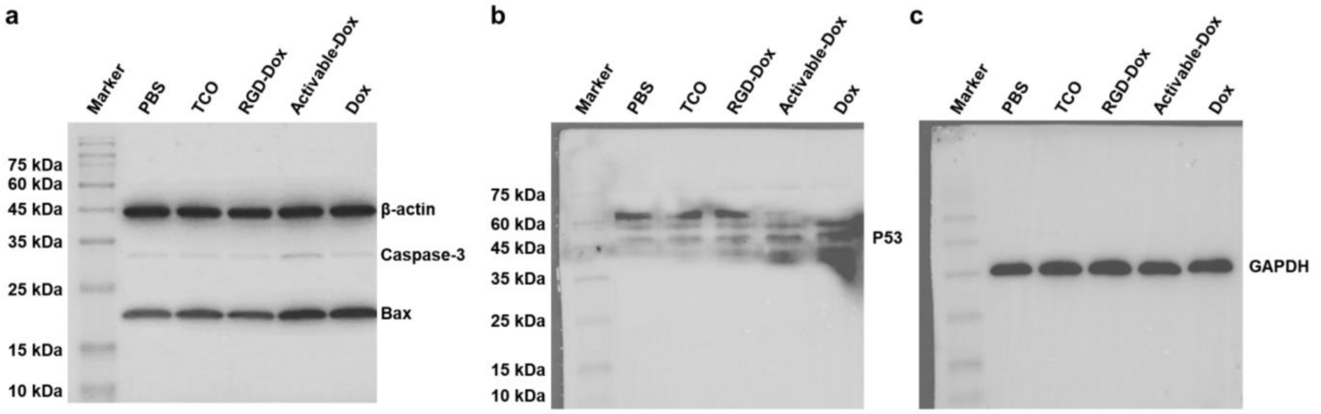

Supplementary Fig. 64. **Western blotting analysis**. Western blotting analysis of (a) Bax, Caspase-3, and (b, c) p53 expression in B16F10 cells after treatment for 24 h (figures b and c were derived from the same membrane).

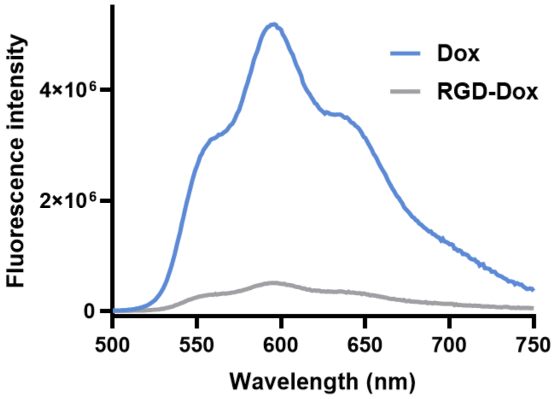

$$F_c = \frac{\text{Int.}_{\text{Dox}}}{\text{Int.}_{\text{RGD-Dox}}} = 10.2$$

Supplementary Fig. 65. **Calculation of the fluorescence coefficient (F_c)**. Fluorescence spectra of RGD-Dox (grey) and Dox (blue) at equal concentration (10 μM) in PBS (10% DMSO), indicating the F_c to be 10.2.

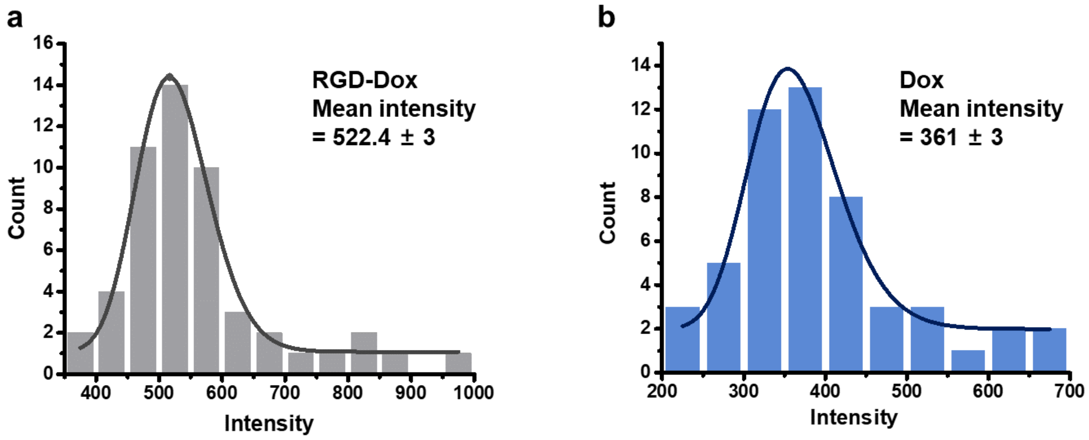

Supplementary Fig. 66. **Fluorescence quantitative analysis of B16F10 cells**. (a) With RGD-Dox or (b) With Dox. The results are shown as the mean ± standard deviation.

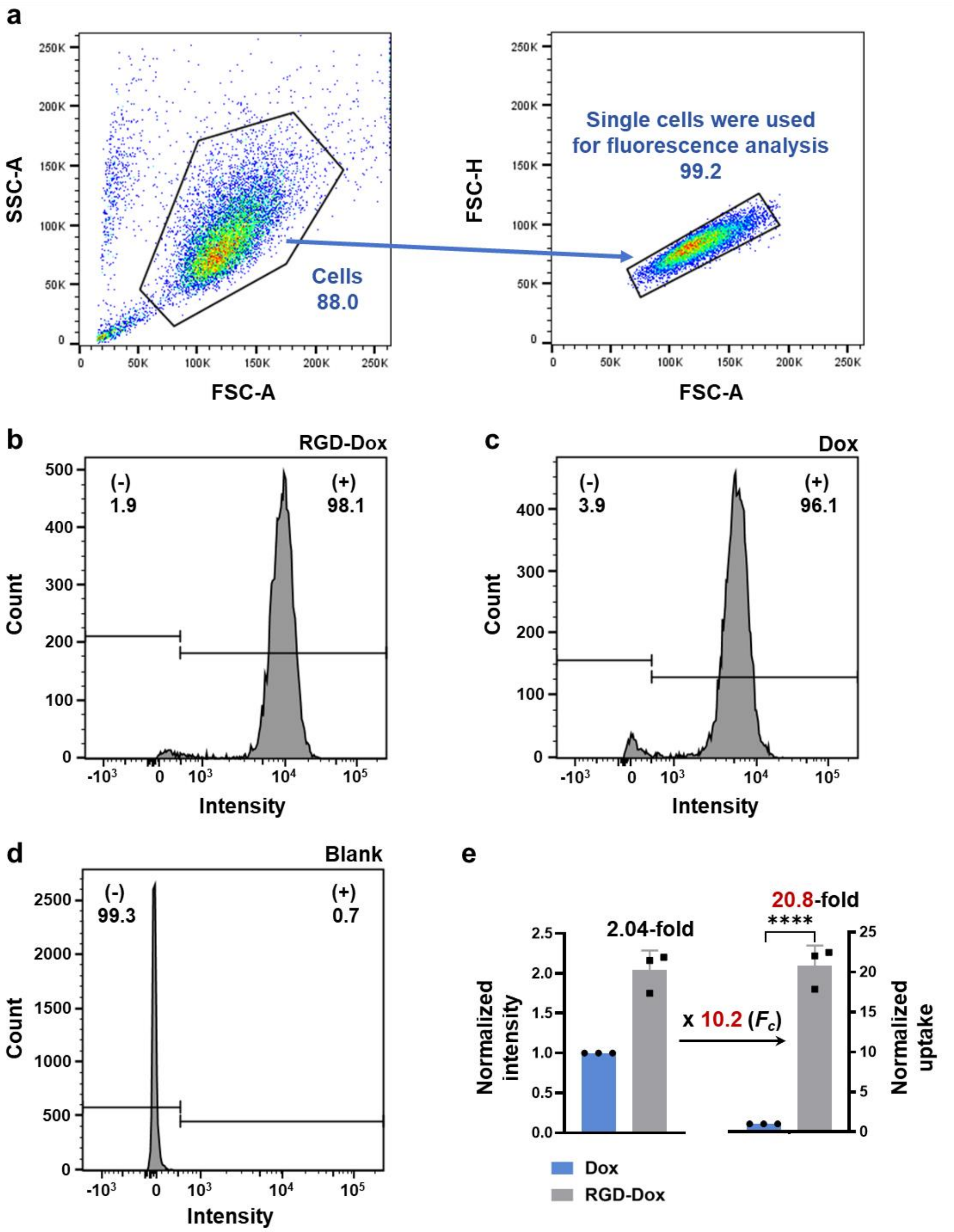

Supplementary Fig. 67. **Analysis of cell uptake through flow cytometry**. (a) Flow cytometry gating strategy. (b–d) B16F10 cells were incubated with various treatments (10 μM) at 37 °C for 2 h. (e) Quantification of fluorescence reveals the disparity in uptake between RGD-Dox and Dox by B16F10 cells. Data are presented as mean ± SD. (n = 3 biologically independent samples). Differences between two groups were assessed for significance using two-tailed unpaired Student's t-test, ****P < 0.0001. Source data are provided as a Source Data file.

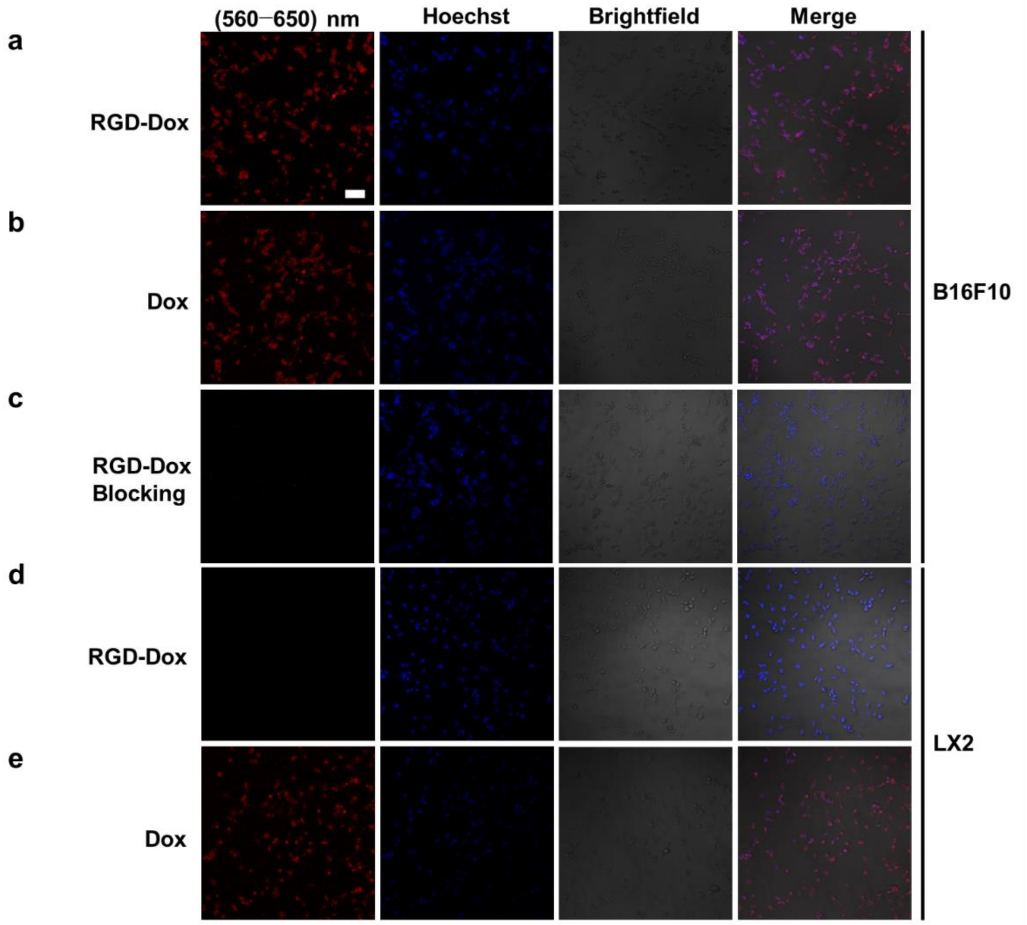

Supplementary Fig. 68. **Cell uptake analysis by fluorescence imaging**. Fluorescence imaging of B16F10 cells with (a) RGD-Dox or (b) Dox, and (c) pre-treated with RGD, then with RGD-Dox. Fluorescence imaging of LX2 cells with (d) RGD-Dox or (e) Dox. Scale bar: 100 μm.

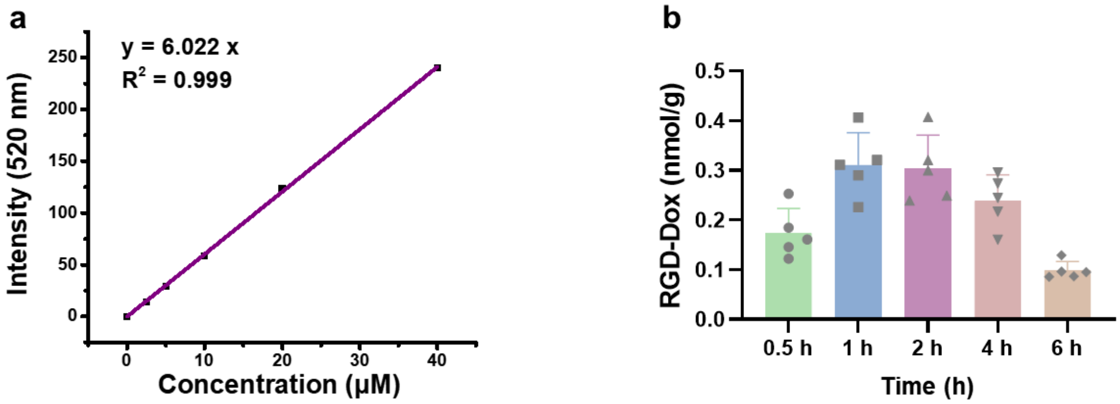

Supplementary Fig. 69. **The accumulation of RGD-Dox at various time points in tumor tissues**. (a) The standard absorption curve of RGD-Dox at various concentrations. (20 μL of sample was injected.) (b) The average concentration of RGD-Dox (3.67 μmol·kg^{-1}) in tumor after *i.v.* injection at various time intervals. Data are presented as mean ± SD. (n = 5 biologically independent samples). Source data are provided as a Source Data file.

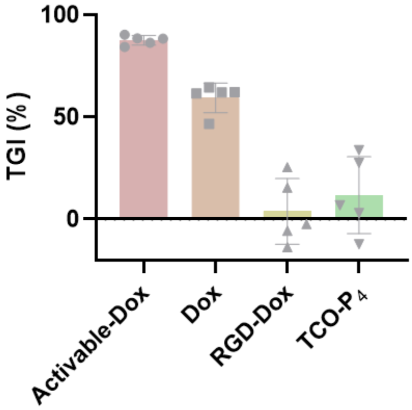

Group	TGI (%)
Activable-Dox	87.6 ± 2.6
Dox	59.4 ± 1.3
RGD-Dox	3.8 ± 14.7
$TCO-P_4$	11.7 ± 20.9

Supplementary Fig. 70. **TGI calculations for the four treatment groups**. Data are presented as mean ± SD. (n = 5 biologically independent samples). Source data are provided as a Source Data file.

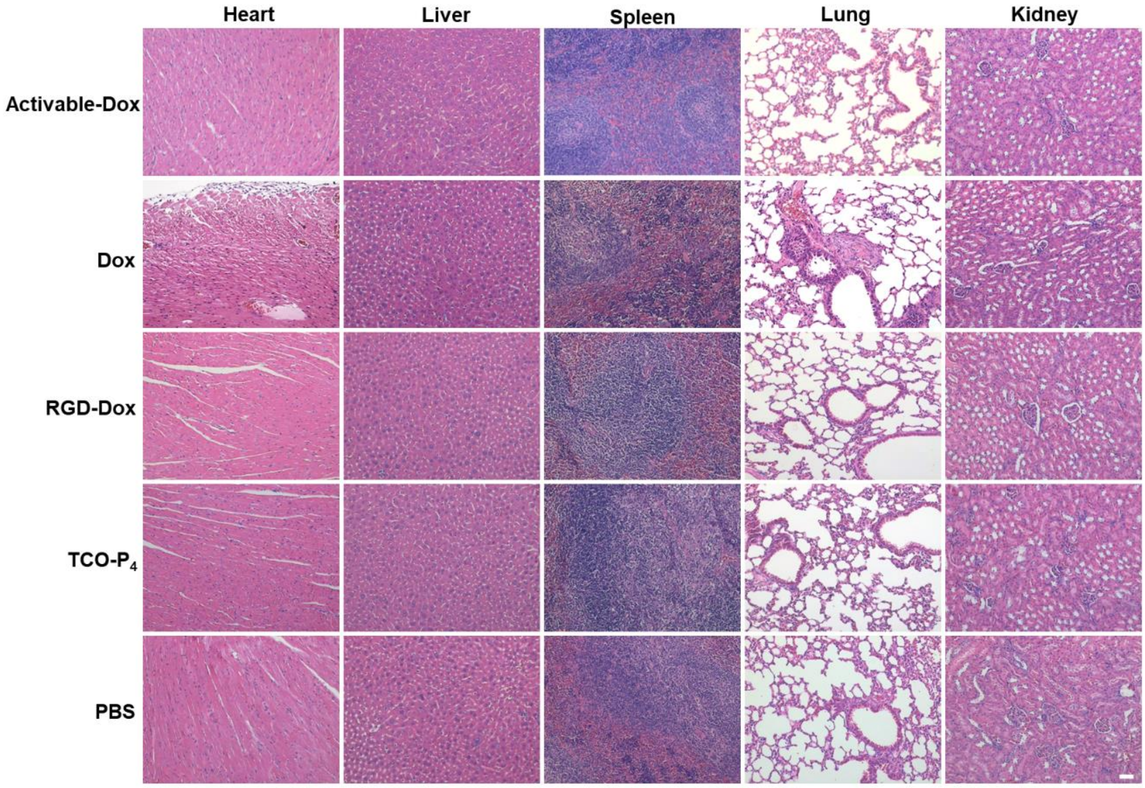

Supplementary Fig. 71. **Histopathology analysis**. Histological examination of heart, liver, spleen, lung, and kidney. Scale bar, 50 μm.

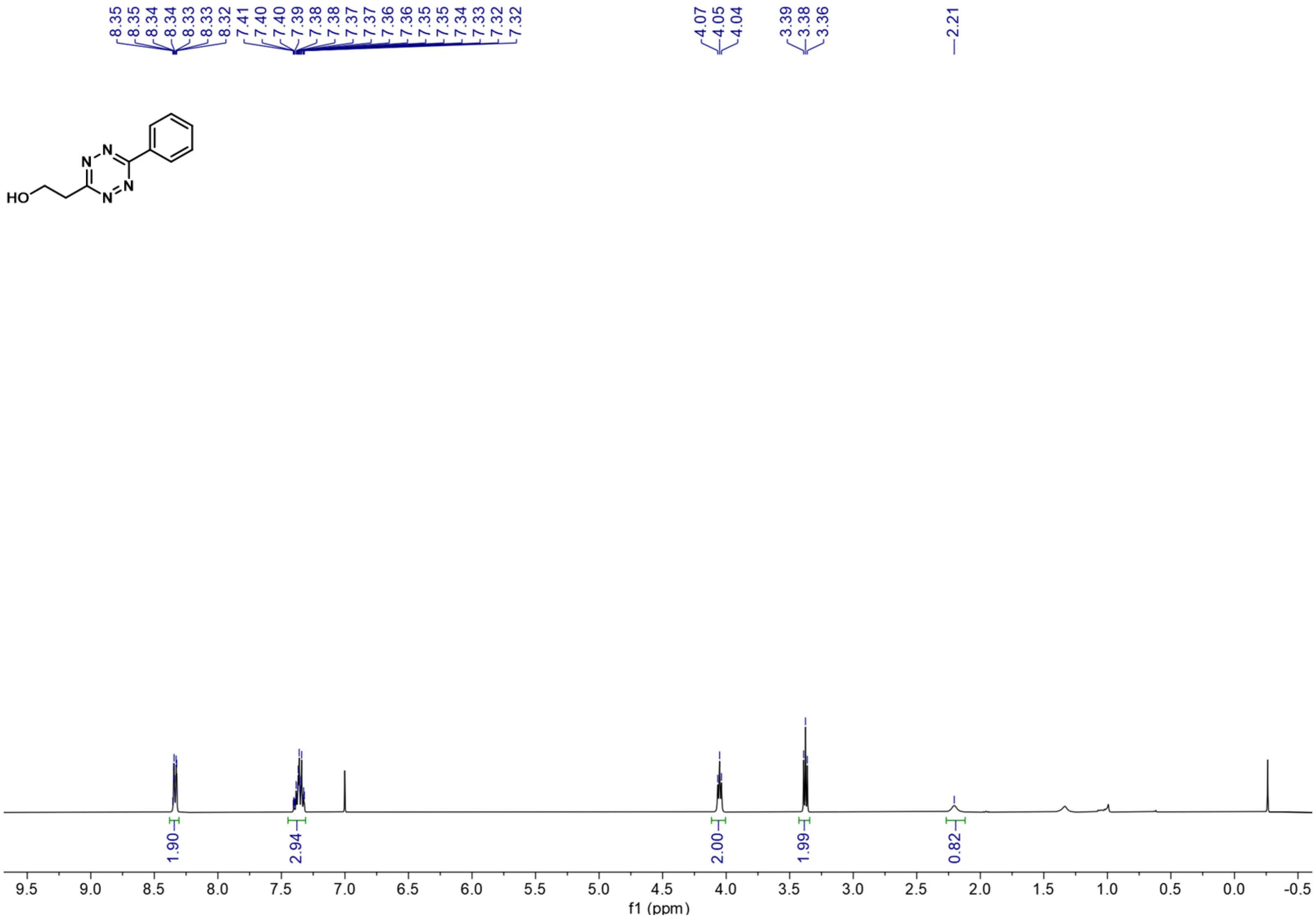

Supplementary Fig. 72. **^{1}H NMR spectra of compound S1**.

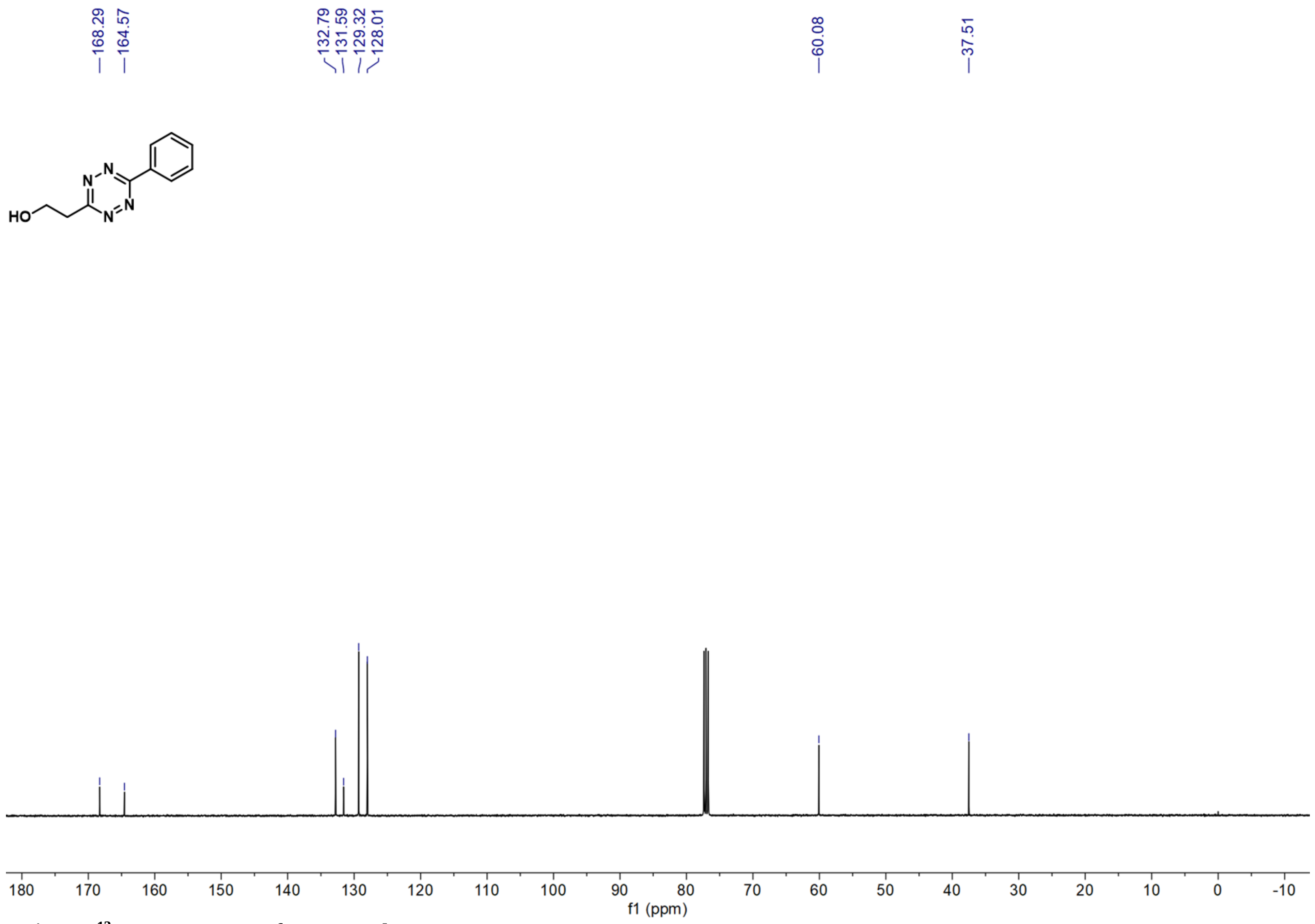

Supplementary Fig. 73. ^{13}**C NMR spectra of compound S1**.

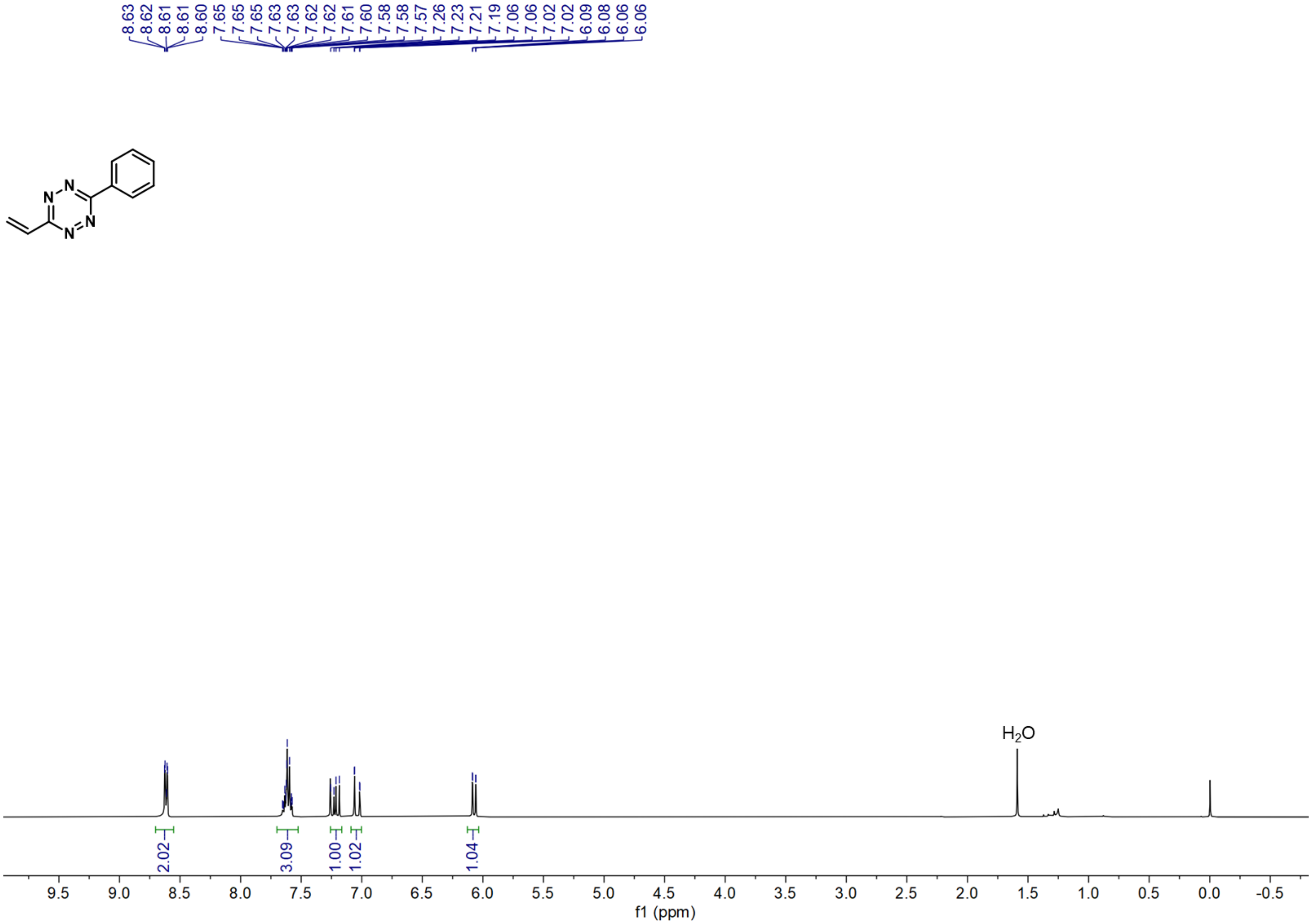

Supplementary Fig. 74. ^{1}H NMR spectra of compound 1.

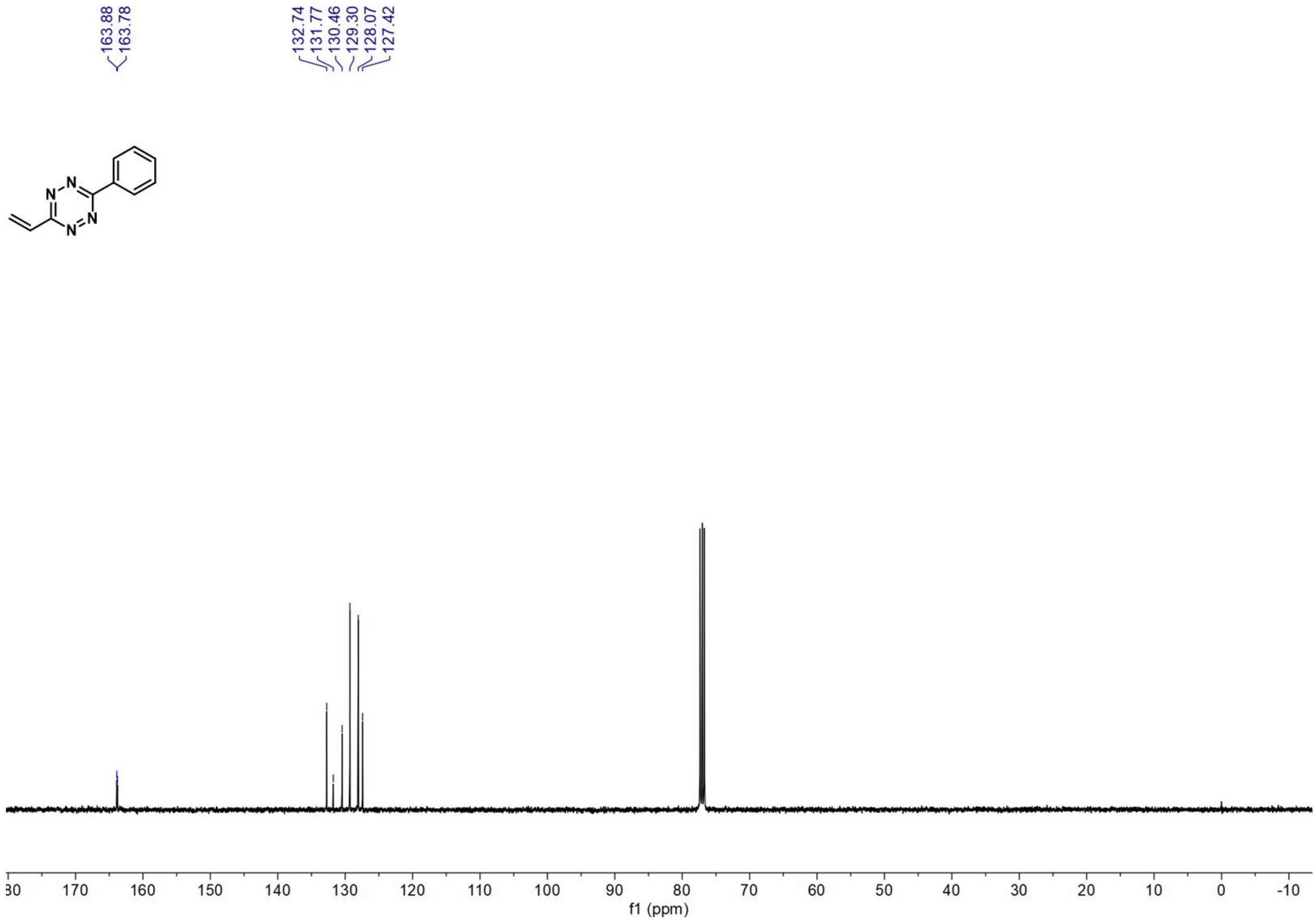

Supplementary Fig. 75. **^{13}C NMR spectra of compound 1**.

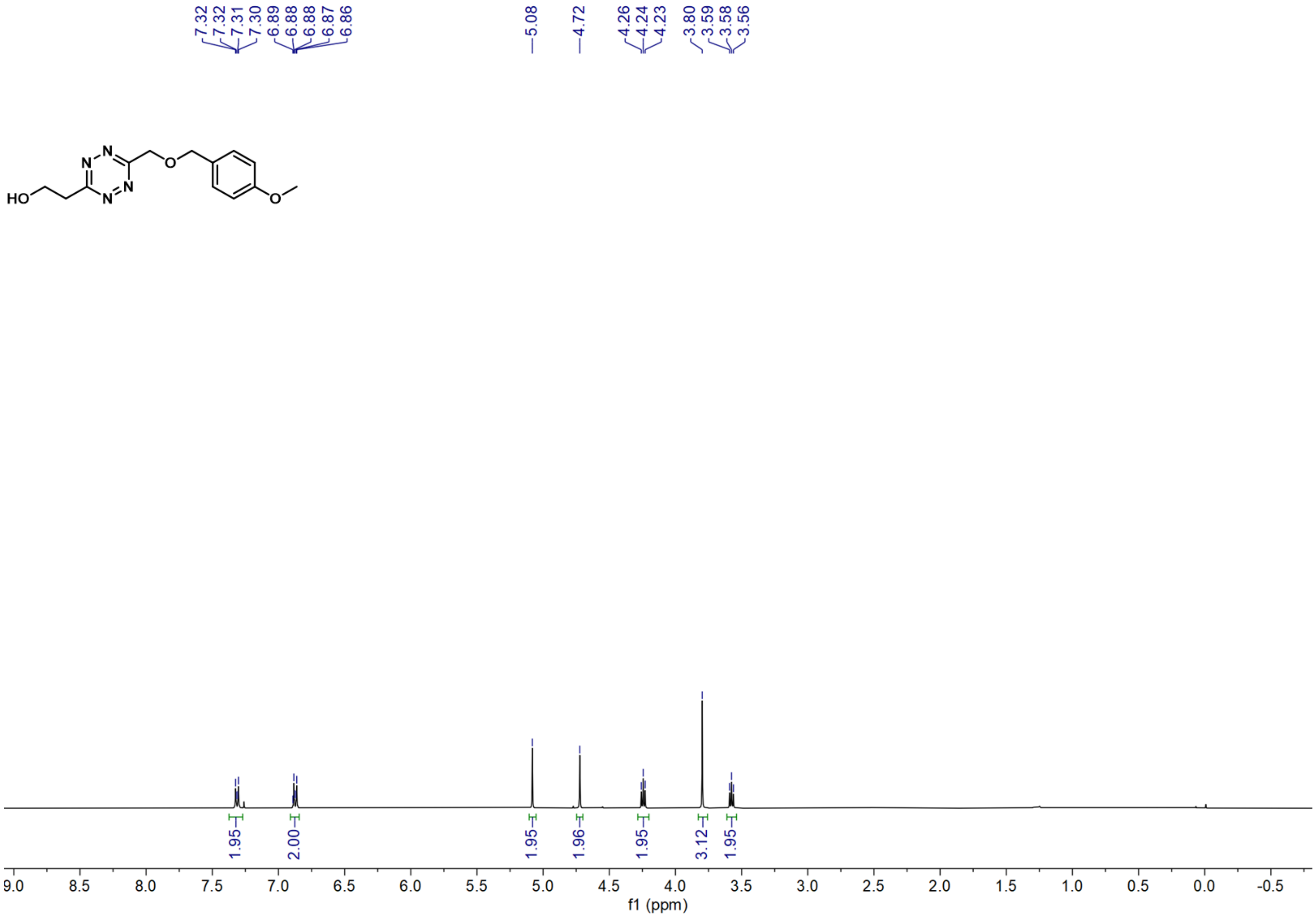

Supplementary Fig. 76. **^{1}H NMR spectra of compound S2a**.

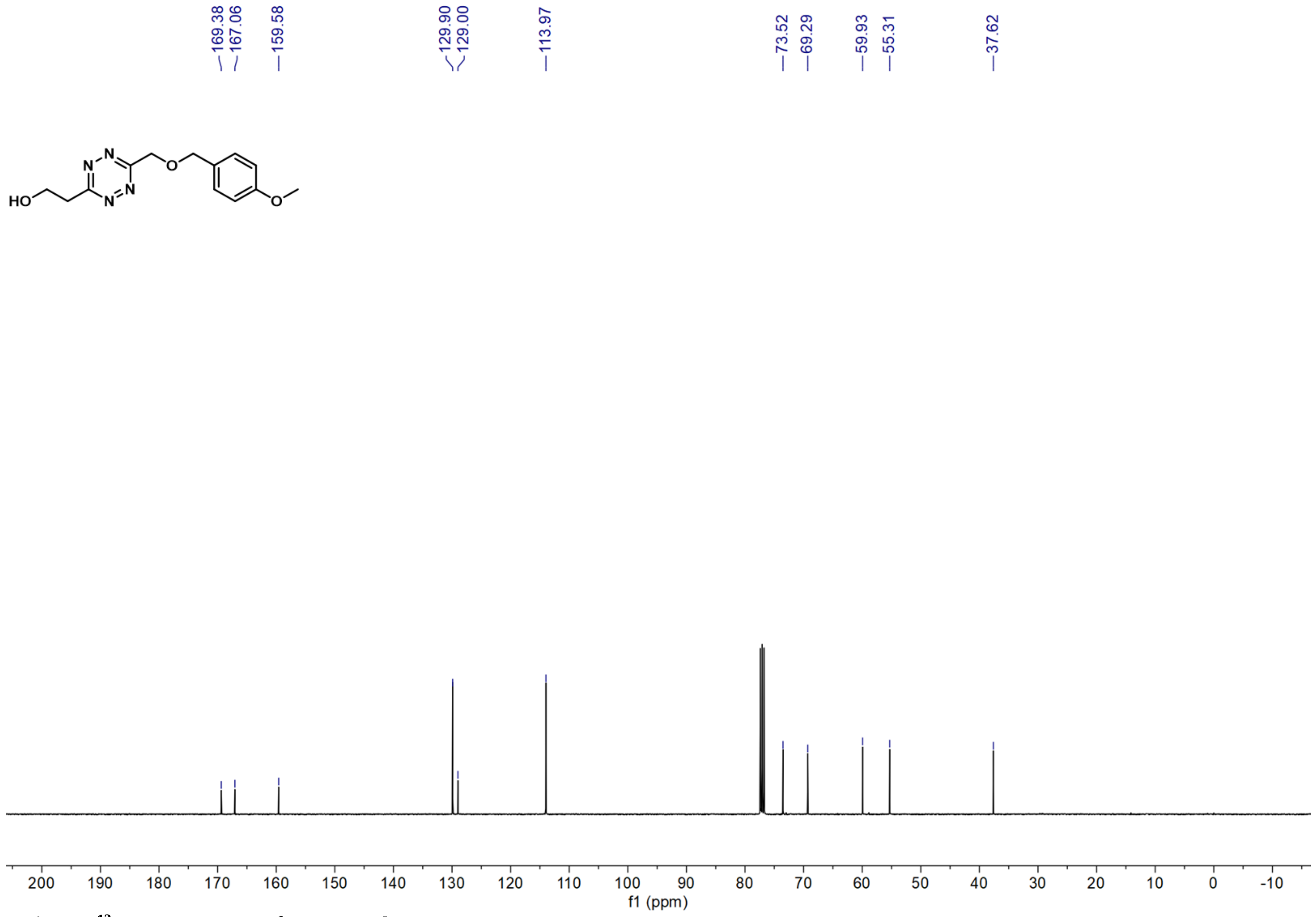

Supplementary Fig. 77. **^{13}C NMR spectra of compound S2a**.

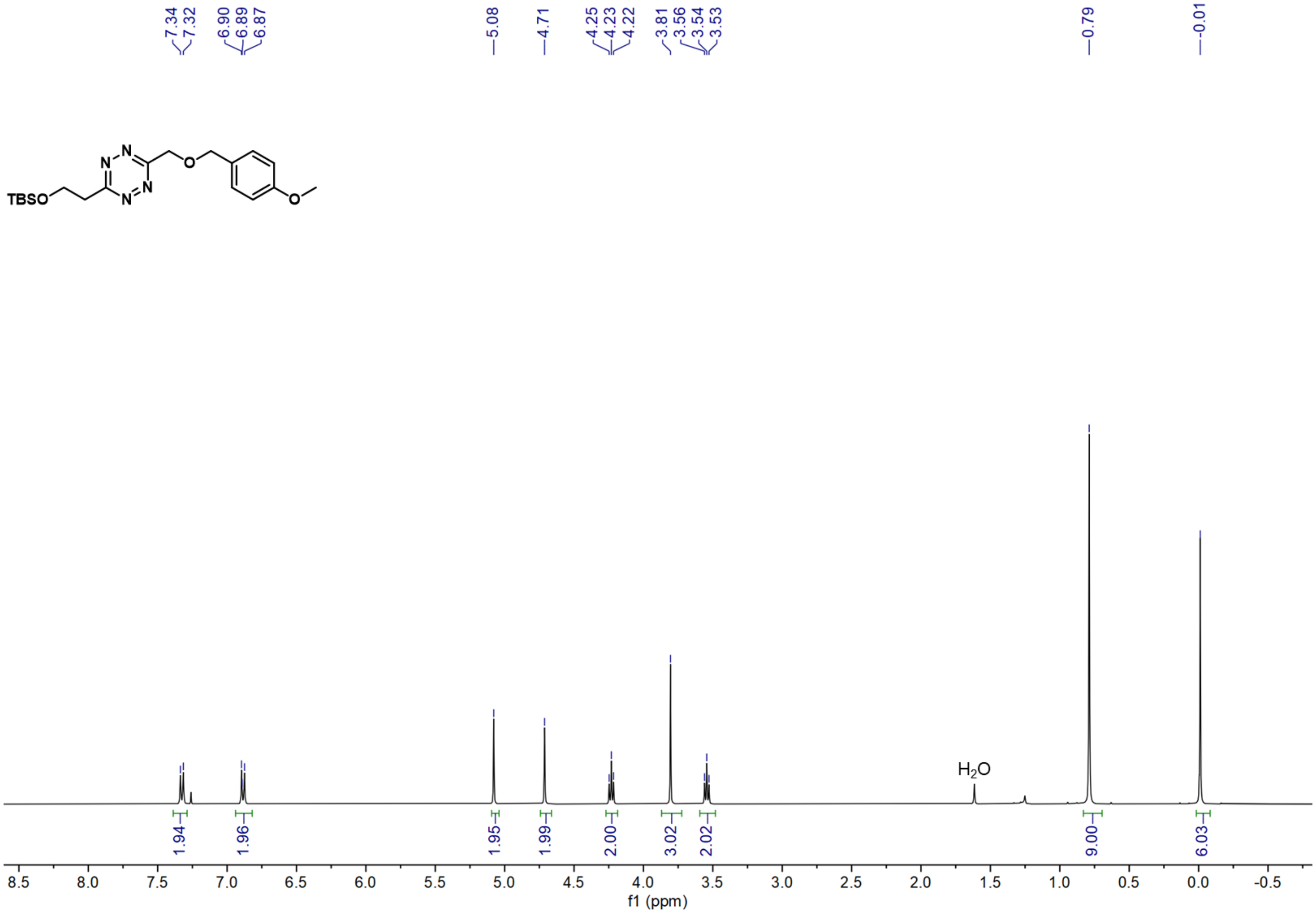

Supplementary Fig. 78. **^{1}H NMR spectra of compound S2b**.

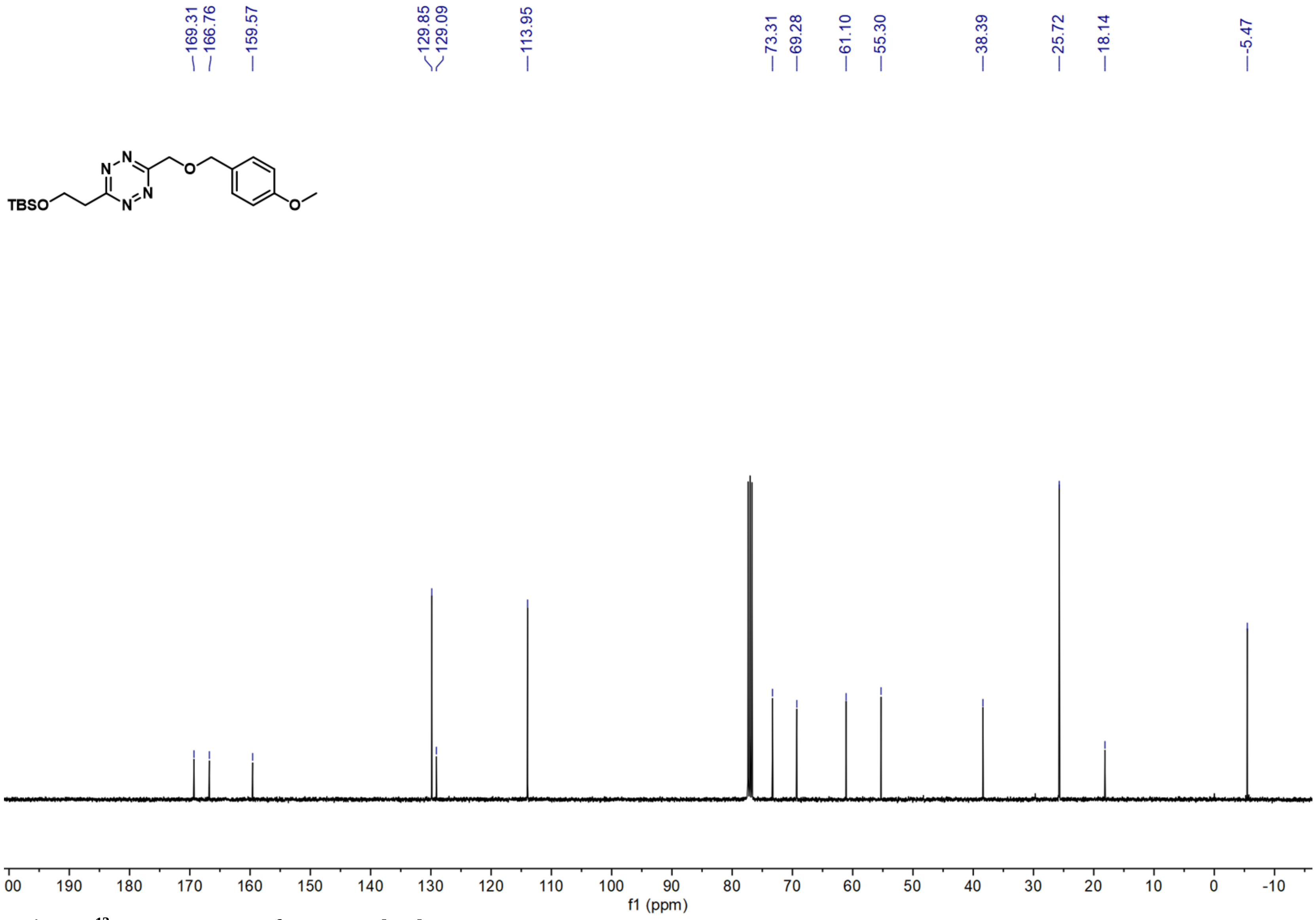

Supplementary Fig. 79. ^{13}C **NMR spectra of compound S2b**.

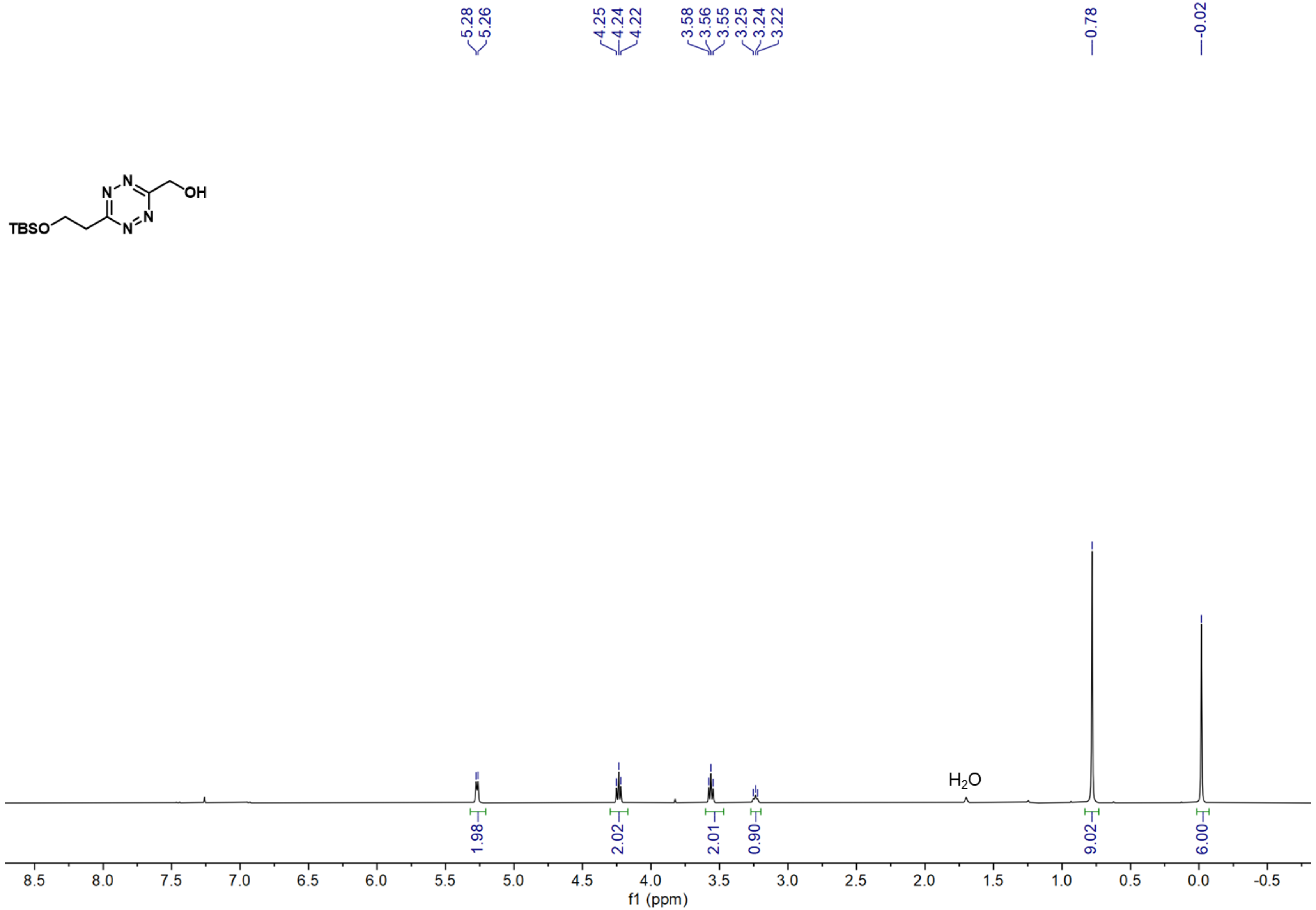

Supplementary Fig. 80. **^{1}H NMR spectra of compound 2**.

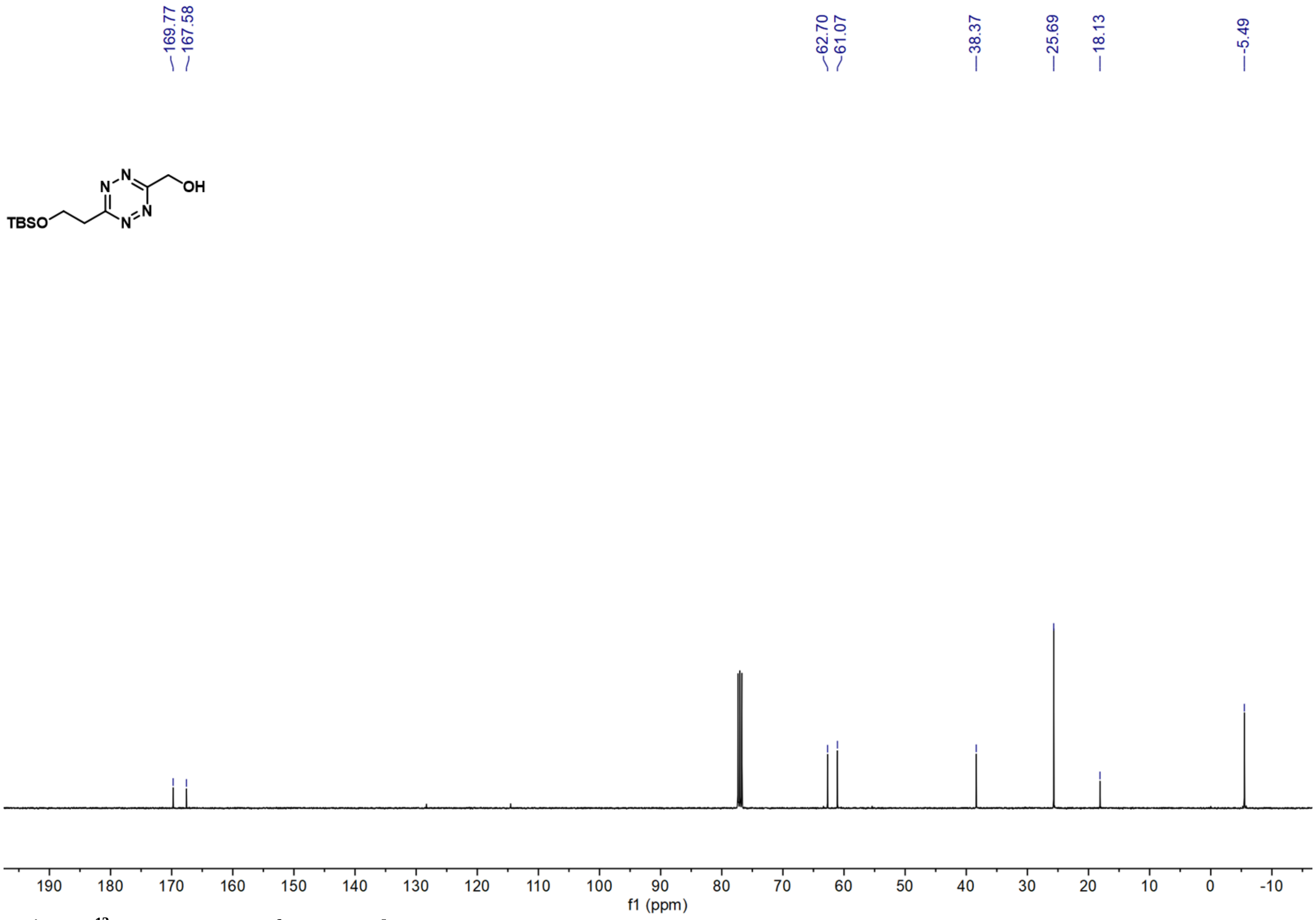

Supplementary Fig. 81. **^{13}C NMR spectra of compound 2**.

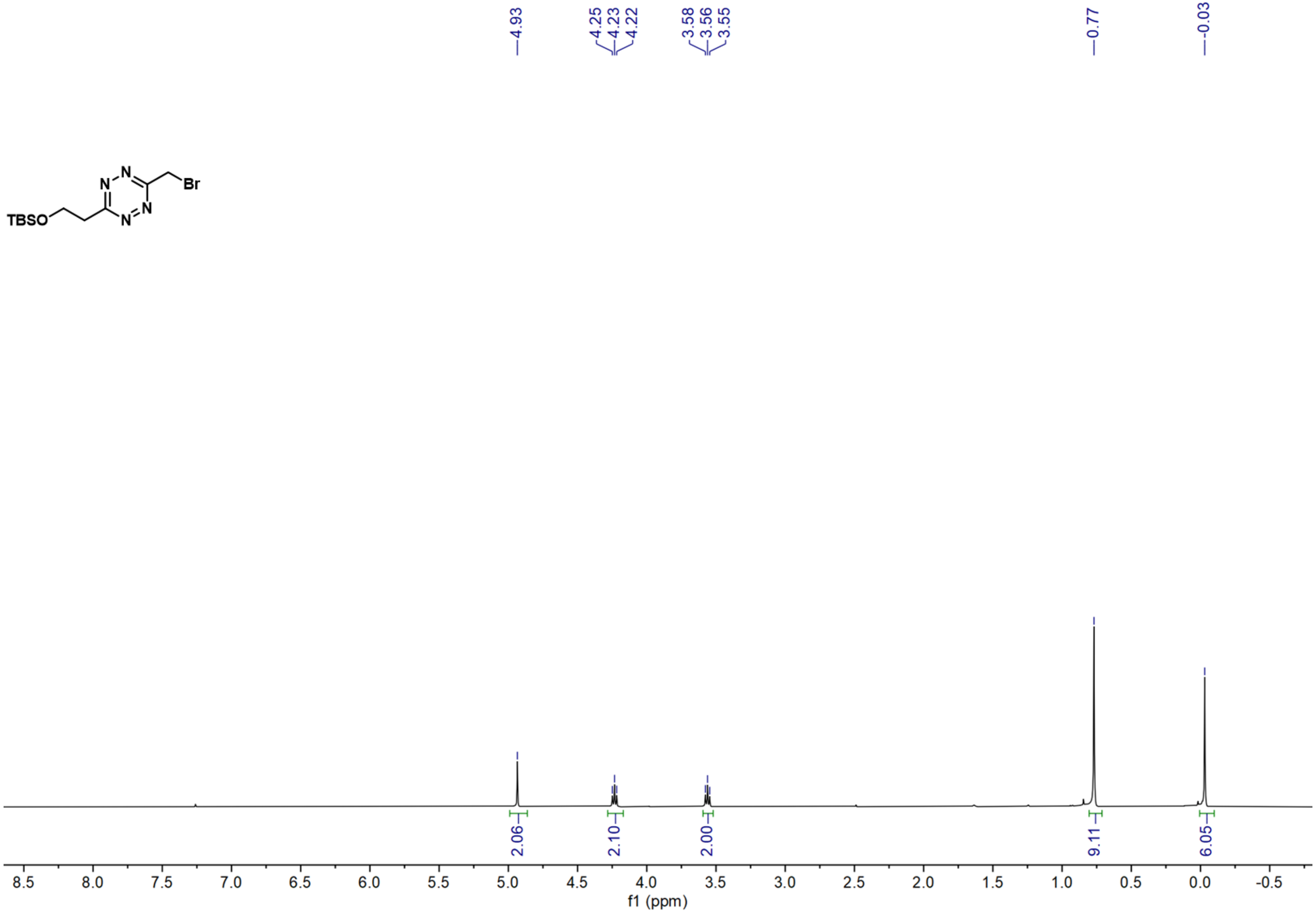

Supplementary Fig. 82. **^{1}H NMR spectra of compound S3**.

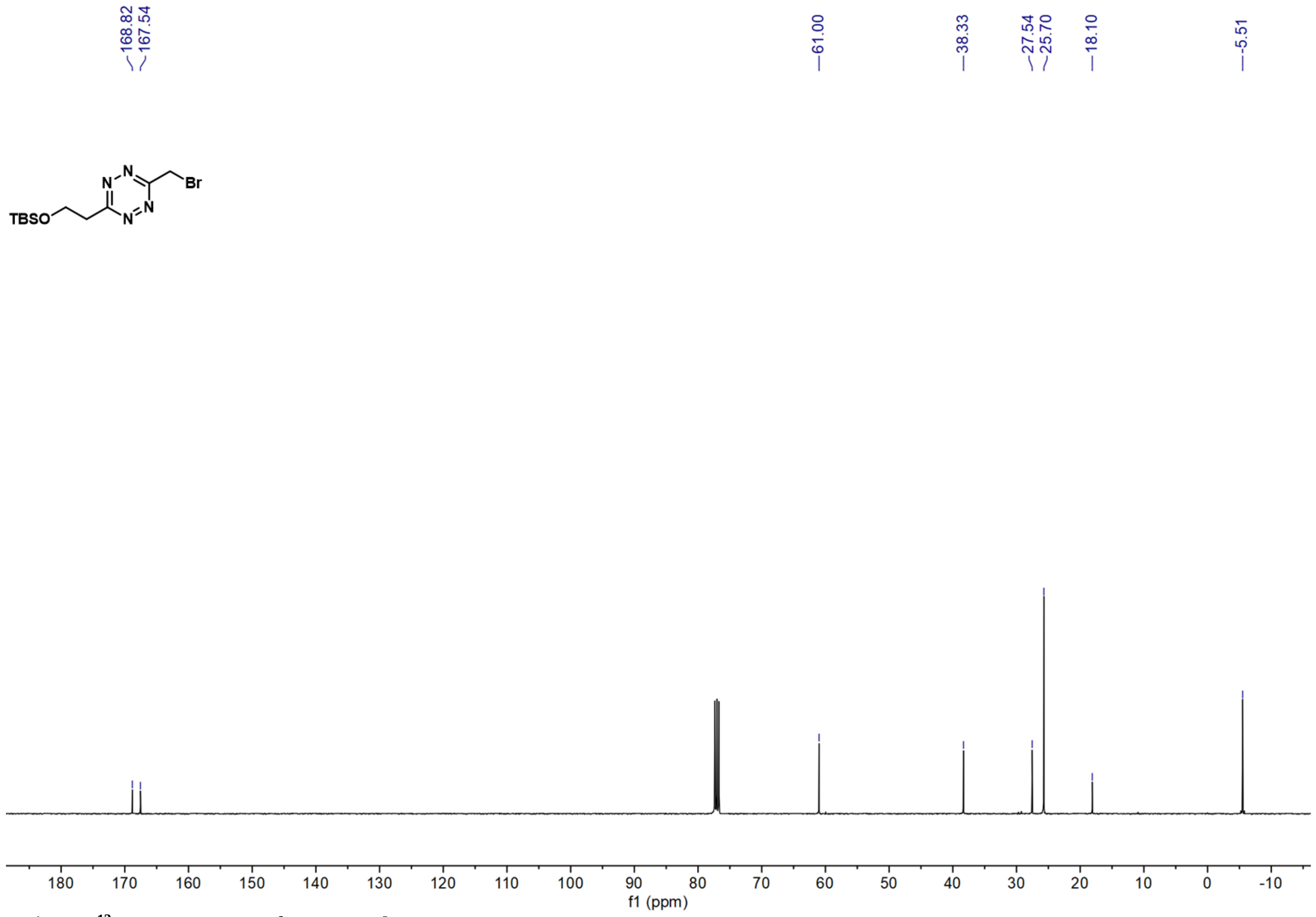

Supplementary Fig. 83. ^{13}C NMR spectra of compound S3.

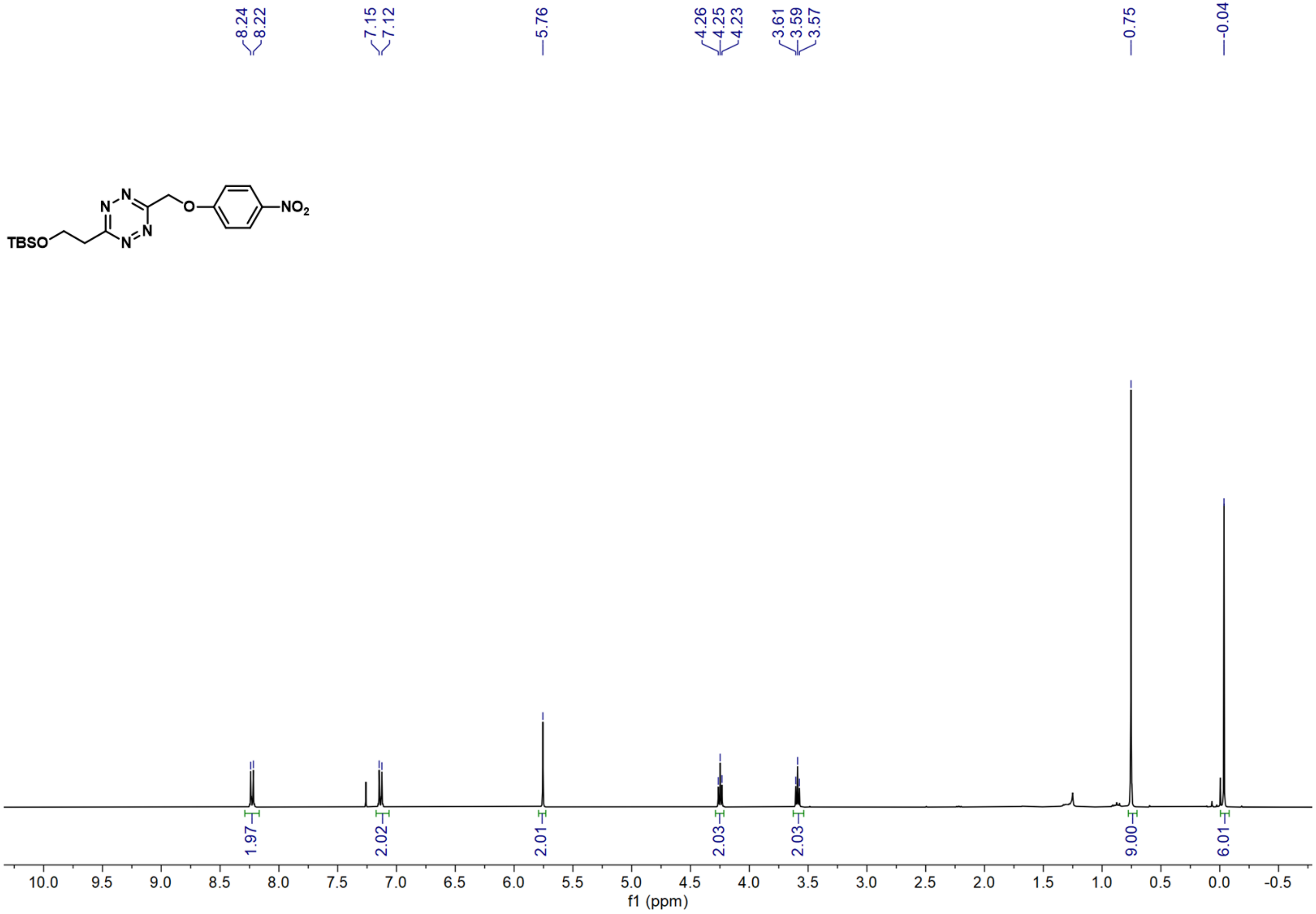

Supplementary Fig. 84. ^{1}H NMR spectra of compound 3a.

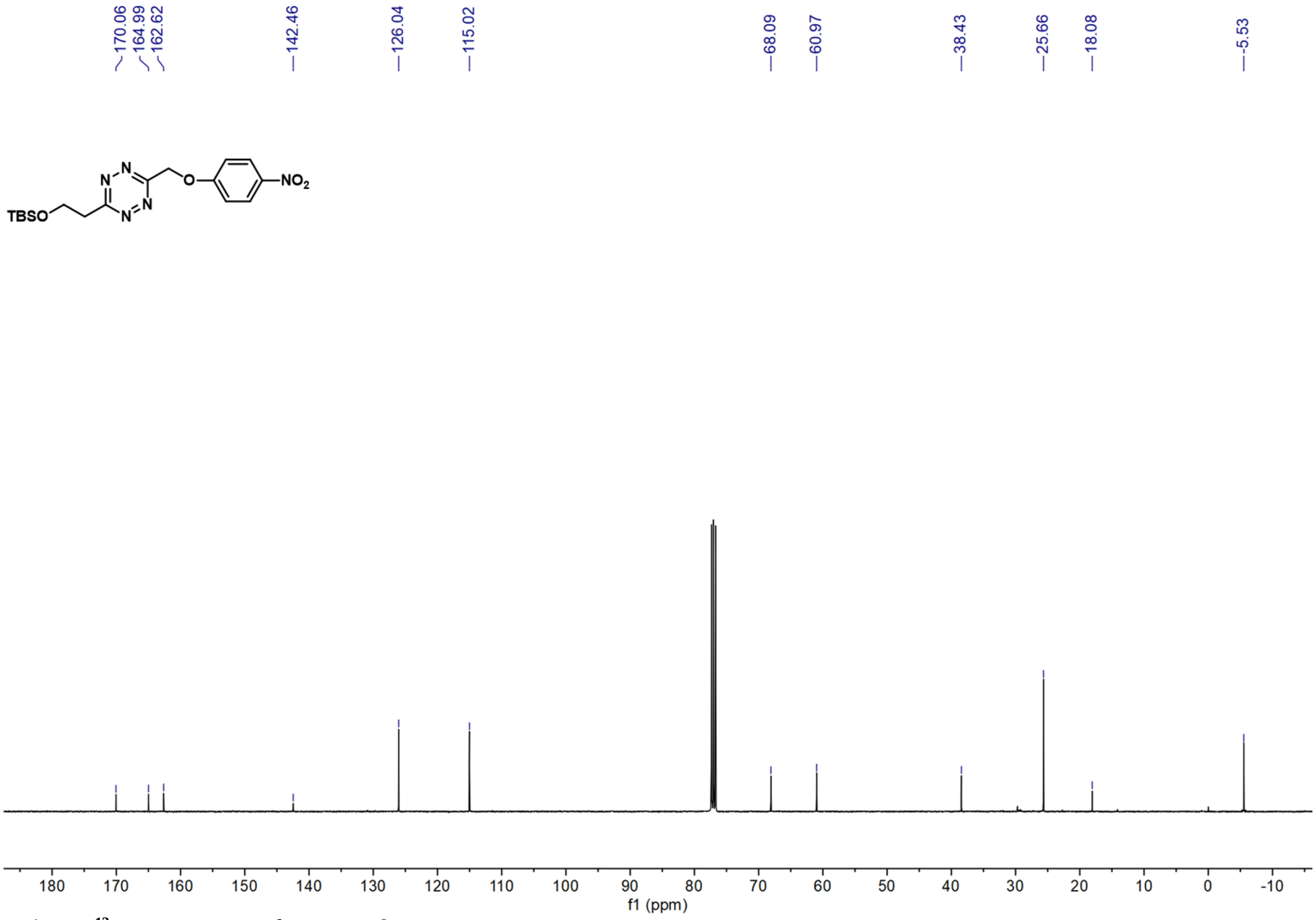

Supplementary Fig. 85. ^{13}C NMR spectra of compound 3a.

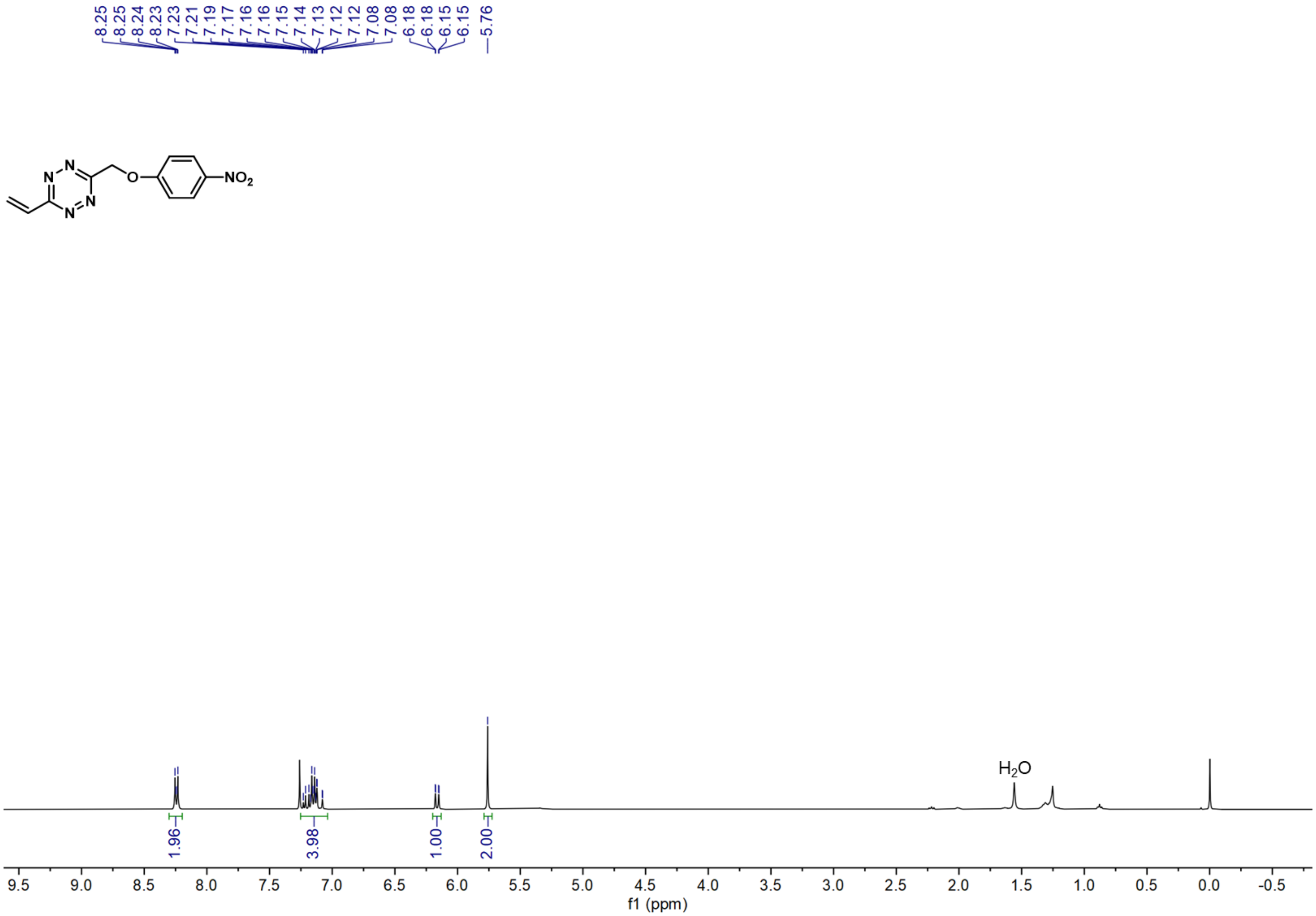

Supplementary Fig. 86. ^{1}H NMR spectra of compound 3.

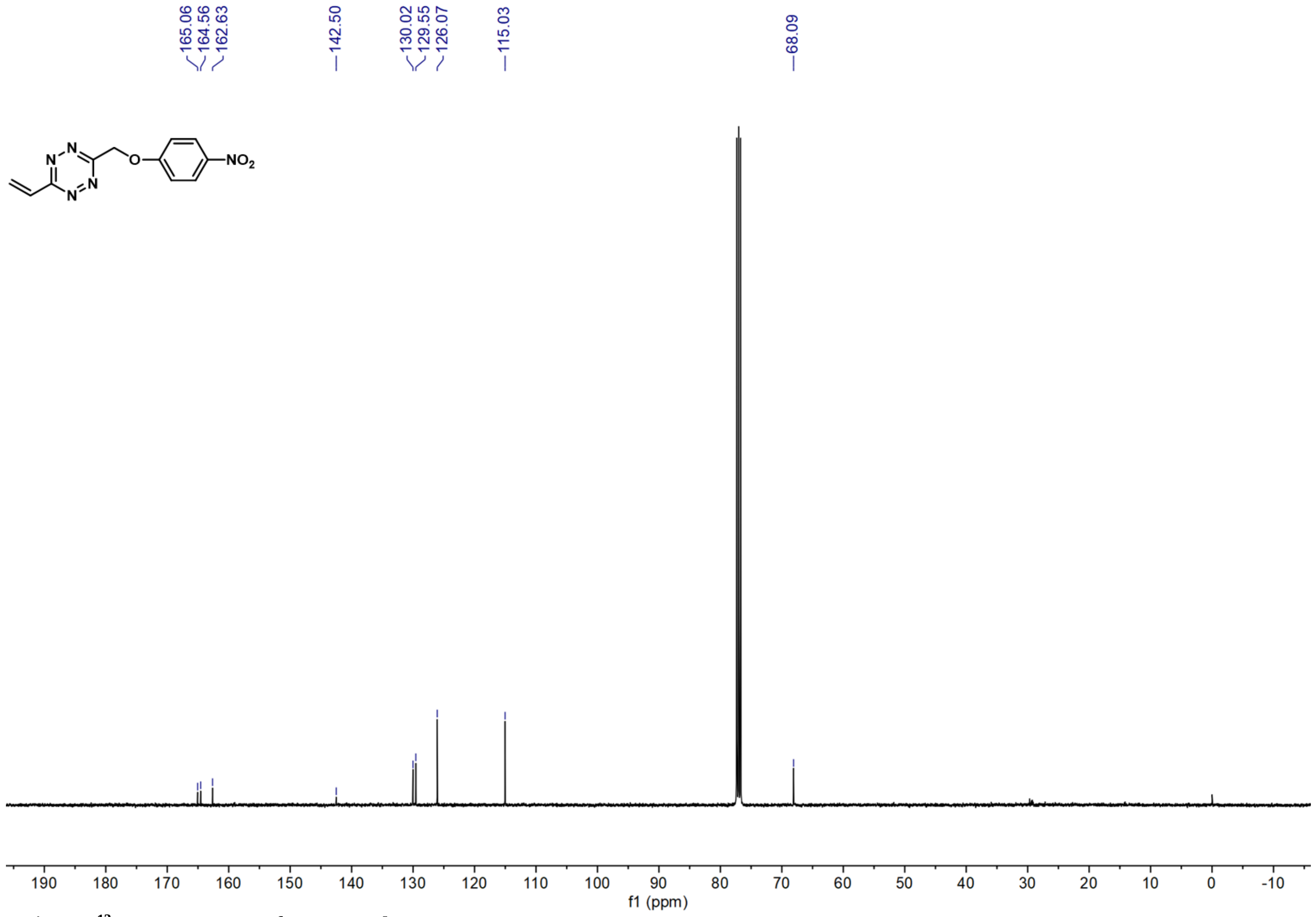

Supplementary Fig. 87. **^{13}C NMR spectra of compound 3**.

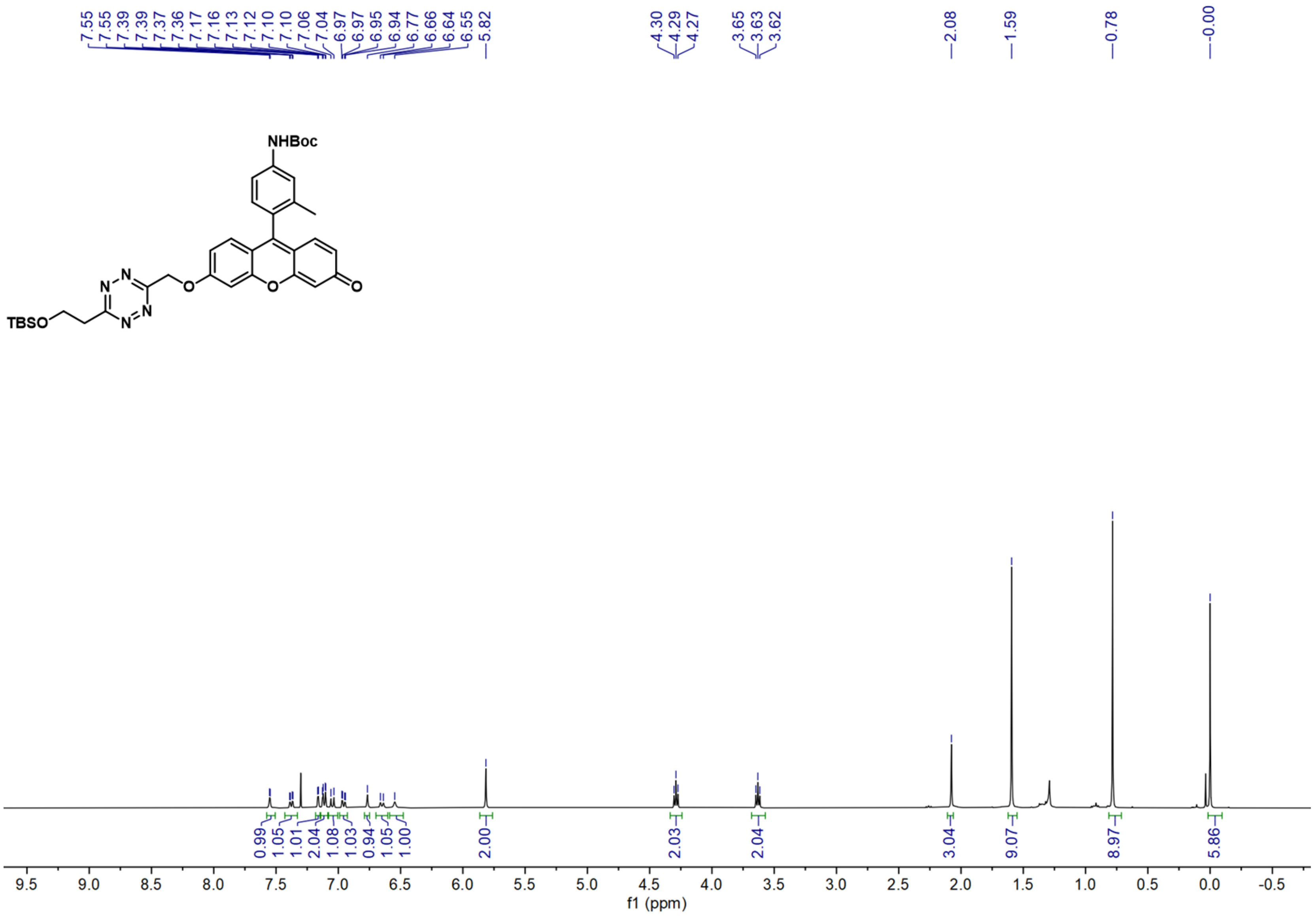

Supplementary Fig. 88. ^{1}H NMR spectra of compound 4a.

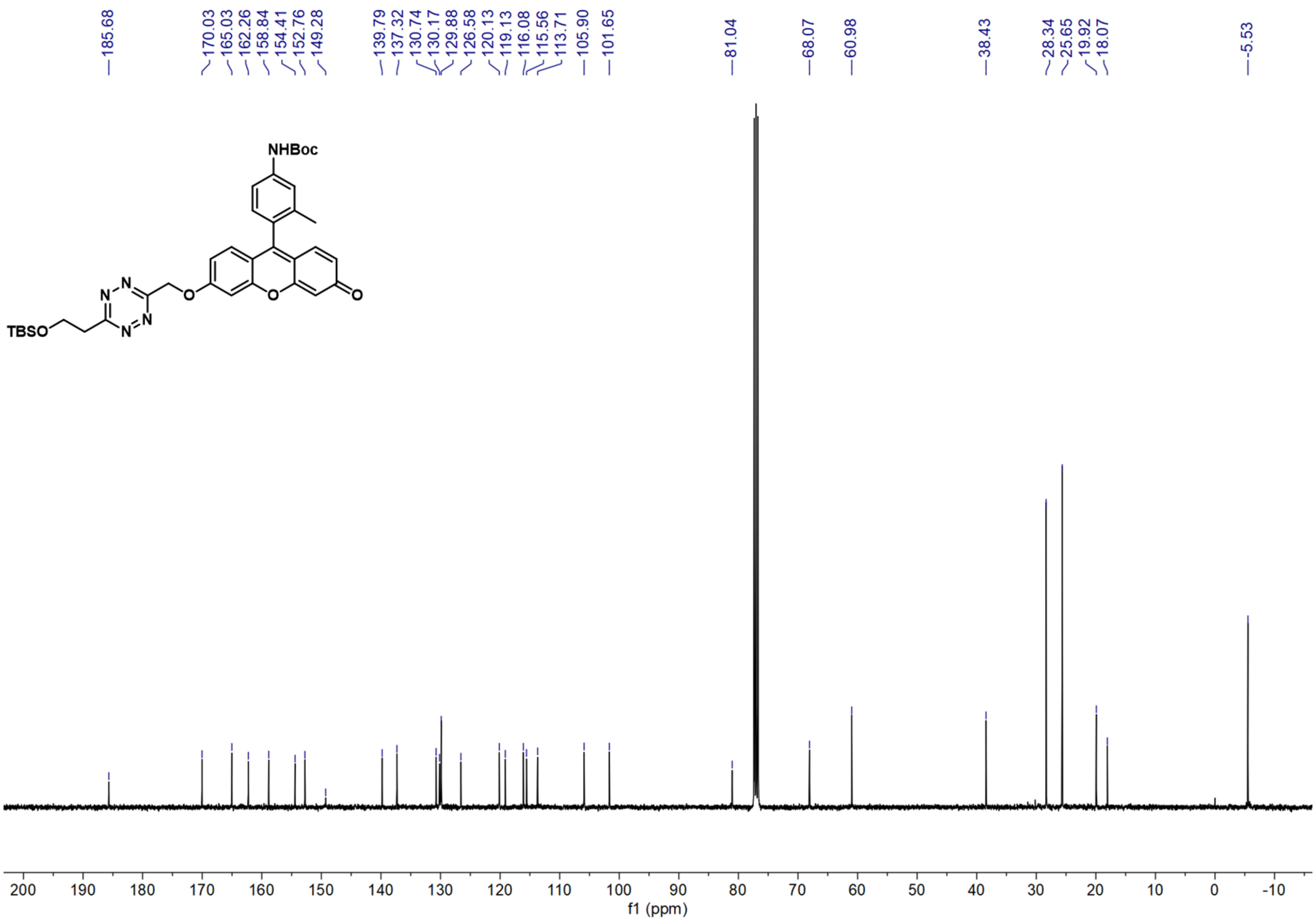

Supplementary Fig. 89. ^{13}**C NMR spectra of compound 4a**.

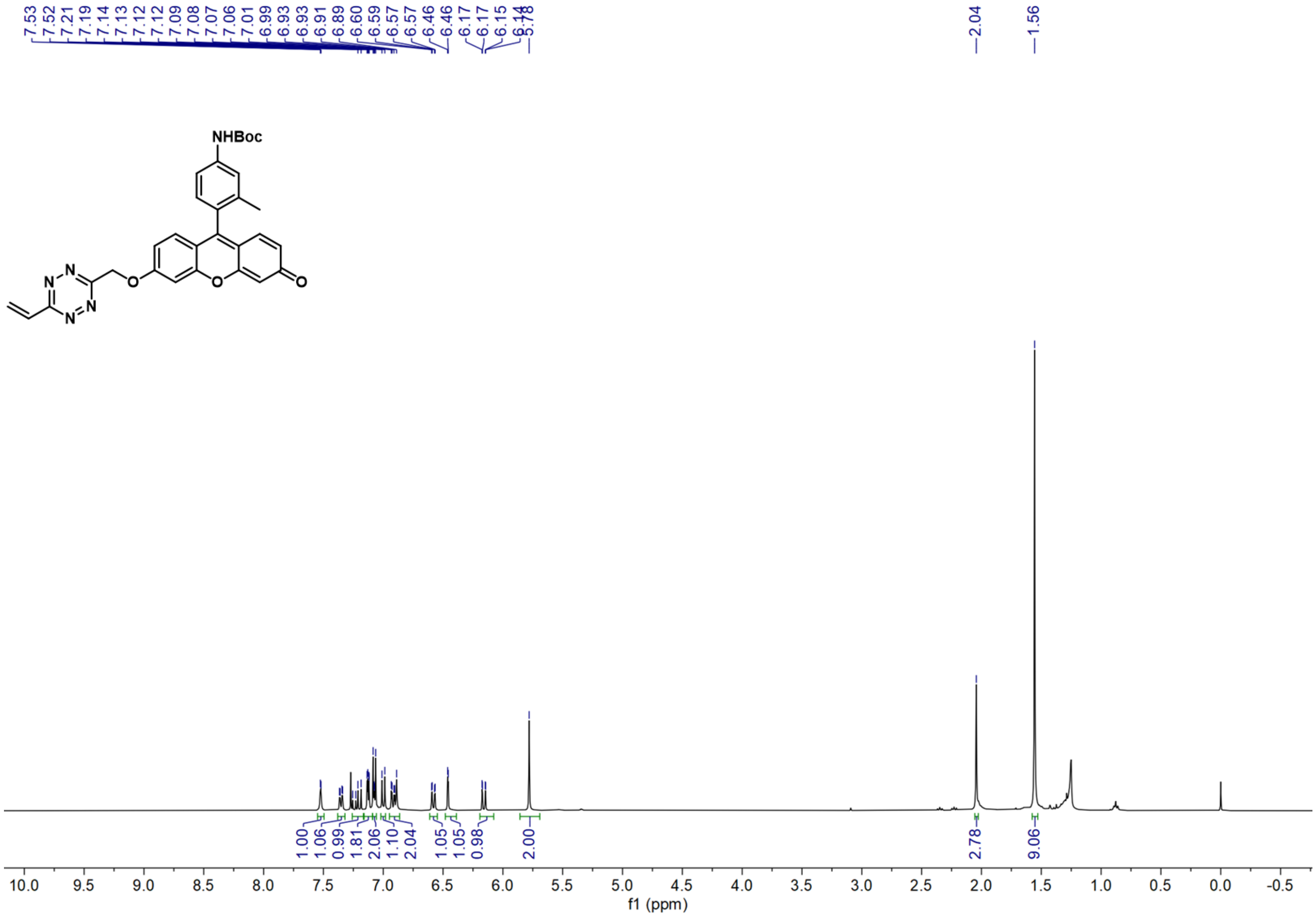

Supplementary Fig. 90. ^{1}H NMR spectra of compound 4.

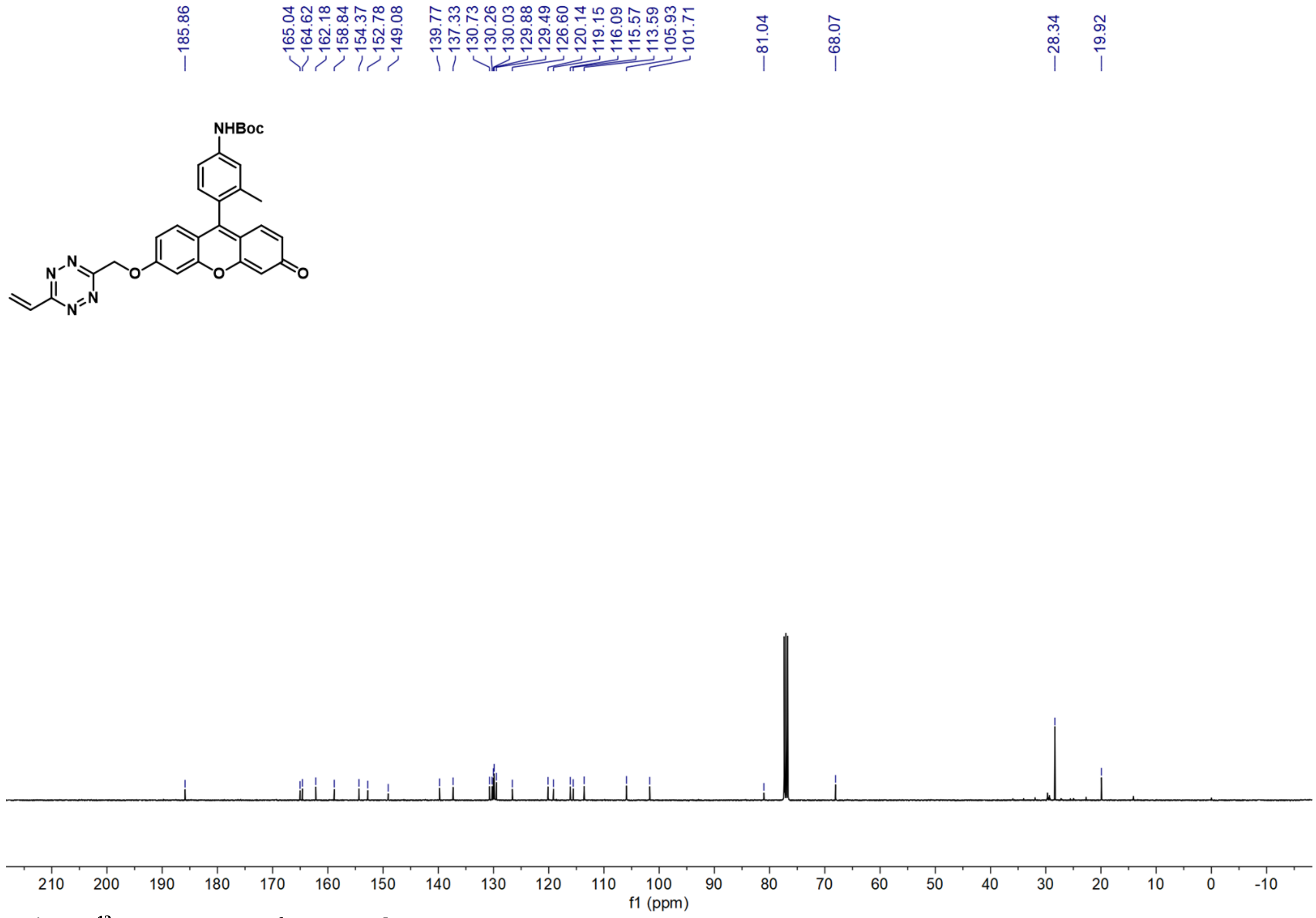

Supplementary Fig. 91. ^{13}C NMR spectra of compound 4.

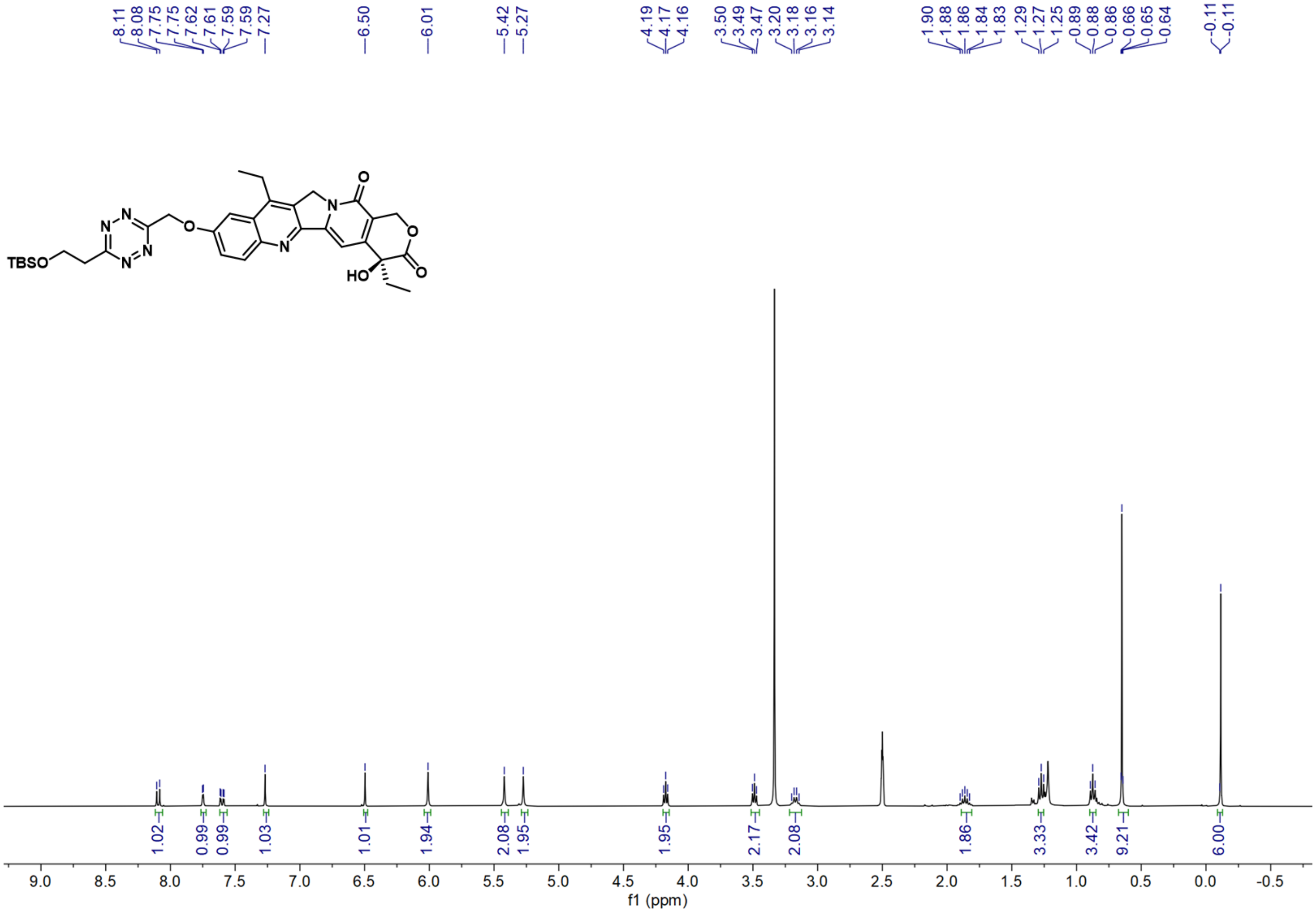

Supplementary Fig. 92. ^{1}H NMR spectra of compound 5a.

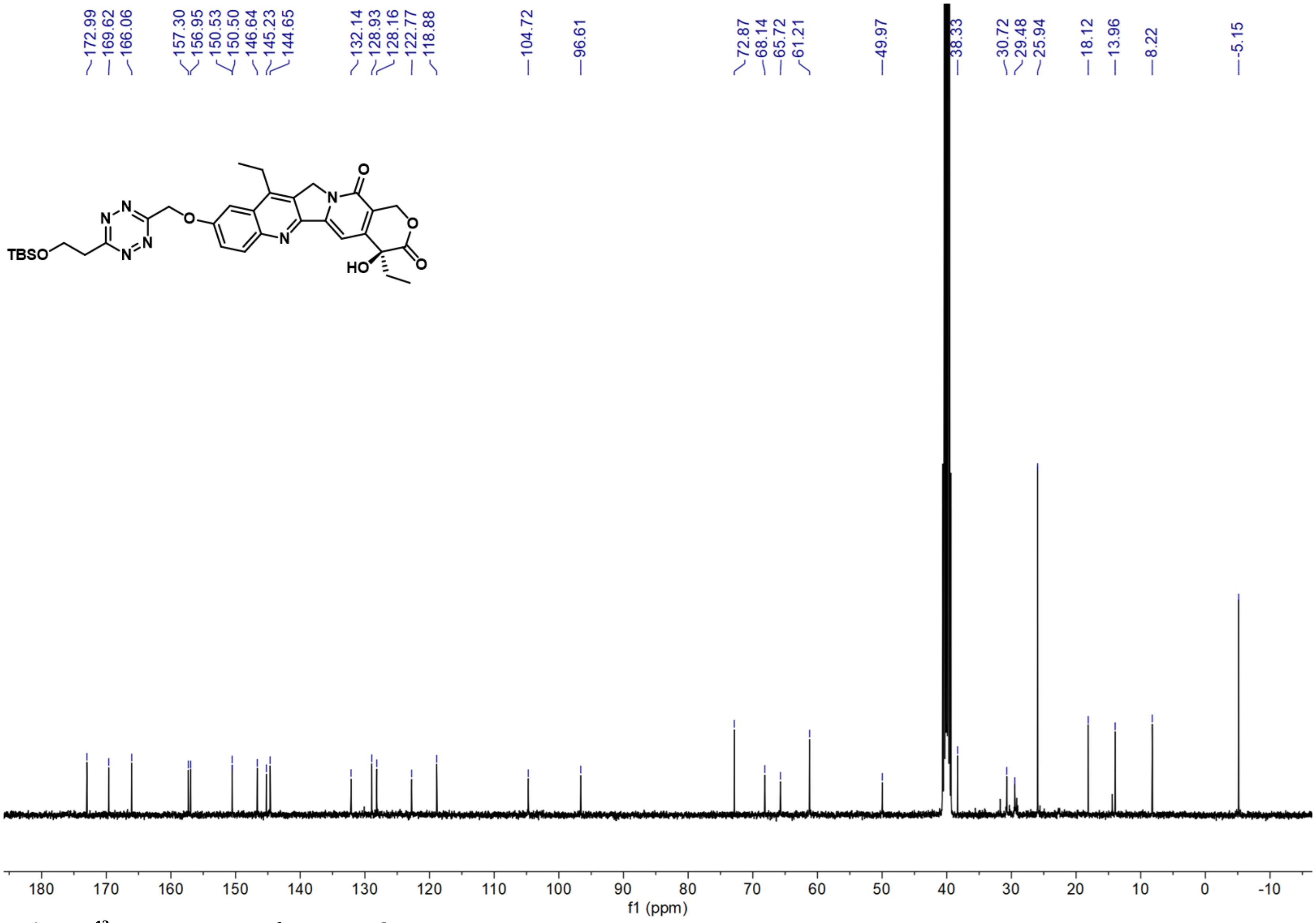

Supplementary Fig. 93. ^{13}**C NMR spectra of compound 5a**.

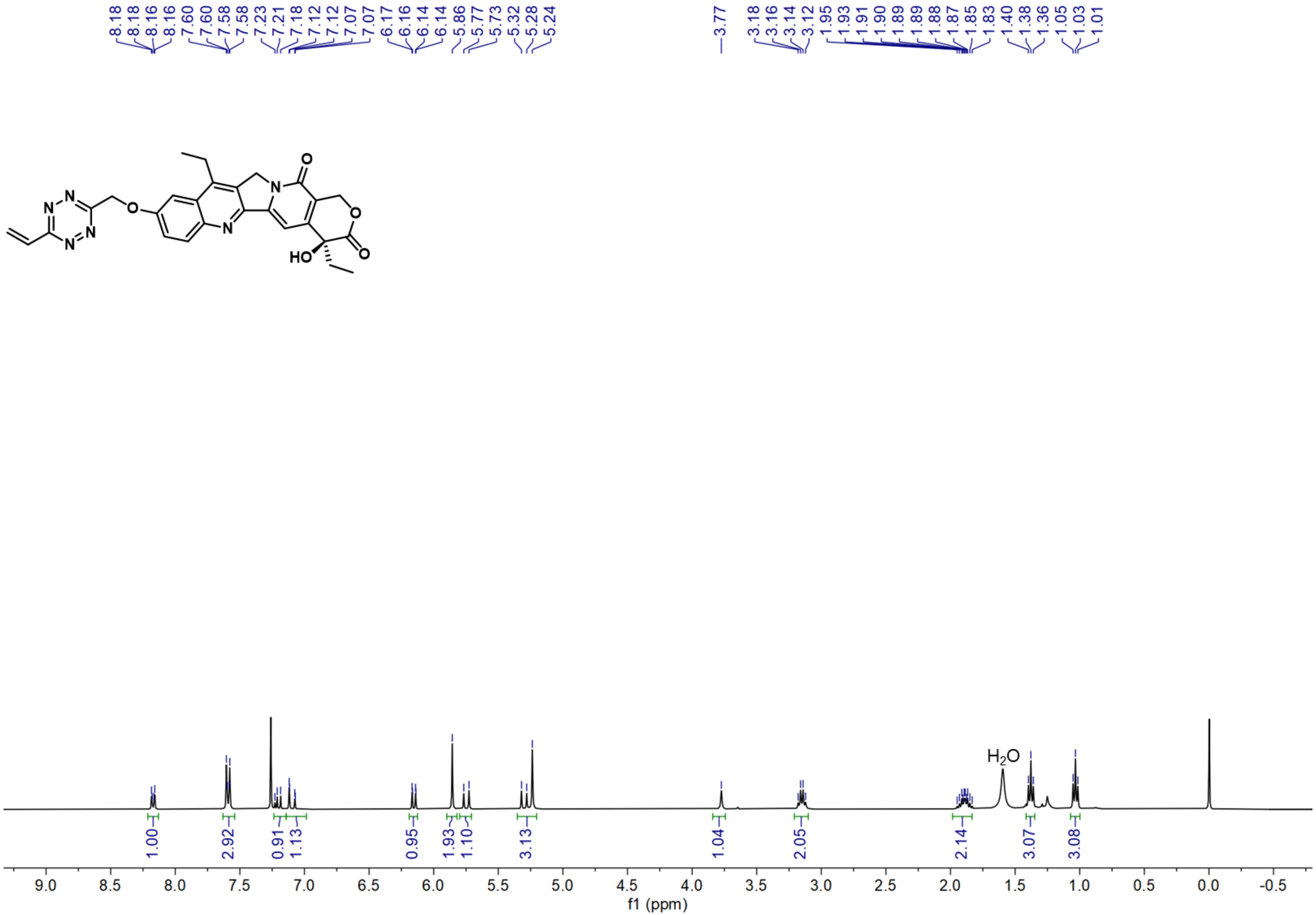

Supplementary Fig. 94. **^{1}H NMR spectra of compound 5**.

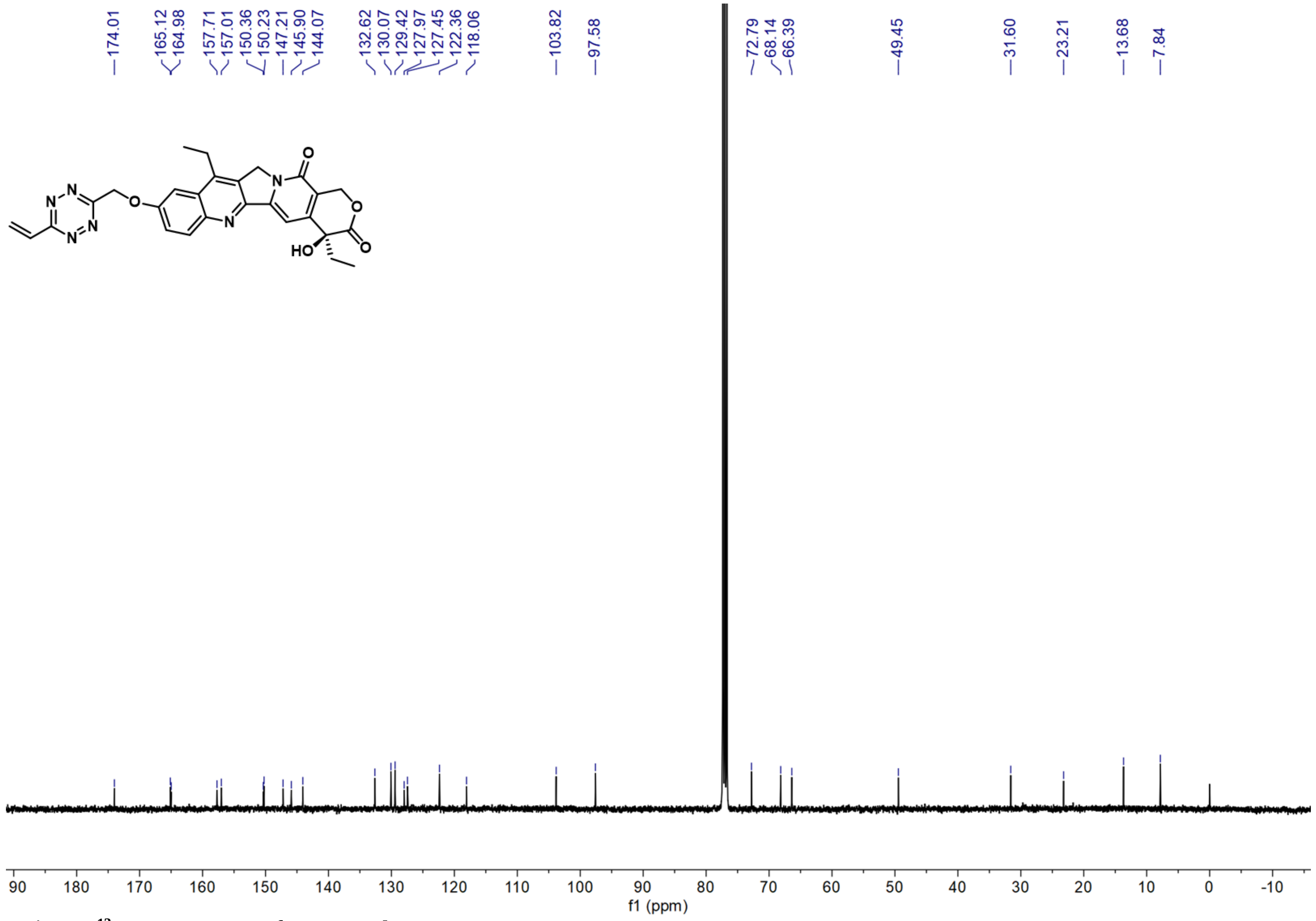

Supplementary Fig. 95. **^{13}C NMR spectra of compound 5**.

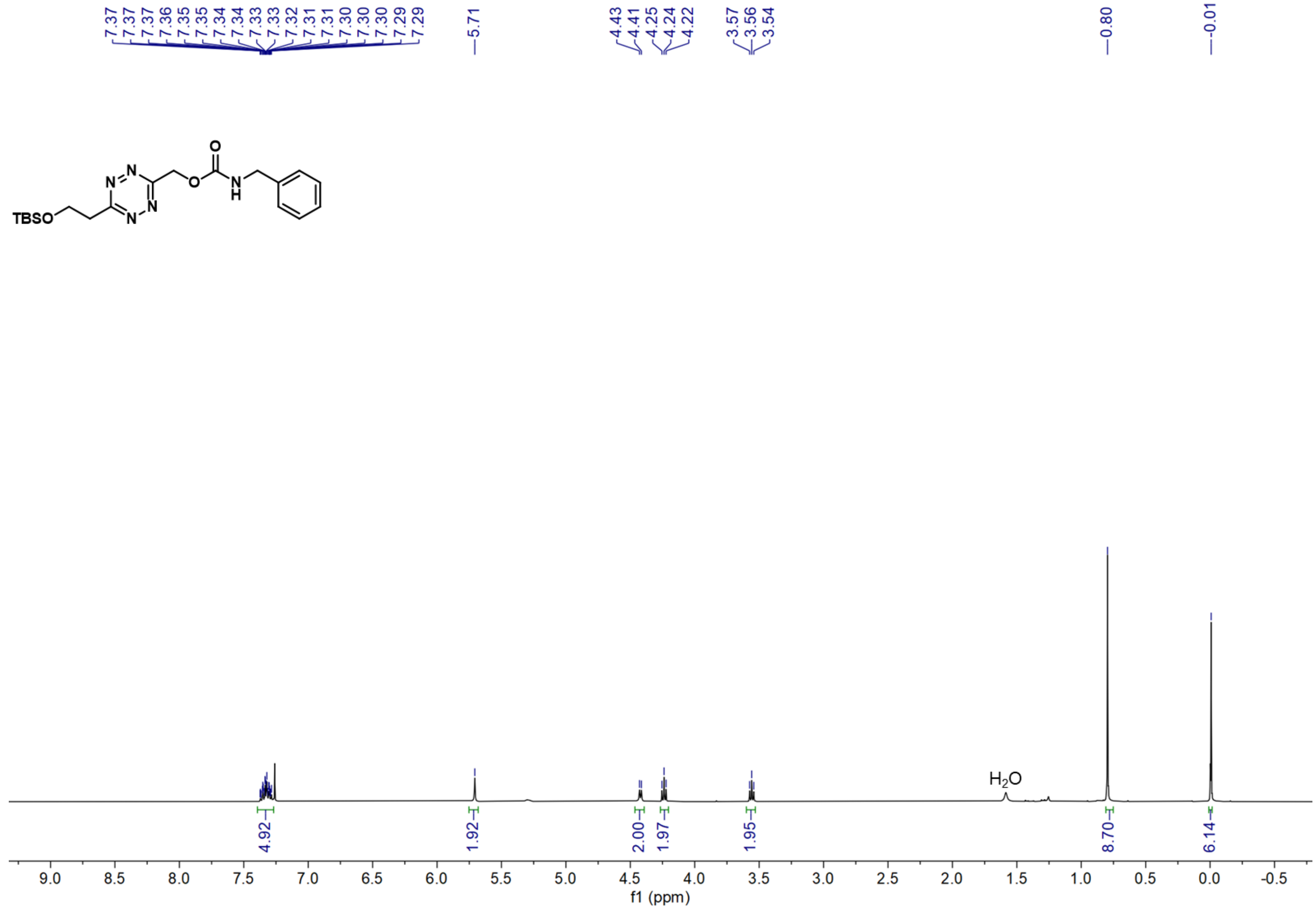

Supplementary Fig. 96. **^{1}H NMR spectra of compound 6a**.

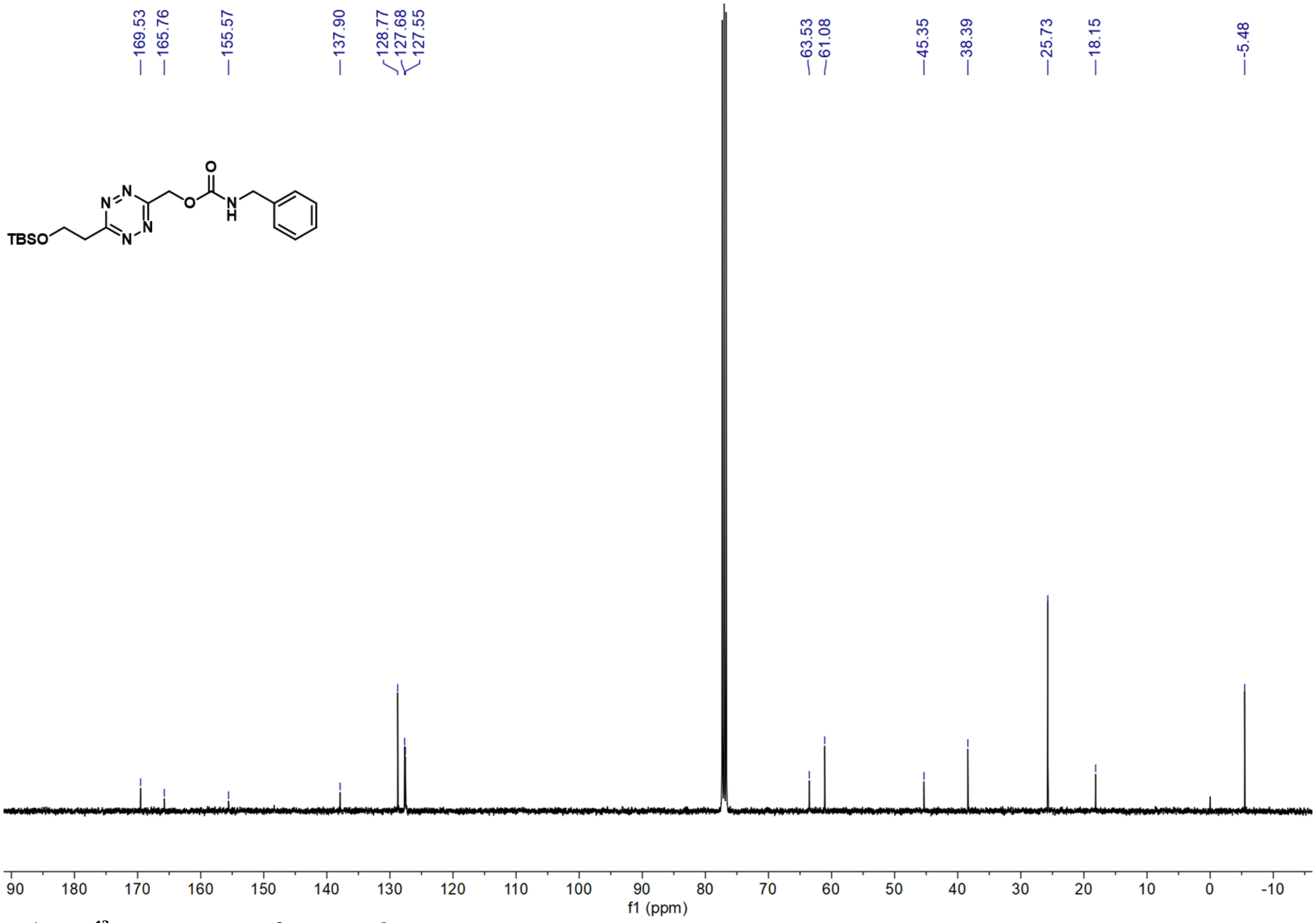

Supplementary Fig. 97. **^{13}C NMR spectra of compound 6a**.

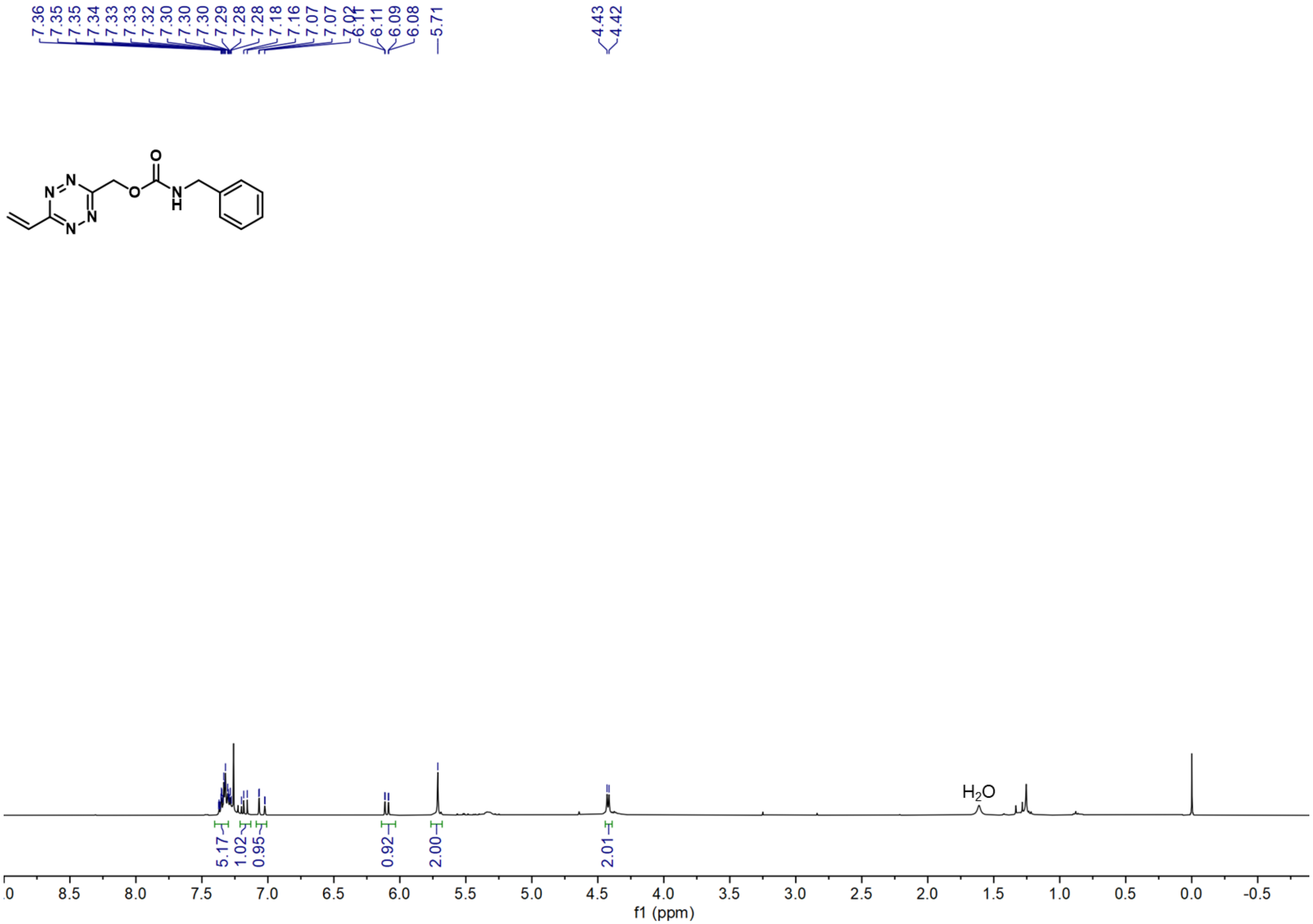

Supplementary Fig. 98. **^{1}H NMR spectra of compound 6**.

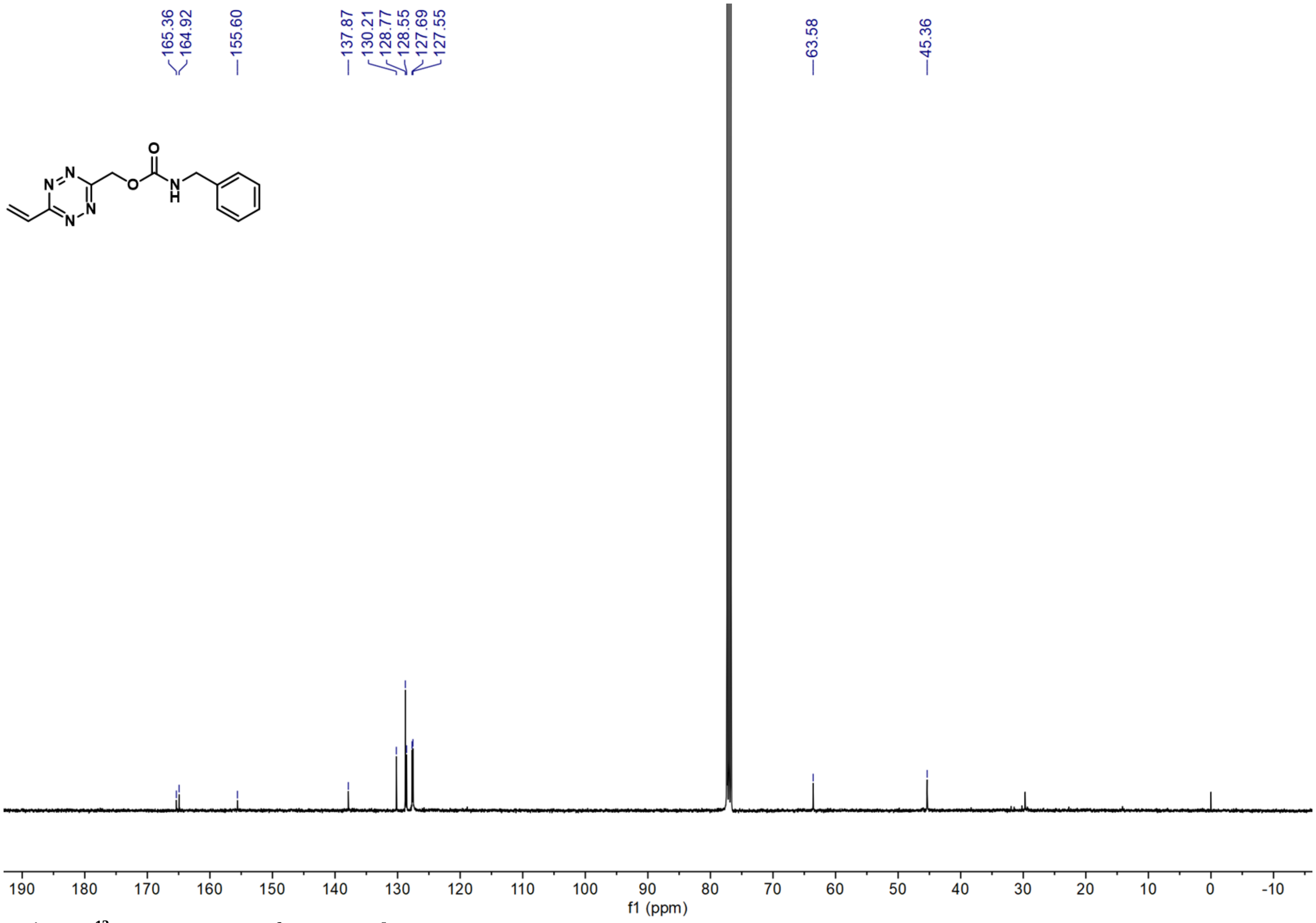

Supplementary Fig. 99. **^{13}C NMR spectra of compound 6**.

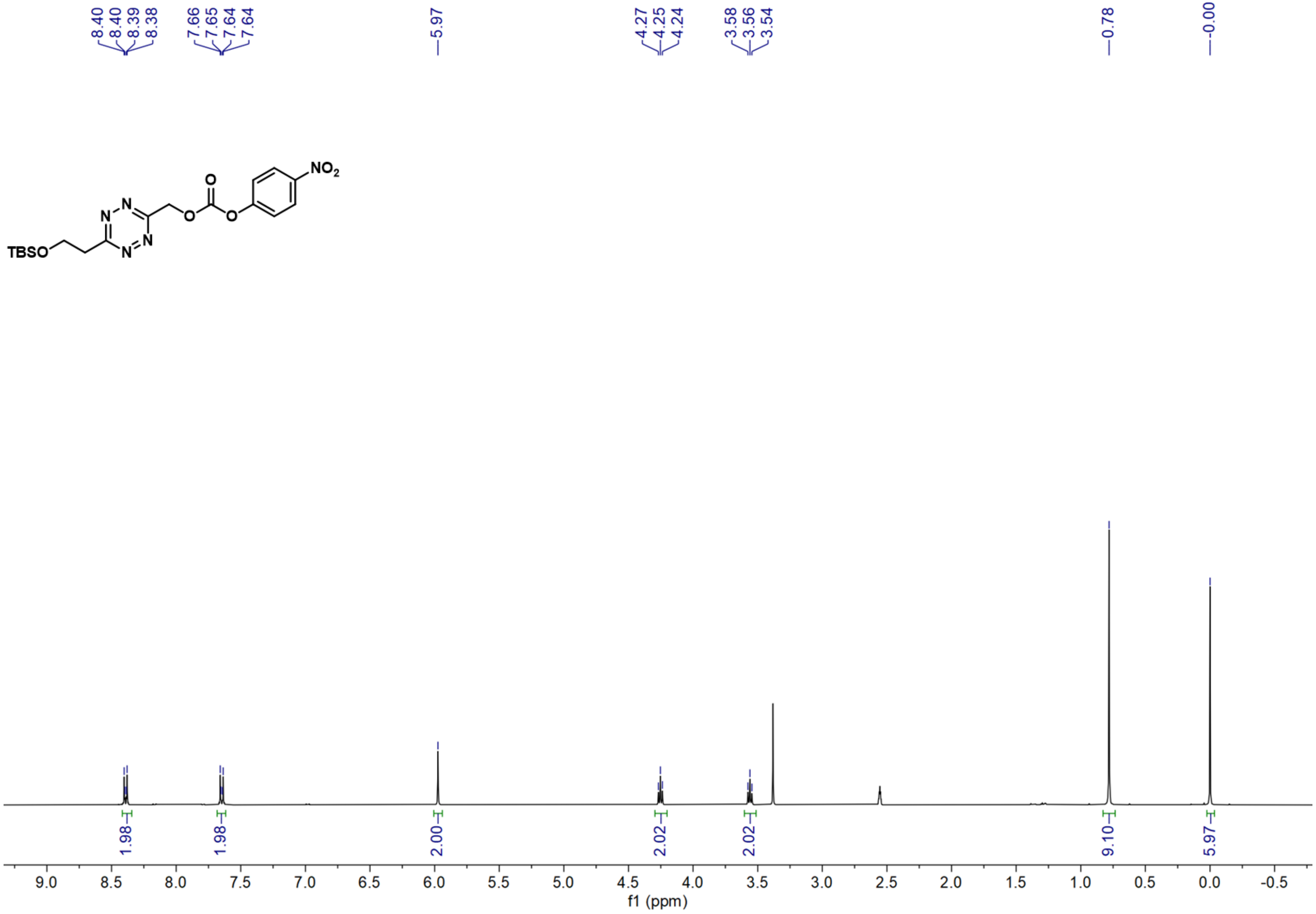

Supplementary Fig. 100. ^{1}H NMR spectra of compound 7a.

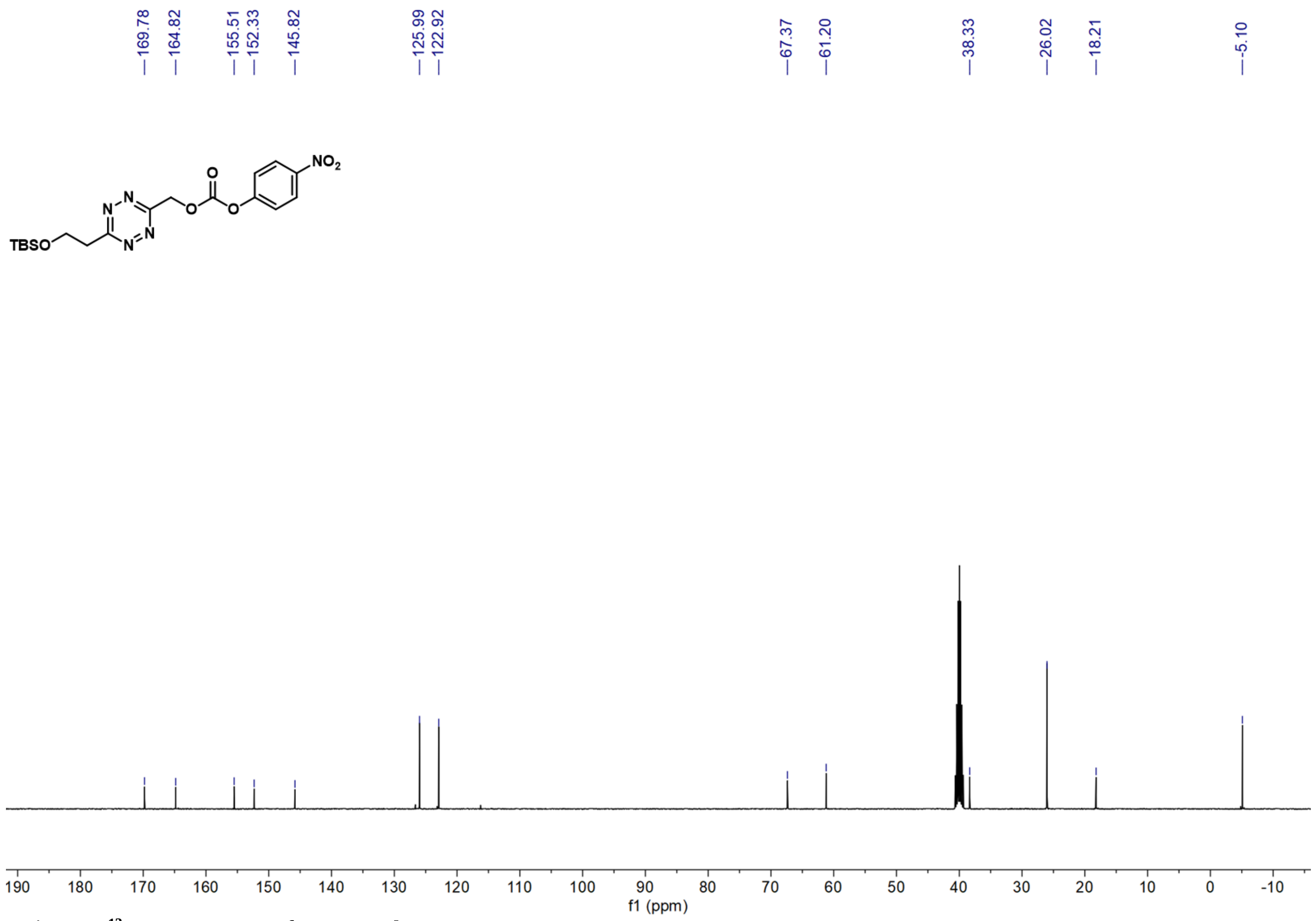

Supplementary Fig. 101. ^{13}C NMR spectra of compound 7a.

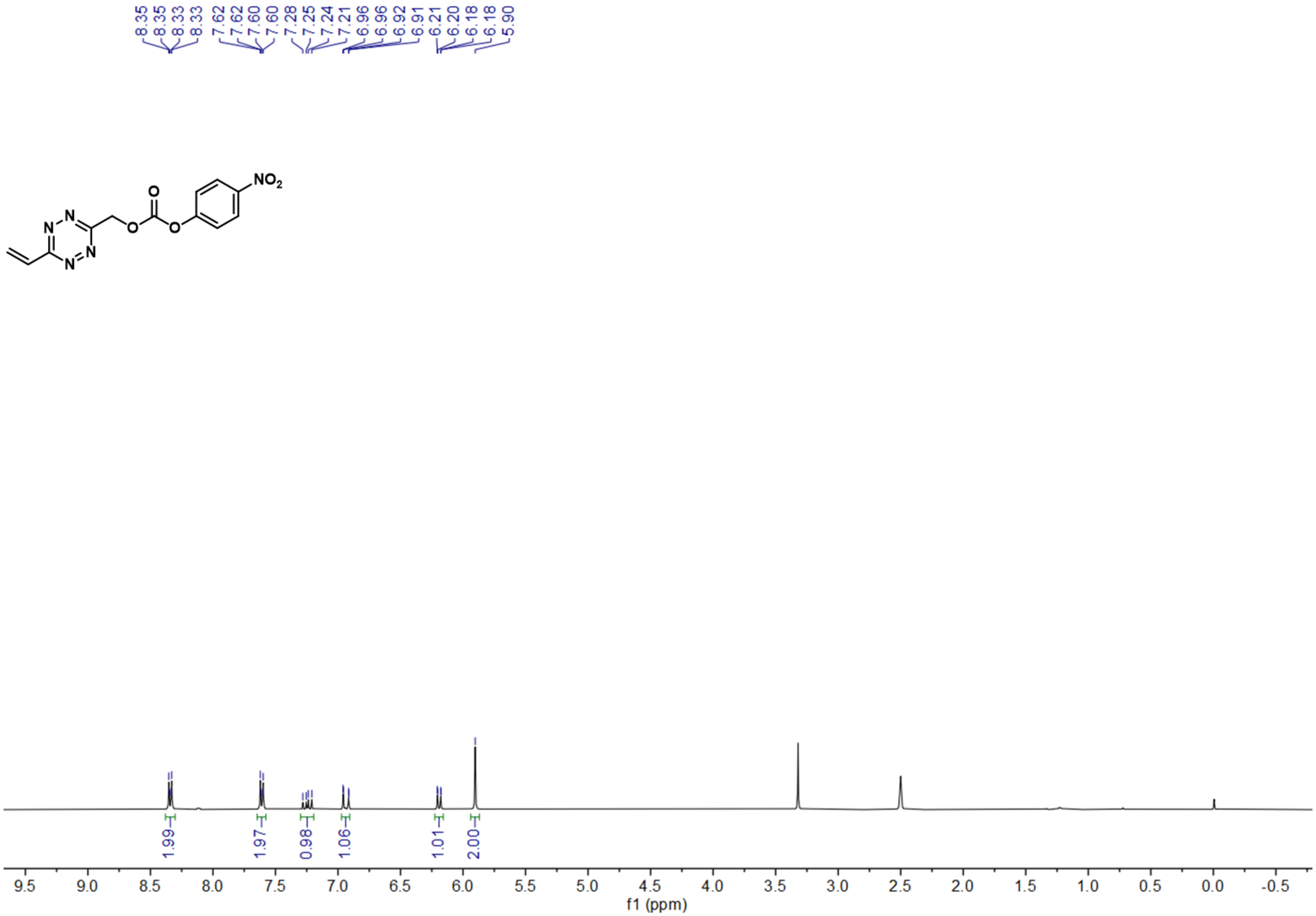

Supplementary Fig. 102. **^{1}H NMR spectra of compound 7b**.

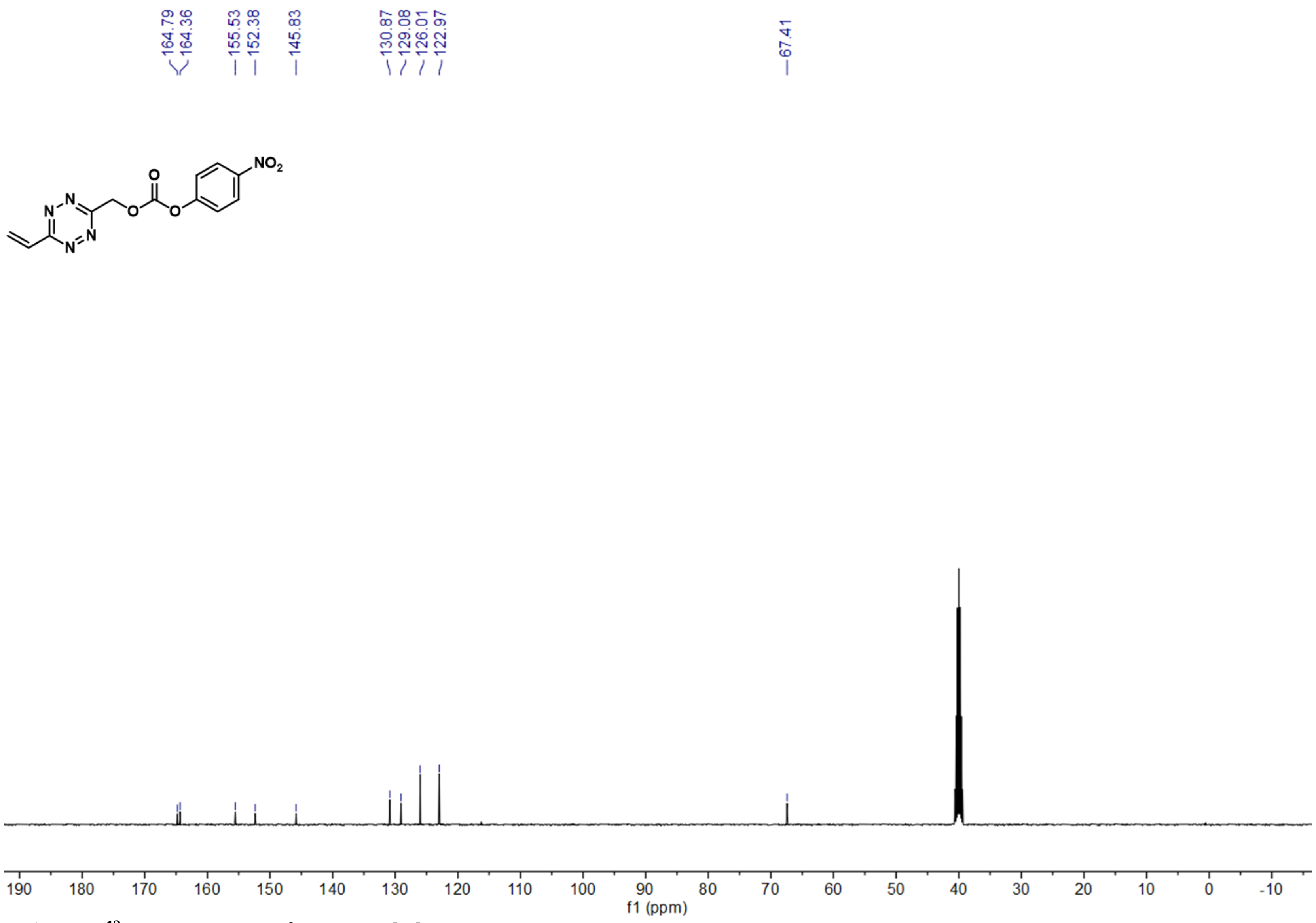

Supplementary Fig. 103. ^{13}**C NMR spectra of compound 7b**.

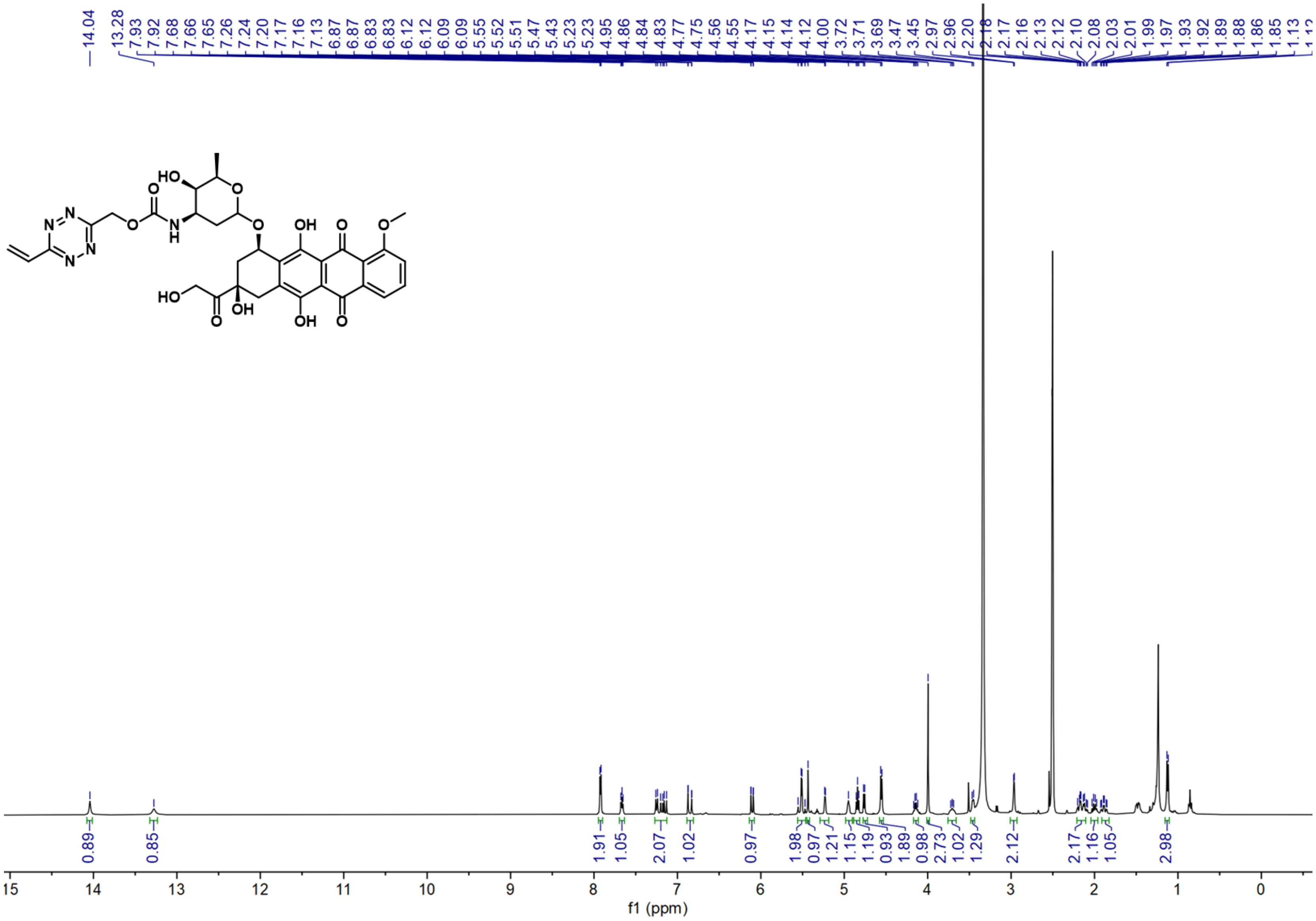

Supplementary Fig. 104. ^{1}H NMR spectra of compound 7.

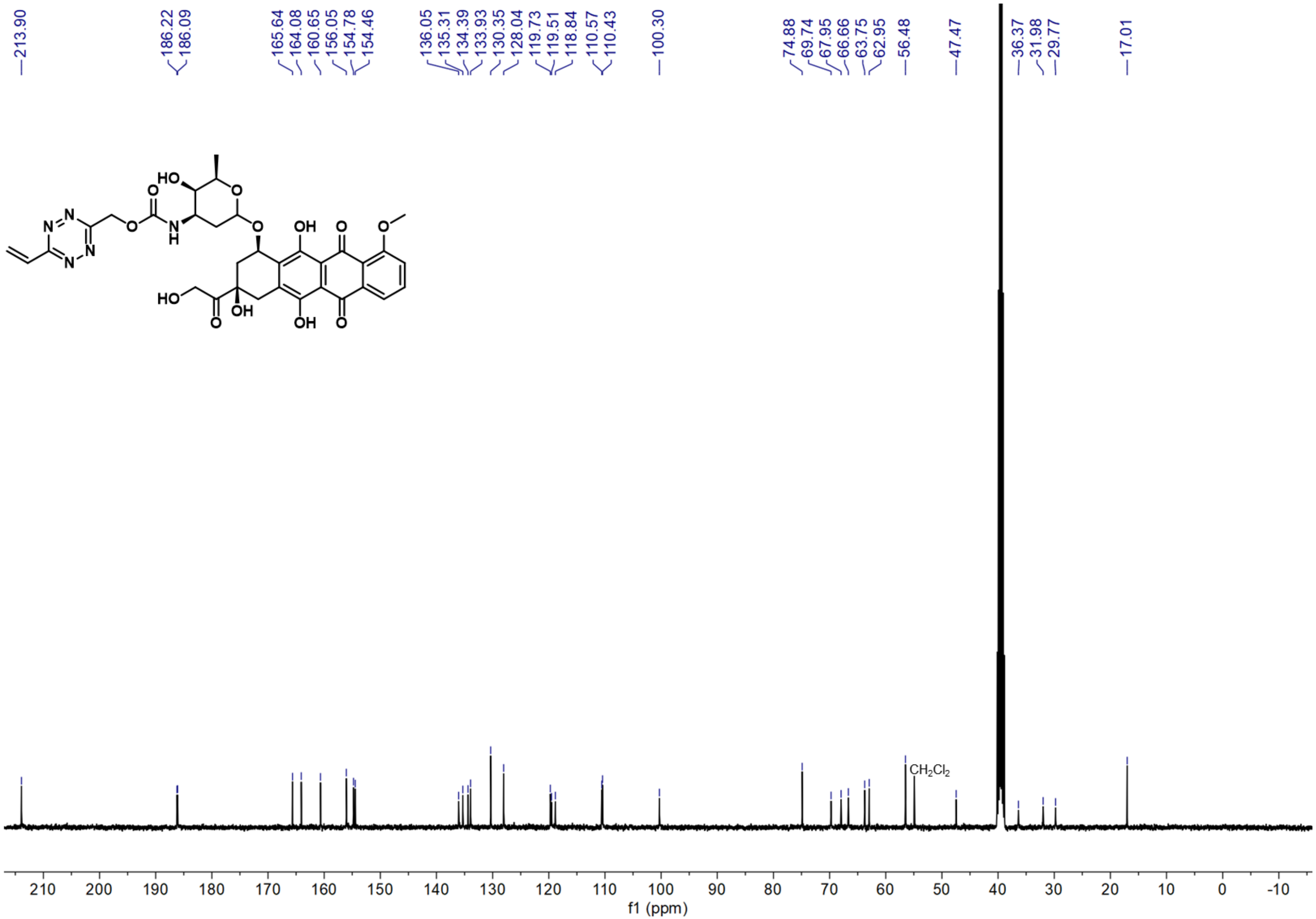

Supplementary Fig. 105. **^{13}C NMR spectra of compound 7**.

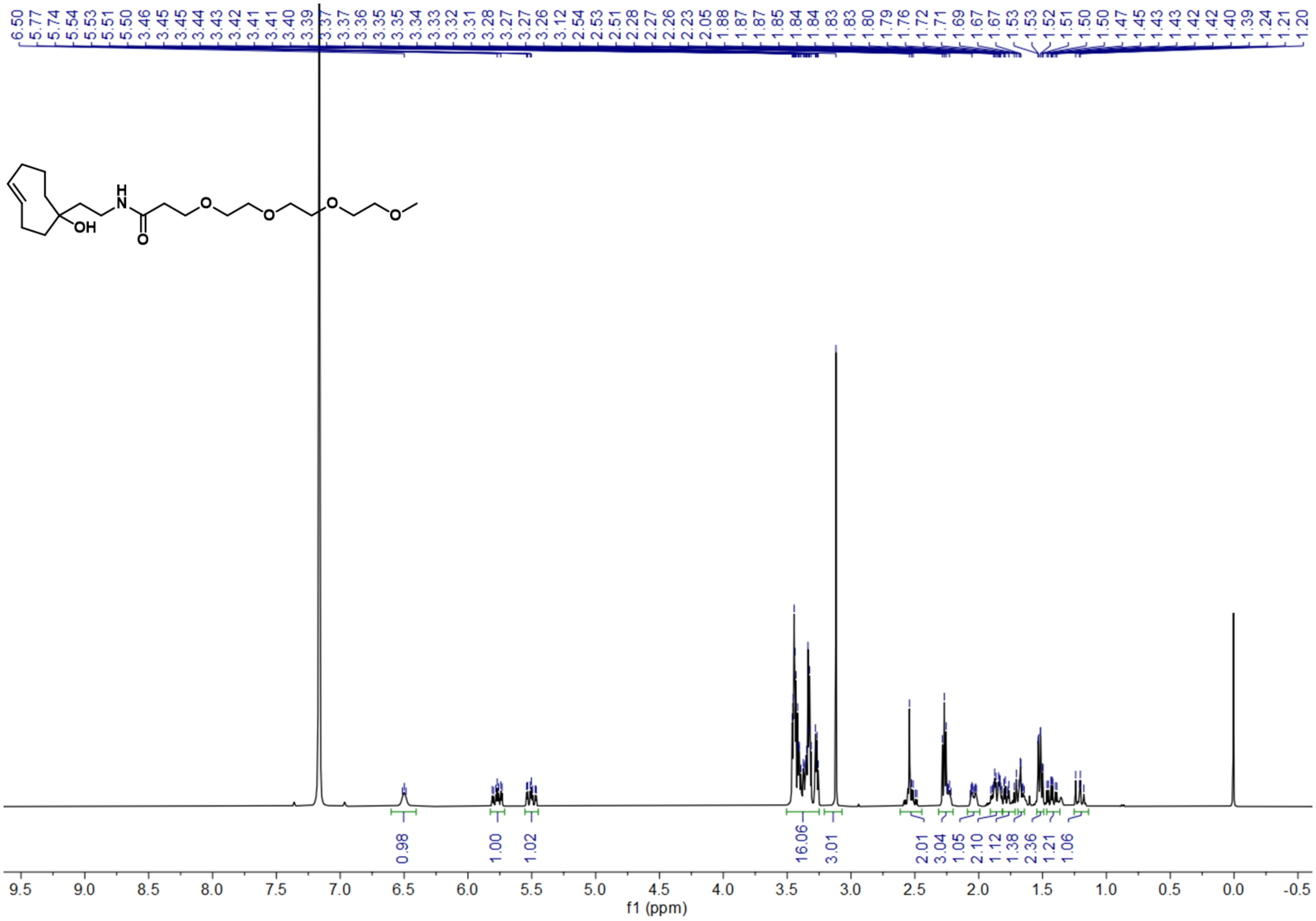

Supplementary Fig. 106. **^{1}H NMR spectra of compound TCO-P$_4$.**

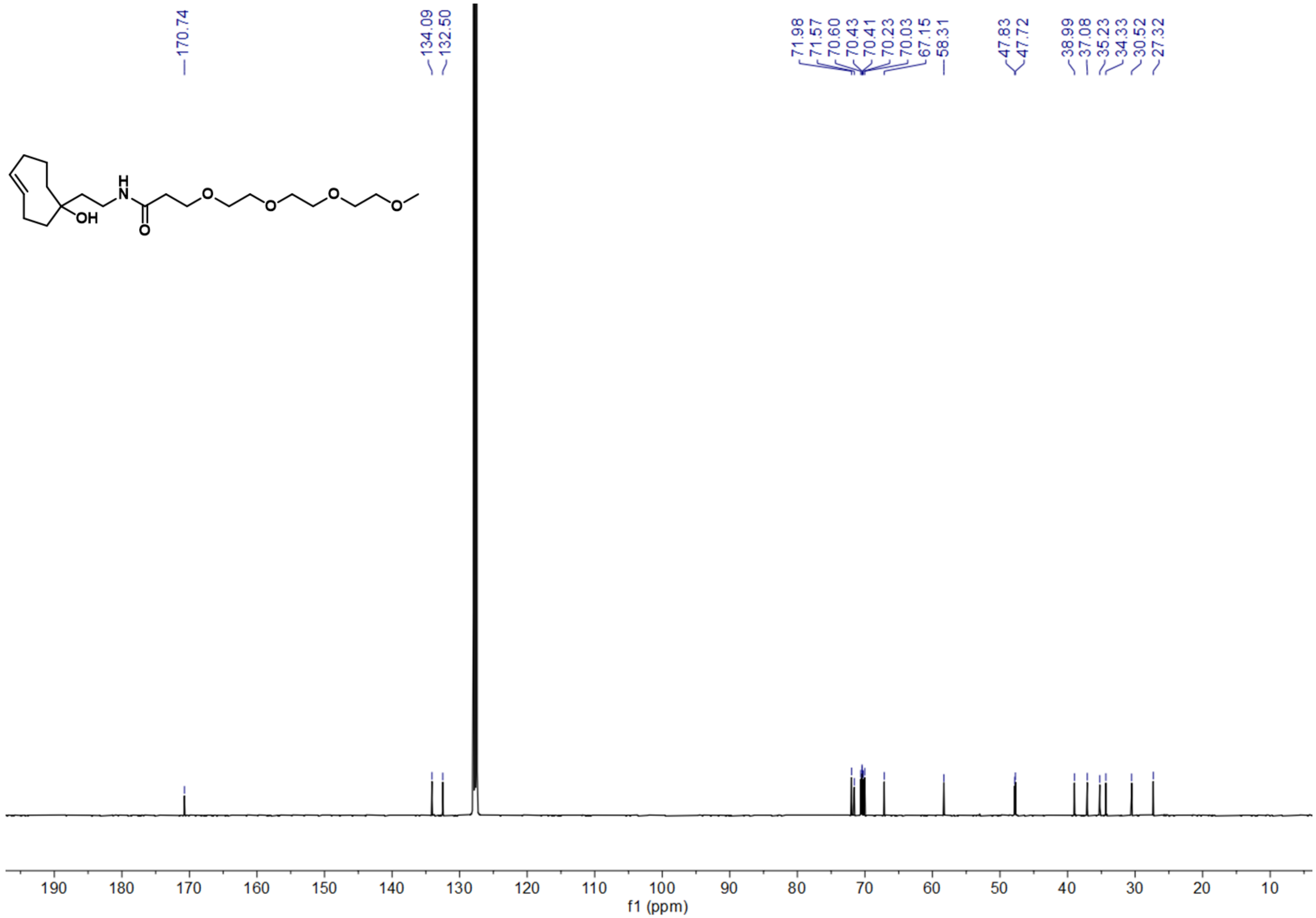

Supplementary Fig. 107. **^{13}C NMR spectra of compound TCO-P$_4$**.

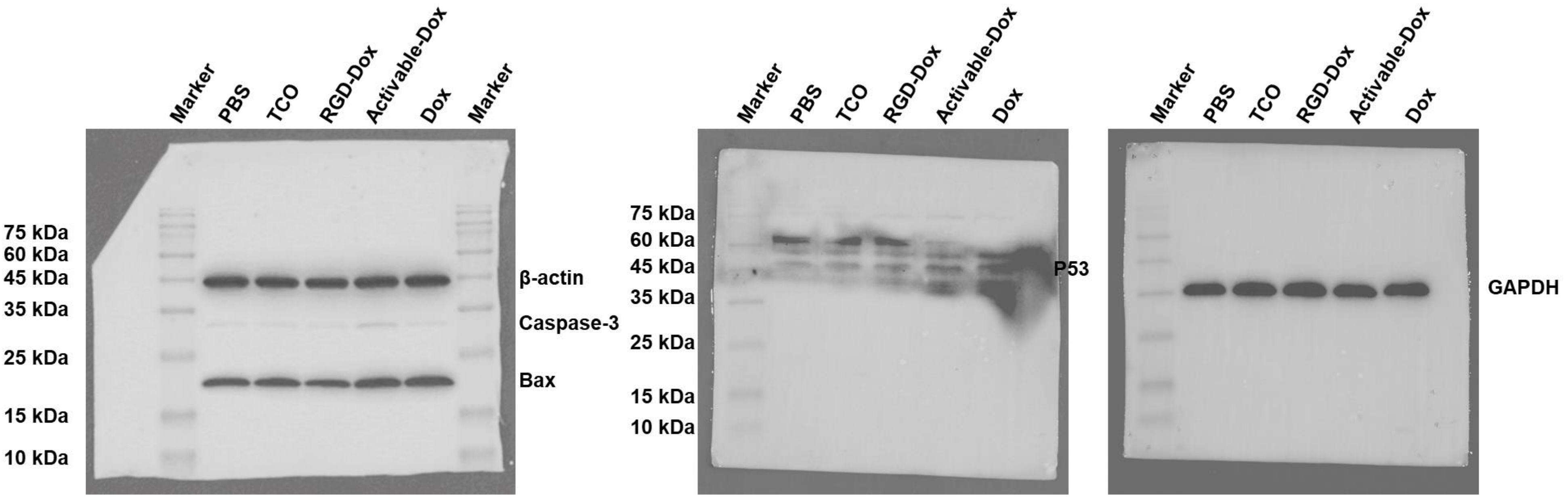

Supplementary Fig. 108. **Uncropped scans of western blotting analysis**.

Supplementary References

1. Pigga, J. E., et al. General, Divergent Platform for Diastereoselective Synthesis of *trans*‐Cyclooctenes with High Reactivity and Favorable Physiochemical Properties. *Angew. Chem., Int. Ed.* **60**, 14975−14980 (2021).
2. Tessier, R., et al. "Doubly Orthogonal" Labeling of Peptides and Proteins. *Chem* **5**, 2243−2263 (2019).
