# Supplementary material for: An all-in-one tetrazine reagent for cysteine-selective labeling and bioorthogonal activable prodrug construction
Source: Nat Commun. 2024 Apr 2;15:2831. doi: 10.1038/s41467-024-47188-6 (PMC10987521; doi:10.1038/s41467-024-47188-6)
Supplement: Supplementary file 3 — Reporting Summary [file 41467_2024_47188_MOESM3_ESM.pdf]

nature portfolio

Corresponding author(s): Haoxing Wu

Last updated by author(s): Mar 14, 2024

Reporting Summary

Nature Portfolio wishes to improve the reproducibility of the work that we publish. This form provides structure for consistency and transparency in reporting. For further information on Nature Portfolio policies, see our Editorial Policies and the Editorial Policy Checklist.

Statistics

For all statistical analyses, confirm that the following items are present in the figure legend, table legend, main text, or Methods section.

n/a	Confirmed	
☐	☒	The exact sample size (*n*) for each experimental group/condition, given as a discrete number and unit of measurement
☐	☒	A statement on whether measurements were taken from distinct samples or whether the same sample was measured repeatedly
☐	☒	The statistical test(s) used AND whether they are one- or two-sided<br>*Only common tests should be described solely by name; describe more complex techniques in the Methods section.*
☒	☐	A description of all covariates tested
☒	☐	A description of any assumptions or corrections, such as tests of normality and adjustment for multiple comparisons
☐	☒	A full description of the statistical parameters including central tendency (e.g. means) or other basic estimates (e.g. regression coefficient) AND variation (e.g. standard deviation) or associated estimates of uncertainty (e.g. confidence intervals)
☐	☒	For null hypothesis testing, the test statistic (e.g. F, t, r) with confidence intervals, effect sizes, degrees of freedom and P value noted<br>*Give P values as exact values whenever suitable.*
☒	☐	For Bayesian analysis, information on the choice of priors and Markov chain Monte Carlo settings
☒	☐	For hierarchical and complex designs, identification of the appropriate level for tests and full reporting of outcomes
☒	☐	Estimates of effect sizes (e.g. Cohen's *d*, Pearson's *r*), indicating how they were calculated

Our web collection on statistics for biologists contains articles on many of the points above.

Software and code

Policy information about availability of computer code

Data collection	NMR spectra were recorded on a Bruker NMR spectrometer (1H, 400 MHz; 13C, 101 MHz),<br>High-resolution mass spectra (HRMS) were recorded on a quadrupole time-of-flight mass spectrometer (micrO-TOF-QII, Bruker) with electrospray ionization (ESI),<br>High-performance liquid chromatography-mass spectrometry (HPLC–MS) for each peptide was recorded on a Bruker micro-TOF-QII time of flight mass spectrometer with electrospray ionization (ESI),<br>Ultraviolet–visible (UV–Vis) absorption spectra were recorded on a Quawell scientific Q6000+ microvolume spectrophotometer,<br>Fluorescence spectra were acquired on a wavelength-calibrated FluoroMax-4 fluorometer (Horiba Jobin Yvon, Kyoto, Japan),<br>DLS analyses and zeta potential values were determined using a Nano ZS ZEN 3690 instrument (Malvern Instruments),<br>Transmission electron microscope (TEM) images were obtained using a Talos FEI 200 instrument (Thermo Scientific),<br>Fluorescence images were obtained using a confocal laser scanning microscope (Zeiss 880, 63x magnification).
Data analysis	Fluorescence images were analyzed with ZEN blue software,<br>Data were analyzed using Origin Pro 2015 and Graphpad Prism 8.

For manuscripts utilizing custom algorithms or software that are central to the research but not yet described in published literature, software must be made available to editors and reviewers. We strongly encourage code deposition in a community repository (e.g. GitHub). See the Nature Portfolio guidelines for submitting code & software for further information.

Data

Policy information about availability of data

All manuscripts must include a data availability statement. This statement should provide the following information, where applicable:

- Accession codes, unique identifiers, or web links for publicly available datasets
- A description of any restrictions on data availability
- For clinical datasets or third party data, please ensure that the statement adheres to our policy

The authors declare that the data supporting the findings of this study are available with the paper and its Supplementary information files. Source data are provided with this paper.

Research involving human participants, their data, or biological material

Policy information about studies with human participants or human data. See also policy information about sex, gender (identity/presentation), and sexual orientation and race, ethnicity and racism.

Reporting on sex and gender	n/a
Reporting on race, ethnicity, or other socially relevant groupings	n/a
Population characteristics	n/a
Recruitment	n/a
Ethics oversight	n/a

Note that full information on the approval of the study protocol must also be provided in the manuscript.

Field-specific reporting

Please select the one below that is the best fit for your research. If you are not sure, read the appropriate sections before making your selection.

- [x] Life sciences
- [ ] Behavioural & social sciences
- [ ] Ecological, evolutionary & environmental sciences

For a reference copy of the document with all sections, see nature.com/documents/nr-reporting-summary-flat.pdf

Life sciences study design

All studies must disclose on these points even when the disclosure is negative.

Sample size	The sample sizes of in vivo anticancer efficacy (number of mice for each group) are 5. No statistical methods were used to predetermine sample sizes. Sample size was determined based on previous published studies using similar methodologies (https://doi.org/10.1038/s41467-024-45072-x, https://doi.org/10.1038/s41467-023-44308-6, https://doi.org/10.1038/s41467-023-37253-x). All sample sizes are listed in the corresponding figure legends or on the figures.
Data exclusions	No data were excluded from the analyses.
Replication	The number of replicated experiments are indicated in the figure legends and supplementary information.
Randomization	Mice were randomly divided into five groups for the in vivo anticancer efficacy.
Blinding	The evaluation of histopathology of HE-stained and TUNEL-stained tissue sections were performed in a blinded fashion. In the experiments of in vivo anticancer efficacy , no blinding was used, because after the animals were randomly divided into five groups, researchers need to know the exact experimental group in order to administer drugs correctly.

Reporting for specific materials, systems and methods

We require information from authors about some types of materials, experimental systems and methods used in many studies. Here, indicate whether each material, system or method listed is relevant to your study. If you are not sure if a list item applies to your research, read the appropriate section before selecting a response.

Materials & experimental systems

n/a	Involved in the study
	☒ Antibodies
	☒ Eukaryotic cell lines
☒	Palaeontology and archaeology
	☒ Animals and other organisms
☒	Clinical data
☒	Dual use research of concern
☒	Plants

Methods

n/a	Involved in the study
☒	ChIP-seq
	☒ Flow cytometry
☒	MRI-based neuroimaging

Antibodies

Antibodies used	All the antibodies were obtained from Abcam Ltd (Cambridge, UK):<br>Anti-p53 antibody, Rabbit polyclonal (ab131442, 1:500)<br>Anti-Bax antibody, Rabbit monoclonal [E63] (ab32503, 1:4000)<br>Anti-Caspase-3 antibody, Rabbit monoclonal [EPR18297] (ab184787, 1:2000)<br>Anti-β-actin antibody, Rabbit polyclonal (ab8227, 1:4000)<br>Anti-GAPDH antibody, Rabbit monoclonal [EPR16891] (ab181602, 1:10000)<br>Goat anti-Rabbit IgG HL (HRP, ab205718, 1:10000)
Validation	All the antibodies used in this study were commercially available and validated by manufacturers as described on the following web sites:<br>Anti-p53 antibody (ab131442), https://www.abcam.com/products/primary-antibodies/p53-antibody-ab131442.html<br>Anti-Bax antibody (ab32503), https://www.abcam.com/products/primary-antibodies/bax-antibody-e63-ab32503.html<br>Anti-Caspase-3 antibody (ab184787), https://www.abcam.com/products/primary-antibodies/caspase-3-antibody-epr18297-ab184787.html<br>Anti-β-actin antibody (ab8227), https://www.abcam.com/products/primary-antibodies/beta-actin-antibody-ab8227.html<br>Anti-GAPDH antibody (ab181602), https://www.abcam.com/products/primary-antibodies/gapdh-antibody-epr16891-loading-control-ab181602.html<br>Goat anti-Rabbit IgG HL (HRP, ab205718), https://www.abcam.com/products/secondary-antibodies/goat-rabbit-igg-hl-hrp-ab205718.html

Eukaryotic cell lines

Policy information about cell lines and Sex and Gender in Research

Cell line source(s)	Human ovarian cancer SKOV3 cells (CL-0215), Human breast cancer MCF-7 cells (CL-0149), Human brain glioma U87 cells (CL-0238), Mouse melanoma B16F10 cells (CL-0319), Human hepatic stellate LX2 cells (CL-0560), Human hepatocellular carcinomas HepG2 cells (CL-0103) and Human breast cancer MDA-MB-231 cells (CL-0150) were kindly provided by Procell Life Science & Technology Co.,Ltd.
Authentication	Authentication of all cells was conducted via short tandam repeat (STR) profiling in Procell Life Science & Technology Co.,Ltd.
Mycoplasma contamination	Cells were not tested for mycoplasma contamination.
Commonly misidentified lines (See ICLAC register)	None.

Animals and other research organisms

Policy information about studies involving animals; ARRIVE guidelines recommended for reporting animal research, and Sex and Gender in Research

Laboratory animals	The animals in this study are male BALB/c nude mice (5 weeks old, 20 g) which were purchased from Beijing HFK Bioscience (Beijing, China). Mice were housed at ambient temperature of 23±2 °C and relative humidities of 55%±2% in a specific pathogen-free environment with a 12-h light/dark cycle.
Wild animals	The study did not involve wild animals.
Reporting on sex	The experiment was designed without considering the sex of the mice, and male mice were selected to ensure gender uniformity which could control the experimental variables.
Field-collected samples	The study did not involve samples collected from the field.
Ethics oversight	All animal experiments were approved by the Committee for Animal Care and Use and the Ethics Committee of West China Hospital, Sichuan University (approval number: 20230625002).

Note that full information on the approval of the study protocol must also be provided in the manuscript.

Plants

Seed stocks	n/a
Novel plant genotypes	n/a
Authentication	n/a

Flow Cytometry

Plots

Confirm that:

- [x] The axis labels state the marker and fluorochrome used (e.g. CD4-FITC).
- [x] The axis scales are clearly visible. Include numbers along axes only for bottom left plot of group (a 'group' is an analysis of identical markers).
- [x] All plots are contour plots with outliers or pseudocolor plots.
- [x] A numerical value for number of cells or percentage (with statistics) is provided.

Methodology

Sample preparation	B16F10 cells were seeded onto 6-well cell culture clusters and incubated for 48 hours. After the incubation period, the cells were exposed to RGD-Dox (10 μM) at 37 °C for different durations. Subsequently, the cells were trypsinized and underwent three rinses with PBS. Flow cytometry analysis was employed to assess the intrinsic fluorescence intensity of Dox with an excitation/emission wavelength of 488/610 nm.
Instrument	Fortessa, BD, USA
Software	FlowJo_v10.8.1
Cell population abundance	At least 10000 cells were used for flow cytometric analysis.
Gating strategy	In general, cells were initially gated based on forward scatter (FSC) and side scatter (SSC). Singlet cells were typically gated using FSC height (FSC-H) and FSC area (FSC-A). We have included this gating strategy for flow cytometry experiments in the Supplementary Information.

- [x] Tick this box to confirm that a figure exemplifying the gating strategy is provided in the Supplementary Information.
